# Genome-Wide Identification of Calcium Dependent Protein Kinase Gene Family in Plant Lineage Shows Presence of Novel D-x-D and D-E-L Motifs in EF-Hand Domain

**DOI:** 10.3389/fpls.2015.01146

**Published:** 2015-12-24

**Authors:** Tapan K. Mohanta, Nibedita Mohanta, Yugal K. Mohanta, Hanhong Bae

**Affiliations:** ^1^School of Biotechnology, Yeungnam UniversityGyeongsan, South Korea; ^2^Department Of Biotechnology, North Orissa UniversityBaripada, India; ^3^Department Of Botany, North Orissa UniversityBaripada, India

**Keywords:** CPK, CaM, CBL, calcium signaling, evolution, EF-hands

## Abstract

Calcium ions are considered ubiquitous second messengers in eukaryotic signal transduction pathways. Intracellular Ca^2+^ concentration are modulated by various signals such as hormones and biotic and abiotic stresses. Modulation of Ca^2+^ ion leads to stimulation of calcium dependent protein kinase genes (*CPKs*), which results in regulation of gene expression and therefore mediates plant growth and development as well as biotic and abiotic stresses. Here, we reported the *CPK* gene family of 40 different plant species (950 *CPK* genes) and provided a unified nomenclature system for all of them. In addition, we analyzed their genomic, biochemical and structural conserved features. Multiple sequence alignment revealed that the kinase domain, auto-inhibitory domain and EF-hands regions of regulatory domains are highly conserved in nature. Additionally, the EF-hand domains of higher plants were found to contain four D-x-D and two D-E-L motifs, while lower eukaryotic plants had two D-x-D and one D-x-E motifs in their EF-hands. Phylogenetic analysis showed that *CPK* genes are clustered into four different groups. By studying the *CPK* gene family across the plant lineage, we provide the first evidence of the presence of D-x-D motif in the calcium binding EF-hand domain of CPK proteins.

## Introduction

Plants are constantly challenged by different environmental factors that guide regulation of various physiological and developmental programs in a systematic manner (Choi et al., 2014). Plants sense and process environmental stimuli such as light, temperature, water and nutrients to enable normal growth and development (Krouk et al., 2011; Davidson and Gu, 2012; Lawson et al., 2014; Matos et al., 2014). Plant cells are able to respond to external stimuli in a time dependent manner, after which they process the information received. This is followed by signal transduction and finally appropriate physiological responses (Asano et al., 2005; Clouse, 2011; Tena et al., 2011; Huang et al., 2012; Boudsocq and Sheen, 2013; Wasternack and Hause, 2013). The propagation and amplification of signals involve rapid changes in cellular and sub-cellular levels of signals by second messengers that relay information to downstream effector molecules such as enzymes, transcription factors, and cytoskeletal proteins, which are responsible for changes in gene expression, metabolic activity and developmental patterns (Mohanta et al., 2012; Kanchiswamy et al., 2013). Among these messengers, calcium dependent protein kinases (CPKs) are the most important enzymes responsible for a diverse array of plant growth and development, as well as biotic and abiotic stress responses (Luan, 2009; Kanchiswamy et al., 2013; Mohanta et al., 2015b).

Calcium ion is considered an ubiquitous second messenger in eukaryotic signal transduction pathways (Hamel et al., 2014). Intracellular Ca^2+^ concentrations are modulated by various signals (e.g., hormones, biotic and abiotic stresses) and play significant roles in plant growth and development and stress responses (Dodd et al., 2010; Reddy et al., 2011; Mohanta and Bae, 2015). CPKs are considered novel calcium sensors that act as crucial mediators in response to diverse stress conditions (Cheng et al., 2002; Schulz et al., 2013). The genes encoding CPKs are found in plants as multi-gene family members (Harmon et al., 2001). The CPKs are classified as serine/threonine protein kinases that contain five domains: (i) N-terminal variable domain, (ii) kinase domain, (iii) auto-inhibitory domain, (iv) regulatory domain, and (v) C-terminal variable domain (Harmon et al., 2001). The regulatory domain contains four calcium binding EF-hands, and the conserved glutamic acid (E) and aspartate (D) amino acids present in the EF-hands act as an important calcium sensor (Day et al., 2002; Hrabak et al., 2003). The EF-hands, which act in pairs, are divided into N-and C-terminal EF-hand pairs. The EF-hand motif is highly conserved and contains a 12 amino acids helix-loop-helix structure that coordinates the calcium ion. The EF-hand module binds Ca^2+^ ion in the loop and undergoes conformational changes that result in the exposure of hydrophobic pockets, which facilitates protein-protein interactions with a variety of downstream interacting partners (Harmon et al., 2000; Sulmann et al., 2014). The N- and C-terminal domains of CPKs are highly variable in nature and contain myristoylation and palmitoylation sites for sub-cellular targeting, whereas the kinase domain is catalytic in nature and contains an ATP binding site (Ito et al., 2010). While CPKs are regulated by diverse mechanisms, sensitivity to calcium ion can be influenced by the type of substrate, and each isoform of CPKs responds to a specific set of calcium signals.

There are several gene families that act as potential calcium sensors in plants including calcium dependent protein kinase (*CPK*), calmodulin (*CaM*), calmodulin like (*CML*), calcineurin B-like (*CBL*), and CPK related kinase (*CRK*) (Hrabak et al., 2003). Among these calcium sensor genes, CPKs are unique in that they contain both a kinase catalytic domain and an EF-hand containing calcium binding domain, which leads to signaling and sensor activity in a single gene product (Harmon et al., 2000). The fusion of the kinase domain with an EF-hand containing regulatory domain probably arose via early fusion of an upstream protein kinase with downstream CaM to conduct immediate and efficient downstream phosphorylation of target proteins.

To date, there has been little information available regarding the unified nomenclature, genomics, biochemistry and conserved structures of *CPK* genes in plants. Therefore, in this study, we conducted large scale identification of *CPK* gene family members using 40 different plant species (950 *CPK* genes). Additionally, we provided a unified nomenclature system and analyzed the conserved structural and functional aspects of *CPKs* (Ouyang et al., 2007; Goodstein et al., 2012; Lamesch et al., 2012; Nystedt et al., 2013).

## Materials and methods

### Identification of CPK genes

To understand the signature motif of the CPK gene family in plants, genome-wide identification of *CPK* gene family members of 40 plant species was conducted. The *CPK* genes from *Arabidopsis thaliana* were downloaded from “The Arabidopsis Information Resources” (TAIR) database (Lamesch et al., 2012), while the *CPK* genes from rice were downloaded from the “rice Genome Annotation Resources” (http://rice.plantbiology.msu.edu/) database (Ouyang et al., 2007). The CPK protein sequences from *Arabidopsis thaliana* and *Oryza sativa* were used as search queries in the publicly available phytozome database (http://www.phytozome.net/) (Goodstein et al., 2012) and *P. abies* genome database (http://congenie.org/start) (Nystedt et al., 2013) to identify the CPK genes from other plant species. In total, 40 species were included in this study (Table 1). The BLASTP search was conducted to identify the *CPK* gene family members of unknown species by taking the protein sequences of AtCPKs and OsCPKs. All CPK proteins of the 40 species were analyzed using the SCAN PROSITE software with default parameters to confirm the presence of all the domains of CPKs (de Castro et al., 2006). Only CPK proteins that contained the kinase domain, auto-inhibitory domain, and regulatory domain with four calcium binding EF-hands was considered for further investigation. All the CPK proteins identified from these approaches were again subjected to BLASTP searches against the TAIR and rice genome annotation database to confirm the BLAST hits with *CPK* genes of *Arabidopsis thaliana* and *Oryza sativa*. The palmitoylation sites of all CPK proteins were identified by CSS Palm software version 2.0 (Ren et al., 2008). The molecular masses of all CPK proteins were predicted using protein calculator version 3.3 (http://www.scripps.edu/~cdputnam/protcalc.html).

**Table 1:** **Genome size of different plant species used in this study and number of CPK genes present in their respective genomes**.

**Sl. No**	**Name of species**	**Type of organism**	**Genome size (Mbs)**	**Total no. of loci/genome**	**Total no. of CPK genes/genome**
1	*Aquilegia coerulea*	Dicot	302	24823	16
2	*Arabidopsis thaliana*	Dicot	135	27416	34
3	*Brachipodium distachyon*	Monocot	272	31694	27
4	*Brassica rapa*	Dicot	283.8	40492	49
5	*Capsella rubella*	Dicot	134.8	26521	32
6	*Carica papaya*	Dicot	135	27332	15
7	*Chlamydomonas reinhardtii*	Lower eukaryote	111.1	17741	14
8	*Citrus clememtina*	Dicot	301.4	24533	26
9	*Citrus sinensis*	Dicot	319	25376	24
10	*Coccomyxa subellipsoidea*	Lower eukaryote	49	9629	2
11	*Cucumis sativus*	Dicot	203	21494	18
12	*Eucalyptus grandis*	Dicot	691	36376	22
13	*Fragaria vesca*	Dicot	240	32831	14
14	*Glycine max*	Dicot	975	54175	41
15	*Gossipium raimondi*	Dicot	761.4	37505	40
16	*Linum usitatissimum*	Dicot	318.3	43471	47
17	*Malus domestica*	Dicot	881.3	63514	28
18	*Manihot esculenta*	Dicot	532.5	34085	26
19	*Medicago truncatula*	Dicot	241	50894	11
20	*Micromonas pusila CCMP1545*	Lower eukaryote	22	10660	2
21	*Mimulus guttatus*	Dicot	321.7	26718	25
22	*Oryza sativa*	Monocot	372	39049	30
23	*Ostreococcus lucimarinus*	Lower eukaryote	13.2	7796	3
24	*Panicum virgatum*	Monocot	1358	98007	53
25	*Phaseolus vulgaris*	Dicot	521.1	27197	25
26	*Physcomitrella patens V.13*	Bryophyte	480	33362	25
27	*Picea abies*	Gymnosperm	1960	28354	11
28	*Populus trichocarpa*	Dicot	422.9	41335	28
29	*Prunus persica*	Dicot	227.3	26873	17
30	*Ricinus communis*	Dicot	400	31221	15
31	*Selaginella moellendorffii*	Pteridophyte	212.5	22273	9
32	*Setaria italica*	Monocot	405.7	35471	27
33	*Solanum lycopersicum*	Dicot	900	34727	28
34	*Solanum tubersum*	Dicot	800	35119	21
35	*Sorghum bicolor*	Monocot	697.5	33032	28
36	*Thellungiella halophila*	Dicot	238.5	26351	31
37	*Theobroma cacao*	Dicot	346	29452	17
38	*Vitis venifera*	Dicot	487	26346	17
39	*Volvox carteri*	Lower eukaryote	125.4	14971	6
40	*Zea mays*	Monocot	2500	63540	47

### Nomenclature of CPKs

In the traditional nomenclature system, numbers have been assigned to *CPK* genes according to the serial number in which they are cloned and identified. However, this nomenclature system makes it difficult to determine the exact function of the orthologous counterpart. Plant genomic data are currently increasing daily; therefore, it is necessary to provide a unique and systemic nomenclature to all the *CPK* genes. The development of a new nomenclature system can provide brief functional information regarding the orthologous *CPK* genes in other organisms. Therefore, an orthologous based nomenclature system was used to assign the name to each *CPK* gene of the studied organisms as proposed by different research groups (Supplementary Table 1; Hamel et al., 2006; Kanchiswamy et al., 2013; Mohanta and Mohanta, 2013; Mohanta et al., 2014, 2015a). The *Arabidopsis thaliana* and *Oryza sativa CPK* genes were taken as the orthologous genes for this purpose (Hamel et al., 2006; Schlicker et al., 2006). In this study, the nomenclature system adopted for naming *Arabidopsis*, poplar and rice mitogen-activated protein kinases (MPKs) was extended to other genes (Hamel et al., 2006; Mohanta et al., 2015a). For each *CPK* gene, a name was assigned by taking the first letter of the genus and the first letter of the species, after which *CPK* and the orthology based number of *Arabidopsis thaliana* or *Oryza sativa* were used. In the nomenclature system, the first letter of the genus was kept upper case and the first letter of the species was kept lower case. When the first letter of the genus and species coincided with one another, only the first letter of the genus and the first, second, third or later letter of the species name was considered in conjunction with the suitability of pronunciation. For example, *C. rubella* and *C. reinhardtii* share “C” as first letter of the genus and “r” as the first letter of the species. In this case, *C. rubella CPKs* were named *CrCPK*, while *C. reinhardtii CPKs* were named *CreinCPK*. The monocot specific *CPKs* were named according to *Oryza sativa*, while the rest of *CPKs* were named according to *Arabidopsis thaliana*. This nomenclature system can be useful to provide a unique identity to each *CPK* gene throughout the plant kingdom. The sequence/structural similarities resemble the functional similarities of genes (Aravind et al., 2002; Schlicker et al., 2006; Li et al., 2008). Specifically, the unique orthologous gene of one species may resemble the other gene and undergo similar cellular function. A similar approach is being used to predict potential function of newly sequenced genes and their gene products. It is nearly not possible to study the role of each *CPK* with different functional aspects. Therefore, an orthology based nomenclature system of *CPK* genes will provide basic information regarding its ortholog counterpart (Wright and Bruford, 2006).

### Multiple sequence alignment

The CPK protein sequences of plants were subjected to multiple sequence alignment to identify the conserved domains and motifs. Multiple sequence alignment was carried out using the Multalin software (http://multalin.toulouse.inra.fr/multalin/). The statistical parameters used to carry out multiple sequence alignment were as follows: protein weight matrix, BLOSUM62-12-2; gap penalty at opening, default; gap penalty at extension, default; gap penalty at extremities, none; one iteration only, no; high consensus value, 90% (default); and low consensus value 50% (default).

### Construction of phylogenetic tree

All the protein sequences of CPKs were subjected to multiple sequence alignment in clustal omega to generate a clustal file. The generated clustal file of CPKs was converted to MEGA file format using the MEGA6 software and then used to construct the phylogenetic tree using the same software (Tamura et al., 2013). Different statistical parameters used to construct the phylogenetic tree were; statistical method, maximum likelihood; test of phylogeny, bootstrap method; number of bootstrap replication, 5000; model/method, Jones-Taylor-Thornton; rates among sites, uniform; gaps/missing data treatment, partial deletion; site coverage cutoff, 95%; and branch swap filter, very strong.

### Statistical analysis

Tajima's relative rate test was conducted to understand the rate of evolution of plant CPKs. To accomplish this, MEGA6 software was used to conduct Tajima's relative rate test and Tajima's test of neutrality of the CPKs in MEGA file that used to generate the phylogenetic tree. The statistical parameters used for Tajima's relative rate test were; analysis, Tajima's relative rate test; scope, for three chosen sequences; substitution type, amino acids; gaps/missing data treatment, complete deletion. Tajima's test of neutrality was carried out to understand the polymorphism of *CPK* genes in plants. The statistical parameters used to carry out Tajima's test of neutrality were: analysis, Tajima's neutrality test; scope, all selected taxa; substitution type, amino acids; and gaps/missing data treatment, complete deletion.

Correlation regression analysis (http://www.mathportal.org/calculators/statistics-calculator/correlation-and-regression-calculator.php) and paired *t*-test (http://www.mathportal.org/calculators/statistics-calculator/t-test-calculator.php) was conducted using online tool Math Portal. In both the analysis, genome size was provided in X-axis and CPK gene family size was provided in Y-axis. The paired *t*-test was conducted at significance level of *p* < 0.001.

## RNA isolation and qRT-PCR

Wild type *Brassica rapa* L. sub species oleifera were grown in soil in greenhouse under 16 h light and 8 h dark cycle at 22–25°C for 20 days. Cold treatment was conducted by subjecting the plants to 4°C and drought/heat treatment was applied at 37°C. Treated plant leaves were immediately transferred to liquid nitrogen for further analysis. All experiments were carried out in triplicates. Total RNA was isolated from treated and control samples using Trizol reagent. RNA was treated with RNase free DNAase to ensure that there is no DNA contamination in the RNA sample. First strand cDNA synthesis was carried out using a Fermentas RevertAid first strand cDNA synthesis kit according to the manufacturer's instruction. The primers for the *B. rapa CPK* genes were designed using primer3 software targeting either the extreme 5′ end (extreme 5′ ends are not conserved) or 3′ UTR region which produced an amplicon of 120–200 bp (primer length between 20 and 24 bp) with a melting temperature of 58–60°C (Supplementary Table 2). The primer lengths were kept around 18–22 nucleotides. Quantitative real-time PCR was conducted using a Mx3000P real-time PCR system with SYBR green master mix (2x) (Fermentas) and ROX as the passive reference standard to normalize the SYBR fluorescent signal. PCR amplification was conducted in a 25 μl reaction mixture containing 1 μl cDNA as template, 12.5 μl SYBR green master mix (2x), 1 μl of each forward and reverse primer and nuclease free water up to 25 μl. The thermal profile for quantitative real time PCR reaction was; initial activation at 95°C for 10 min, followed by 40 cycles of 95°C for 30 s, 60°C for 30 s, and 72°C for 30 s. Each quantitative real time PCR was carried out in triplicate on three biological replicates. Primers showing efficiency between 90–105% were considered significant. Relative expression was calculated using *ACT2* as the reference gene. The relative expression of *BrCPK* genes was calculated using the 2^−ΔΔCT^ method (Schmittgen and Livak, 2008).

## Results and discussion

### Larger the genome size is not directly proportional to bigger the gene family

**Figure 1:**
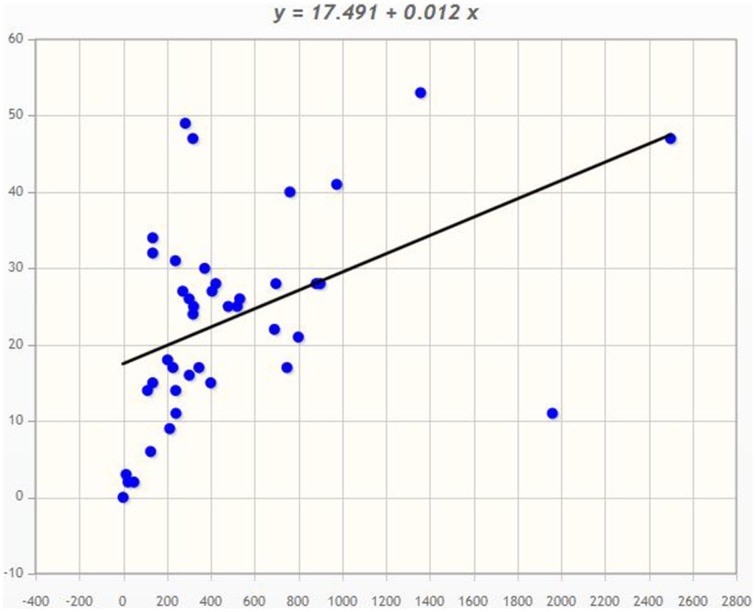
**Correlation regression analysis of Genome size and *CPK* gene family size**. The correlation regression analysis shows, the genome size of an organism is not directly proportional to the size of the gene family. The bigger the genome size isn't directly proportional to the bigger the gene family. In the majority of cases, plant encodes 10–30 *CPKs* per genome. The X-axis denotes the genome size and Y-axis denotes the *CPK* gene family size.

Angiosperms are considered as the most advanced groups of land plants. Although the CPKs of several angiosperms have been widely investigated, such studies have been limited to specific species (Hrabak et al., 2003; Asano et al., 2005; Kanchiswamy et al., 2013; Kong et al., 2013; Zuo et al., 2013; Liu et al., 2014). Therefore, very little information is known about their different genomic aspects. We found that genome size and presence of the *CPK* gene number vary from species to species (Table 1). Among the investigated plants, *Ostreococcus lucimarinus* has the smallest genome (13.2 Mbs), while *Zea mays* has the largest (2500 Mbs). The genome of *Coccomyxa subellipsoidea* encodes the lowest number of *CPK* genes (2), while the genome of *Panicum virgatum* encodes the highest (53) among the studied species. In this study we found that, the larger genome size is not directly proportional to a larger gene family. The *CreinCPK17-5* (*Chlamydomonas reinhardtii*) is the largest *CPK*, containing an ORF of 5940 nucleotides (1979 amino acids), while *CpCPK2* (*Carica papaya*) is the smallest *CPK*, with an ORF length of 693 nucleotides (231 amino acids; Supplementary Table 1). Correlation regression analysis of *CPK* genes compared to the genome size didn't result any linear correlation (Figure 1). Eleven species were found to be clustered within size of 10–20 CPK members and 14 species were found to be clustered with size of 20–30 CPK members (Figure 1). The regression coefficient analysis was found to be *r* = 0.4487. The regression coefficient should be +1/−1 to infer a direct correlation between the genome size and the size of gene family. The paired *t*-test between the genome size and gene family size differs significantly at *p* < 0.001. The *t*-value was found to be 5.9796 with degree of freedom 40 and critical value 3.551. The average size of *CPK* genes of green algae and *Physcomitrella patens* is larger than those of advanced higher plants. The extra sequences of lower eukaryotic plants are probably deleted during evolution from lower eukaryotes to higher eukaryotes owing to the need to adapt to terrestrial habitats (Rensing et al., 2008). These findings indicate that evolution of *CPK* genes occurred via loss of gene size; hence, higher plants do not contain extra sequences. Further genomic analysis revealed that, many *CPK* genes contain either 6, 7, or 8 introns with a maximum of 11 introns in at least one gene, except for *Carica papaya, Ostreococcus lucimarinus*, and *Ricinus communis* (Supplementary Table 1). No introns were detected in some of the *CPK* genes of *O. lucimarinus* and *P. patens*. The *O. lucimarinus* is a lower aquatic algae and *P. patens* is the first land plant, explaining their conserved and ancient origin (Palenik et al., 2007; Jain et al., 2008; Rensing et al., 2008). The molecular masses of CPKs were reportedly vary from 40 kD to 90 kD (Martín and Busconi, 2001; Tuteja and Mahajan, 2007); however, we found that, they ranges from 26.134 kD (CpCPK2) to 198.916 kD (CreinCPK17-5). The isoelectric point of CPKs ranges from 4.61 (PaCPK33, OlCPK3) to 9.33 (AtCPK16) (Supplementary Table 3). The analysis of amino acid composition of CPKs showed that, leucine (L) was present most frequently (8.46 times per CPK) (Supplementary Table 4), while tryptophan (W) was present least frequently (0.91 times per CPK). Aspartic acid (D) and glutamic acid (E), which were responsible for calcium sensing, were found to be 7.14 and 7.83 times per CPK, respectively (Supplementary Table 4).

These finding indicates that, when more energy is required to synthesize a particular amino acid, there is less chance of that amino acid occurring more frequently (Akashi and Gojobori, 2002). The average abundance of T in CPK is only 0.91 times per CPK, and the energy required to synthesize W is 74.3. High energy input is required to synthesize W; therefore, the CPK protein can only incorporate 0.91 W per CPK. The energy required to synthesize D and E is 12.7 and 15.3 unit, respectively (Akashi and Gojobori, 2002); accordingly, the plants can able to accommodate 7.14 D and 7.83 E per CPK.

### CPKs contains four conserved D-x-D motifs in EF-hand domain

**Figure 2:**
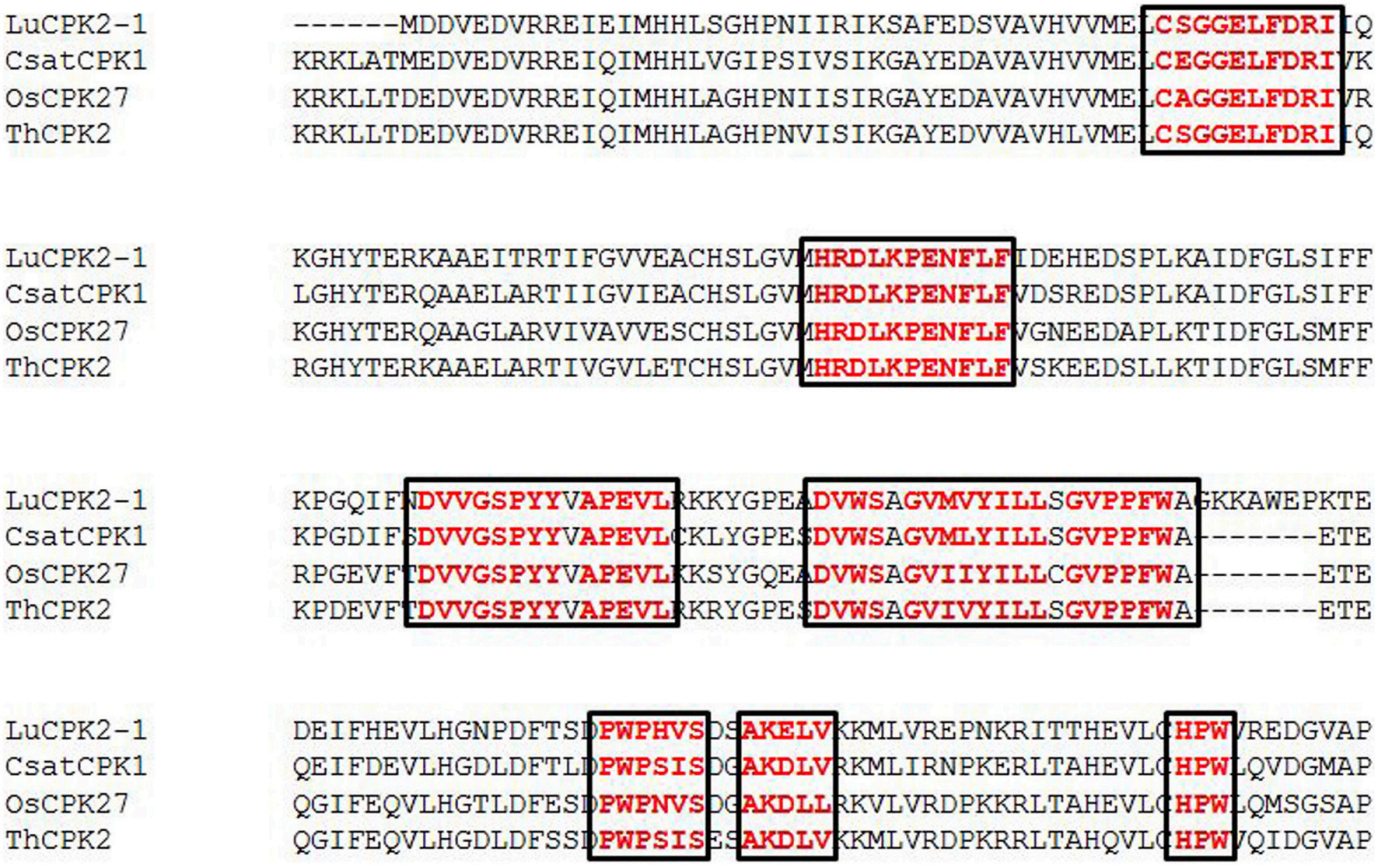
**Presence of conserved domains in the kinase domain of plant calcium-dependent protein kinases (CPKs) (red)**. The major conserved domains are C-S-G-G-E-L-F-D-R-I, H-R-D-L-K-P-E-N-F-L-F, D-V-V-G-S-P-Y-Y, A-P-E-V-L, D-V-W-S, G-V-M-V-Y-I-L-L, G-V-P-P-F-W, P-W-P-H-V-S, A-K-E-L-V, and H-P-W. The complete details describing the conserved domains of CPKs are provided in the Supplementary Figures.

Even though many studies have been carried out, no specific conserved domain/motif has been reported for kinase domains, auto-inhibitory domain and EF-hand domains of the CPK protein. We believe, this is the first report showing the presence of conserved domains/motifs in CPKs. To identify the conserved domains/motifs, multiple sequence alignments of monocot, dicot and lower eukaryotic plants were carried out separately. We found that, monocot and dicot plants shared some common conserved sequences in the kinase domains, C-x-G-G-E-L-x-D-R-I, H-R-D-L-K-P-E-N-F-L, D-x-V-G-S-x-Y-Y, A-P-E-V-L, D-V/I-W-S, G-V-I-x-Y-I-L-L, G-x-P-P-F-W, P-W-P-x-I-S, A-K-D-L-V, and H-P-W (Figure 2, Table 2, Supplementary Table 5, Supplementary Figures 1–3), while the kinase domain of lower eukaryotic plants such as *C. reinhardtii, V. carteri, M. pusila, O. lucimarinus*, and *P. patens* contains little bit different conserved sequences, M-E-L-C-x-G-G-E-L-F, H-R-D-L-K-P-E-N-F-L, D-F-G-L-S-V/x, A-P-E-V-L/x, D-I-W-S-x-G-V, and P-F-W (Supplementary Table 5, Supplementary Figure 4).

**Table 2:** **Presence of conserved motifs in EF-hands of CPKs**.

**Conserved motifs in EF-hands**			
**Monocot**	**Dicot**	**Lower eukaryotes**	**Altogether**
E-E-I/x	E-E-I/x		E-E-I/x
E-M-F	E-M-F		
D-x-D	D-x-D		D-x-D
	G-x-I		G-x-I
D-E-L	D/E-E-L		D-E-L
	D/E-x-E		D-E-x-E
D-x-D	D-x-D	D-x-D	D-x-D
D-x-x-E-F	D-Y-x-E-F	D-x-x-E	E-F-I-x
E-D-x_(4)_-A-F			
F-D-x-D	F-D-x-D		D-x-D
			G-x-I
	G-I-x-Y		
E-E-L	D-E-L		D-E-L
D-x-D	D-x-D	D-x-D	E-D-x-D
D-G-x-I	D-G-R/x-I		D-x-D/G-x-I
Y-x-E-F-x-x-M-M	Y-x-E-F-x-x-M-M		Y-x-E-F-x-x-M-M

*Monocot and dicot plants share common E-E-I/x, E-M-F, and D-x-D motifs in EF-hands. Higher plants contain 4 D-x-D motifs in their EF-hands, while lower eukaryotes have only two D-x-D motifs*.

**Figure 3:**
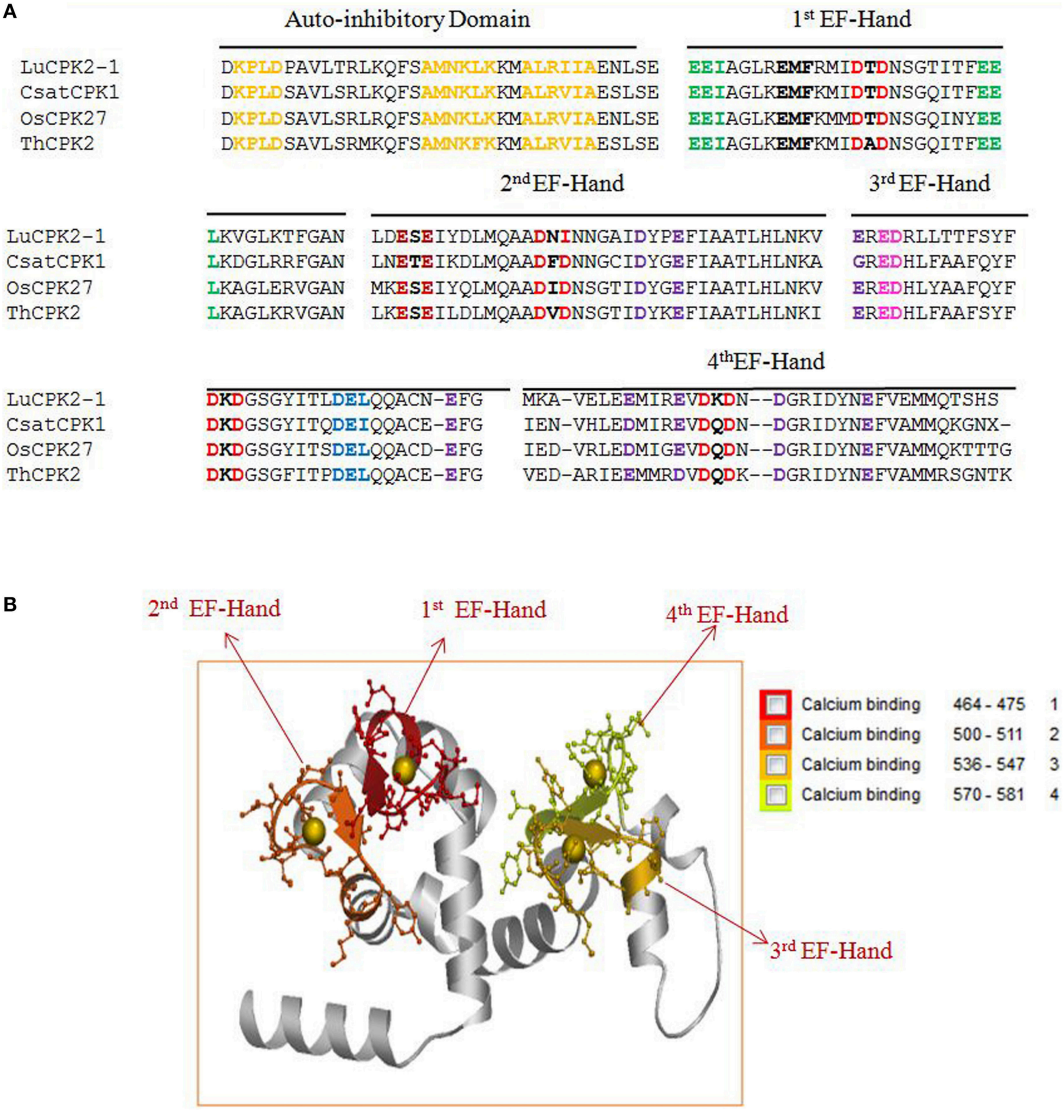
**(A)** The presence of conserved domains and motifs in auto-inhibitory and EF-hand regions of calcium-dependent protein kinases (CPKs) in plants. The auto-inhibitory domain contains K-P-L-D, A-M-N-K-L-K, and A-L-R-I-I-A conserved sequences (orange). The EF-hand regions of the regulatory domain of CPK have D-x-D (red) and D/E-E-L motifs (light blue). Higher plants contain four D-X-D motifs in their EF-hands, while lower eukaryotes have only two D-x-D and one D-x-x-E motif. Other motifs found in the EF-hands include E-E-I and E-E-L (green). The presence of the conserved motif E-E-I/x (green) at position 1, 2, and 3, as well as E-M-F at position 8, 9, and 10 (bold black) are first EF-hand specific. The E-x-E motif (dark red) at position 3, 4, and 5 is second EF-hand specific, while the x-E-D motif at position 3 and 4 (pink) is third EF-hand specific. The presence of conserved D/E at position 11 and 12 is fourth EF-hand specific. Some conserved D and E residues present in the EF-hand regions of plants are indicated in purple. Complete details regarding the conserved domains of CPKs can be found in the Supplementary Figures. **(B)** Three dimensional structure of the EF-hand domain of plant CPK (AtCPK1). The structure was created using the Swiss-Model workspace (http://swissmodel.expasy.org) for AtCPK1 as a representative sequence. The colored boxes in the bottom represent the presence of calcium binding motifs within the specific region of EF-hands. Regions ranging from amino acids 464–475, 500–511, 536–547, and 570–581 belong to the first, second, third and fourth EF-hands respectively. All calcium binding EF-hands start with the D-x-D motif. The starting regions of all calcium-binding EF-hands contain conserved D-x-D motif and ends with conserved glutamic acid (E). The conserved D-x-D motif present at position 14, 15, and 16 and conserved E residue is present at position 25 in each EF-hand. Therefore, amino acids 14–25 of each EF-hand are assumed to play crucial roles in calcium binding activity. In conclusion, EF-hands contain conserved aspartic acid (D) and glutamic acid (E) residues, which may be critical to accessory signaling in calcium binding.

The conserved sequences D-x-V-G-S-x-Y-Y, G-V-I-x-Y-I-L-L, G-x-P-P-F-W, P-W-P-x-I-S, A-K-D-L-V, and H-P-W present in higher plants are absent from the kinase domain of lower eukaryotic plants (Supplementary Table 5). Earlier it was thought that, auto-inhibitory domain does not have any conserved sequence. But, we found that, the auto-inhibitory domains of monocot, dicot and lower eukaryotic plants are also highly conserved in nature. The common conserved domains present in the auto-inhibitory domains of monocot and dicot plants are K-P-L-D, F-S-A-M-N-K-L, and A-L-x-x-I-A (Figure 3A, Supplementary Table 6). The auto-inhibitory domain of lower eukaryotic plants doesn't contain K-P-L-D and A-L-x-x-I-A domains and only contains the A-M-N-K-L domain (Supplementary Table 6).

**Figure 4:**
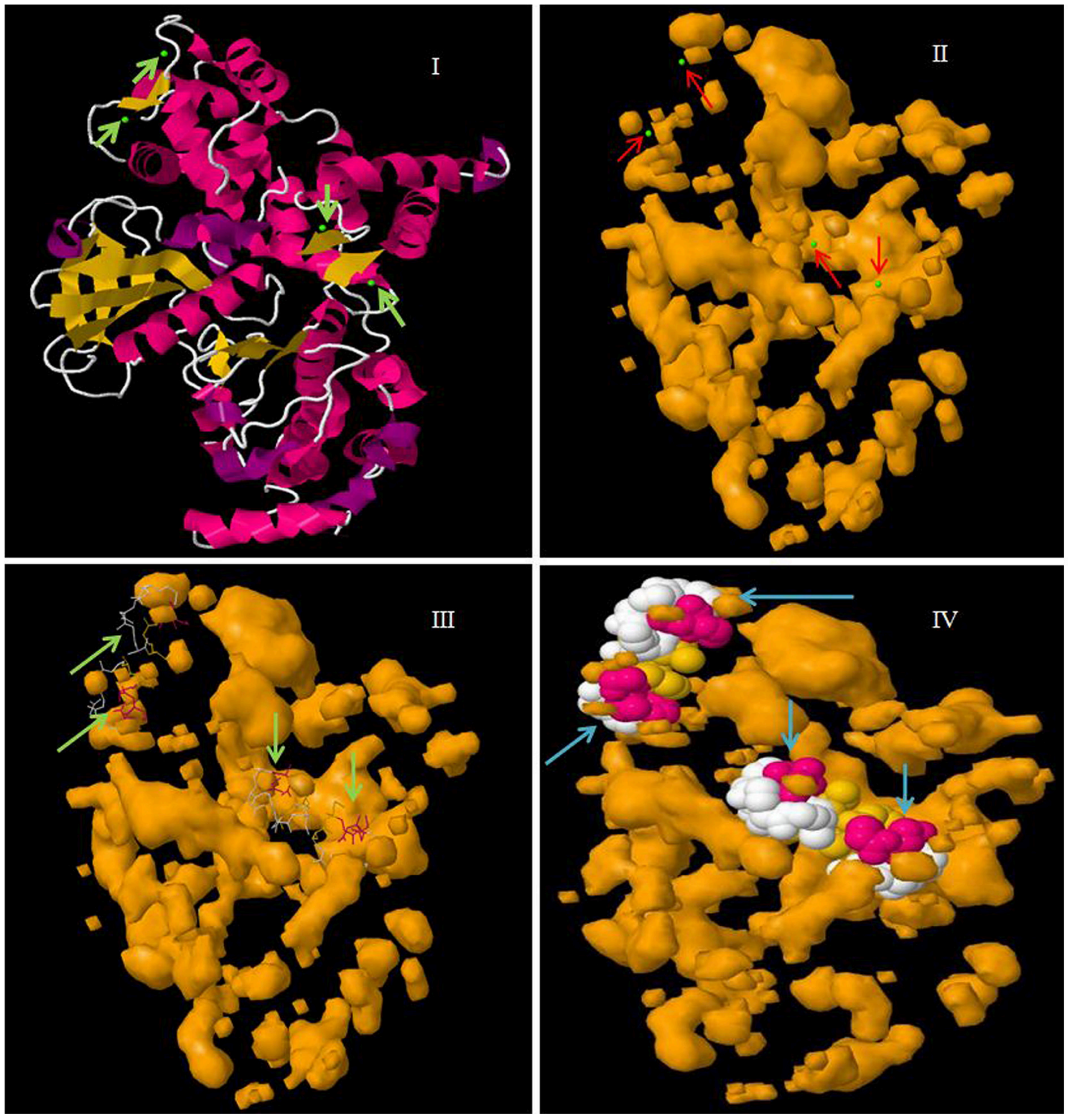
**Molecular structure of CPK protein showing four EF-hand domains**. In the figure, **(I)** represents the secondary structure of CPK protein with calcium binding sites in green, **(II)** the cavity surface of CPK protein with calcium binding sites shown in green, **(III)** the Ca^2+^ ligand interaction with CPK protein, and **(IV)** a space fill view of the Ca^2+^ ligand interaction in cavity model of CPK protein. The molecular model was constructed in the Geno3D server using protein sequence of AtCPK1 as the query search.

The EF-hands of CPK bind the calcium ions and play a significant role in gene regulation (Valmonte et al., 2014). The Ca^2+^ion binds to either D or E amino acid of the EF-hand which leads the auto-inhibitory domain being released from the kinase domain, and subsequent gene regulation (Hwang et al., 2000; Weljie et al., 2003; Tuteja and Mahajan, 2007; Dadacz-Narloch et al., 2011; Boudsocq and Sheen, 2013). Therefore, the presence of D or E amino acid in the EF-hand is very crucial. The EF-hand containing the regulatory domains of CPKs is also highly conserved in nature. The monocot and dicot plants share some common motifs in their EF-hand domain which are; E-E-I/x, D-x-D, D/E-E-L, D-Y-x-E-F, F-D-x-D, E-E-L, D-G-x-I, and Y-x-E-F-x-x-M-M (Figure 3A, Table 2, Supplementary Table 5). Additionally, one extra domain E-D-x_(4)_-A-F is conserved in monocot plants which is absent in dicot plants. The lower eukaryotic plants possess only one D-x-D and D-x-x-E motif in their EF-hand domain. The monocot and dicot (higher eukaryotes) plants possess at least four D-x-D (one D-x-D motif in each EF-hand) and two D/E-E-L motifs in their EF-hands, while lower eukaryotes contain only two D-x-D and one D-x-x-E motifs in their EF-hands (Supplementary Table 5). The two D-x-D motifs of lower eukaryotic plants are present only in the third and fourth EF-hands. Instead of presence of D-x-D and D/E-E-L motifs in CPKs, they also found to contain some substitute motifs like x-D/E-L, Q-E-L (BdCPK30, PvCPK30-1, PvCPK30-2, SiCPK30, SbCPK30, and OsCPK9) and E-E-F/M (BdCPK7-2, PvCPK8-2, SiCPK7-1, SbCPK8-1, ZmCPK8-1, ZmCPK32-1, OsCPK8, PvCPK7-1, BdCPK16-2, OsCPK3, OsCPK4, SiCPK16, SbCPK28, ZmCPK16-1/2, and PvCPK16-2) (Supplementary Table 7). Earlier, Cheng et al. (2002) reported that EF-hands are most conserved at position 1 and 2, while they are least conserved at position 4 (Cheng et al., 2002). In this study, we found that, in the first EF-hand of higher eukaryotic plants (monocot and dicot), the E-E-I/x motif is conserved at position 1, 2, and 3 (Figure 3A). However, all the four EF-hands contain the D-x-D motif at position 14, 15, and 16 and the D/E-E-L motif is at position 24, 25, and 26 respectively (Figures 3A,B, 4). The first EF-hand contains an E-M-F motif at position 8, 9, and 10. In the second EF-hand, there is an E-x-E motif at position 3, 4, and 5, while in the third EF-hand; the x-E-D motif is present at position 3 and 4. In the fourth EF-hand, there is no conserved amino acid at position 1, 2, or 3 but there is presence of a conserved D/E residue at position 11 and 12. According to our findings, the conserved domains/motifs at position14, 15, 16, 24, 25, and 26 are crucial for all EF-hands that are responsible for Ca^2+^ binding. The specific conserved motif E-E-I/x at position 1, 2, and 3 and E-M-F at position 8, 9, and 10 are first EF-hand specific (Figure 3A). The E-x-E motif at position 3, 4, and 5 is second EF-hand specific, while the x-E-D motif at position 3 and 4 is third EF-hand specific (Figure 3A). The presence of conserved D/E amino acids at position11 and 12 is fourth EF-hand specific. The presence of the conserved of CPK motifs from lower to higher plants explains the homologous nature of gene evolution (Thornton and DeSalle, 2000). All EF-hands shows the differences in their first, second, third and fourth amino acid positions. When compared to higher eukaryotes, lower eukaryotes possess only two D-x-D motifs at the second and third EF-hand (Supplementary Table 5). These D-x-D motifs are also present in position 14, 15, and 16 of the second and third EF-hands in lower eukaryotic plants. Some of the lower eukaryotes contain an E-E-I/x motif at the first EF-hand. In the first and fourth EF-hands of lower eukaryotes, D is conserved at position 14, while in the second EF-hand, D and E are conserved at position 22 and 25, respectively. These differences in numbers and positions of EF-hands are likely cause variations in the allosteric properties of the Ca^2+^ binding and activation threshold (Hrabak et al., 2003). The number of EF-hand is very important to determine the Ca^2+^ regulation in CPK activity. Site directed mutagenesis of conserved E residues in each EF-hand showed that, a closer proximity of the EF-hand to the auto-inhibitory domain shows an effect on Ca^2+^ regulation (Zhao et al., 1994).

### CPKs contains membrane targeting palmitoylation and myristoylation sites

Very little information is available regarding the N-terminal variable domain of CPK. It has been reported that, the N-terminal variable region contains sub-cellular targeting information (Harmon et al., 2000; Curran et al., 2011). However, none of the 34 *Arabidopsis* CPKs are predicted to be integral membrane proteins (Cheng et al., 2002). Despite the variability at the N-terminal domain, most CPKs have a G residue at the second position and a C residue at 3, 4, 5, and 6 positions. This N-terminal G residue undergoes modification by myristic acid which is known as myristoylation (Smotrys and Linder, 2004). The N-myristoylation has been reported to promote protein-membrane attachment and protein-protein interactions (Cheng et al., 2002). The mutation of the N-terminal G residue abolishes lipid modification and prevents membrane association (Martín and Busconi, 2001). Among the 950 CPKs, 844 have the G residue at the second position (Table 3). In *Arabidopsis* it has reported that, the N-terminal G residue of AtCPK2 is myristoylated, and the first ten amino acids of CPKs are known to be critical for localization to the endoplasmic reticulum (ER) membrane (Lu and Hrabak, 2002). The second lipid modification leads to addition of palmitate to the C residue at position 3, 4, or 5 of the N-terminal region of CPKs (Cheng et al., 2002). Some major conserved palmitoylation motifs of CPKs found during this study were M-G-C, M-G-N-C, M-G-N-C-C, M-G-N-T-C-V, and M-G-N-C-C-R (Table 3). Both myristoylation at the N-terminal G residue and palmitoylation at the N-terminal C residues at position 4 and 5 have been validated experimentally in membrane bound OsCPK2 (Martín and Busconi, 2001). When myristoylation of OsCPK2 was abolished by mutating N-terminal G amino acid, the protein could no longer be palmitoylated, indicating that, myristoylation might be the prerequisite to palmitoylation (Martín and Busconi, 2001).

**Table 3:** **Protein myristoylation and palmitoylation are important post-translational modification events required for sub-cellular targeting of CPKs**.

**SI No**.	**Consensus myristoylation and palmitoylationsite sequence**	**No. of CPK genes**
1	MGNC	268
2	MGNC**C**	201
3	MGC	60
4	MGNTCV	57
5	QFGTTYLC	45
6	MGNC**C**R	40
7	MGLC	36
8	MGGC	35
9	MGNNC	26
10	MGSC	26
11	MGNSC	21
12	QFGTTFLC	20
13	MGIC	17
14	MGNCNAC	16
15	MGQC	15
16	MGNAC	14
17	QFGTTYQC	13
18	MGNVC	12
19	MGVC	12
20	MGNQC	8
21	QFGVTYLC	5
22	QFGITYLC	5
23	VHLVMELC	4
24	MGNCNTC	4
25	MELC	4

*Protein myristoylation is usually carried out in glycine (G) residues and is conserved at the second position (green). Similarly, protein palmitoylation is carried out in cysteine (C) residues (red). The 25 likely conserved consensus sequences of CPK palmitoylation sites found in this study are listed. The presence of C amino acid at position3, 4, and 5 plays a crucial role in CPK palmitoylation*.

### Phylogenetic analysis shows CPKs are grouped into four groups

**Figure 5:**
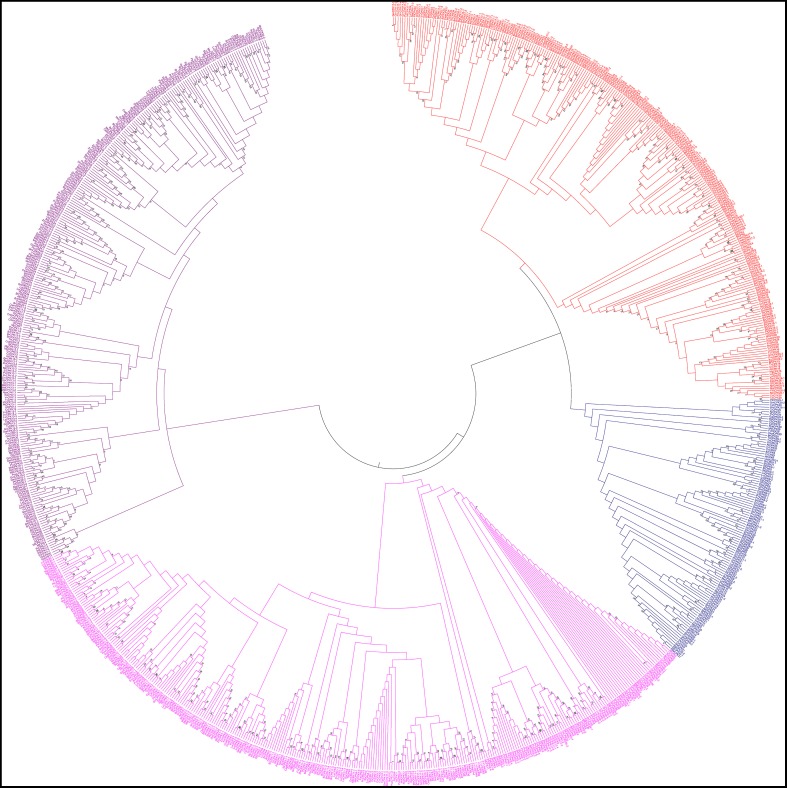
**Phylogenetic tree of calcium-dependent protein kinases (CPKs)**. The CPKs of monocots, dicots, green algae, bryophytes, pteridophytes, and gymnosperms were searched from different genome databases using *CPK* genes of *Arabidopsis thaliana* as queries. Identified sequences were only accepted if the corresponding proteins had serine/threonine protein kinase domain and four calcium binding EF-hand domains. A phylogenetic tree was constructed using Molecular Evolutionary Genetics Analysis 6 (MEGA 6) by neighbor joining method with 1000 bootstrap replicates. The constructed phylogenetic tree showed that, CPKs are clustered into four different groups: A (red), B (blue), C (fuschia), and D (purple).

In this study, we constructed an unrooted phylogenetic tree of CPKs from monocot, dicot and lower eukaryotic plants (Figure 5). The result revealed that *CPK* genes fell into four different groups; group A (red), B (blue), C (fuschia), and D (purple) (Figure 5). Previous studies by different researchers also reported the presence of four groups in CPKs (Lu and Hrabak, 2002; Hamel et al., 2014). The phylogenetic tree showed that, the basal architecture of the *CPK* gene family of three classes (monocot, dicot and lower eukaryotic plants) is conserved and emerged from a common ancestor. Significant sequence similarities exist among the CPKs, indicating that they are probably arose very recently via gene duplication with similar or overlapping functions. The orthologous genes are considered as evolutionary counter parts that must be derived from a common ancestor, while paralogs are homologous genes that evolved through duplication within the same genome (Chen et al., 2005). Paralogs tend to evolve due to new functions for better adaptation (Makarova et al., 2005; Wu et al., 2006). When duplication precedes speciation, each of the paralogs gives rise to a distinct line of orthologous descent (Vleeshouwers et al., 2001; Ting et al., 2004; Chauve et al., 2008). Conversely, when duplication occurs after a particular speciation event in one or both lineages independently, one-to-one orthologous relationships cannot be delineated in principle, resulting in co-orthologs (Li et al., 2010; Mahmood et al., 2012; Yan et al., 2012). The lineage specific expansion of paralogous gene families, which in some cases accounts for a sizable fraction of the genome, is considered as one of the major mechanisms of adaptation (Jordan et al., 2001; Lespinet et al., 2002).

### CPKs are undergone vivid gene duplication during the course of evolution

The *CPK* gene family is characterized by the presence of several paralogs that share a significant level of homology. These closely related *CPK* genes most likely emerged following a recent gene duplication event, which would explain why *CPK* genes have not yet diversified much. In some cases, duplication may be due to diversification events or lineage specific. Gene duplication may increase due to auto-polyploidization, which is the most common mechanism of gene duplication in plants.

The genomes of *C. subellipsoidea* and *M. pusila CCMP1545* contain only two *CPK* genes, whereas *O. lucimarinus* contains only three *CPK* genes, indicating no significant duplication of *CPK* genes in these species. Another lower eukaryotic alga, *C. reinhardtii*, contains 14 *CPK* genes with several paralogs; while *V. carteri* has six *CPK* with no paralogous genes, indicating that, gene duplication may have started from *Chlamydomonas*. The first land plant, *P. patens*, contains 25 *CPK* genes in its genome, including several paralogous genes; however, the pteridophyte *S. moellendoffii* contains only nine *CPK* genes (Banks et al., 2011; Baker et al., 2013). Although *Picea abies* has undergone vivid duplication (Morse et al., 2009; Mackay et al., 2012; Nystedt et al., 2013), we found that the model gymnosperm *P. abies* contains only 11 *CPK* with no paralogous genes. Only the genome of *P. patens* in bryophytes has been sequenced and confirmed to have undergone recent large scale duplication (Bisova et al., 2005; Zimmer et al., 2008). Nevertheless, it is difficult to predict whether recent duplication events contributed to expansion of the *CPK* family of other non-vascular plants.

### CPKs are evolved by balancing selection

Statistical analysis is very important to understand the significance of the study. Therefore, we used Tajima's relative rate test to analyze three samples (sequences) selected at random (Tajima, 1998). We repeated this analysis three times using different sequences. For all three cases, the *p*-value and *X*^2^- (Chi-square) test were found to be significant (Table 4).

**Table 4:** **Results of Tajima's test for 3 sequences in three replicate analyses**.

**Configuration**	**AtCPK18 PpCPK16-3 SmCPK16**	**ThCPK12 OsCPK3 CreinCPK17-6**	**GrCPK11-2 OsCPK3 CreinCPK17-6**
Identical sites in all three sequences	307	121	122
Divergent sites in all three sequences	49	164	169
Unique differences in sequence A	66	23	22
Unique differences in sequence B	33	57	54
Unique differences in sequence C	29	75	73
*P*-value	0.00091	0.00014	0.00024
*X*^2^-test	11.00	14.45	13.47
Degree of freedom	1	1	1

*A P-value less than 0.05 is often used to reject the null hypothesis of equal rates between lineages. The analysis involved three amino acid sequences. All positions containing gaps and missing data were eliminated*.

Tajima's neutrality test (*D*-test) was conducted to understand the polymorphism of CPKs (Tajima, 1989). We found that, Tajima's *D*-value for all CPKs is 5.269218 (Table 5). A negative Tajima's D signifies very low frequency of polymorphism relative to the expectation indicating expansion of population size by purifying selection. Similarly, a positive Tajima's D indicates a high level of polymorphism (Tajima, 1989; Fu and Li, 1993; Simonsen et al., 1995). The high level of polymorphism indicates decrease in population size by balancing selection. In the present study, the Tajima's *D*-value for all CPKs was 5.269218 (Table 5) which indicates that, plant CPK genes are undergoing balancing selection and hence decreasing in population size. A Tajima's D of zero (*D* = 0) indicates that, the average heterozygosity is equal to the number of segregating sites. This can be inferred as an observed variation similar to the expected variation, indicating that the population evolved in mutation-drift equilibrium. A Tajima's (*D* < 0) indicates greater lower average heterozygosity than the number of segregating sites and that there will be very rare alleles at low frequency. A Tajima's *D*-value (*D* > 0) indicates greater average heterozygosity than the number of segregating sites and multiple alleles presents at high frequency. This leads to balancing selection by sudden population contraction. A *D*-value greater than +2 or less than −2 is considered as significant; accordingly, the *D*-value in the present study is +5.269218 which is significant (Tajima, 1989; Fu and Li, 1993; Simonsen et al., 1995).

**Table 5:** **Results of Tajima's Neutrality Test**.

***m***	***S***	***p*_s_**	**Θ**	**π**	***D***
950	24	1.000000	0.134532	0.427892	5.269218

*The analysis involved 950 amino acid sequences. All positions containing gaps and missing data were eliminated. There were a total of 24 positions in the final dataset. Evolutionary analyses were conducted in MEGA6*.

*m, number of sequences; n, total number of sites; S, number of segregating sites; p_s_, S/n; Θ, p_s_/a_1_; π, nucleotide diversity, and D is the Tajima test statistic*.

### CPK genes are differentially expressed during stress conditions

**Figure 6:**
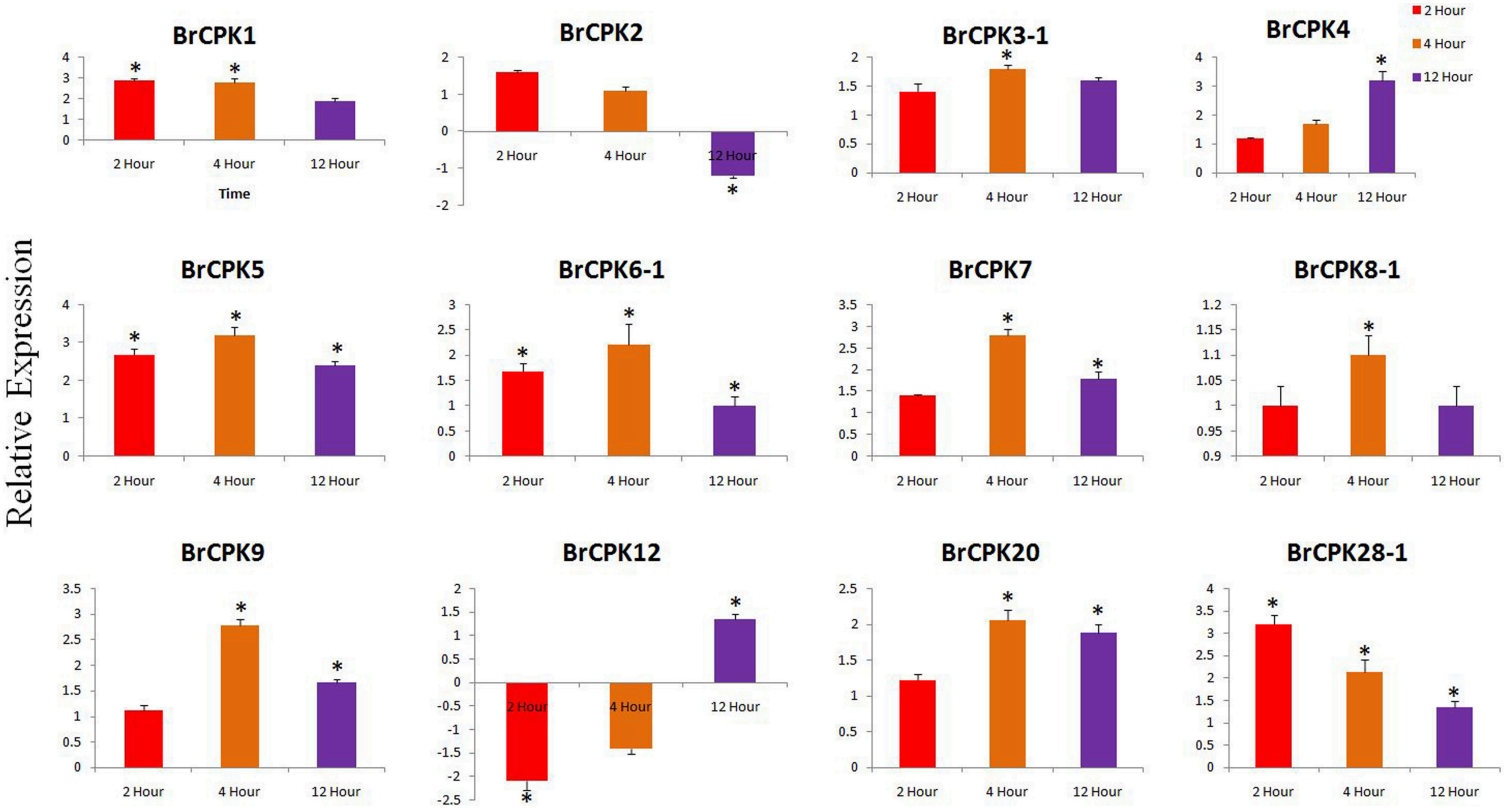
**Quantitative real time PCR of cold treated *B. rapa CPK* genes at 2, 4, and 12 h**. The transcript levels of *BrCPKs* were normalized with *ACT2* and expressed as fold change relative to normalized transcript level in leaves of control (untreated plants at respective time points) plants. The metric bar represents the standard errors (SE) and star indicates statistical significance at ^**^p < 0.05.

The expression analysis of some *B. rapa CPK* genes treated with cold and heat/drought stress were analyzed. In cold treated *B. rapa*, expression of *BrCPK1* was up-regulated at 2, 4, and 12 h (Figure 6). Additionally, *BrCPK2* was up-regulated at 2 and 4 h and got down regulated at 12 h. The *BrCPK4* was gradually up-regulated from 2 to 12 h (Figure 6). The expression of *BrCPK5* was up-regulated by more than 2-fold in all three time periods. This indicates that, *BrCPK1, BrCPK2, BrCPK4*, and *BrCPK5* are cold stress responsive genes. The up-regulation at 2 and 4 h time point indicates the early response of calcium signaling genes to cold stress. Down regulation of *BrCPK2* at 12 h indicates, this gene plays significant role in early stage of calcium signaling event and gradually its expression decreases at late stage. The *BrCPK12* was down regulated at 2 and 4 h and up-regulated at 12 h; while *BrCPK28-1* up-regulated at 2 h and then gradually down regulatedat 12 h (Figure 6). We found that, the maximum number of *CPK* genes were up-regulated in response to cold stress indicating that these genes play significant roles in cold stress response in *B. rapa*.

**Figure 7:**
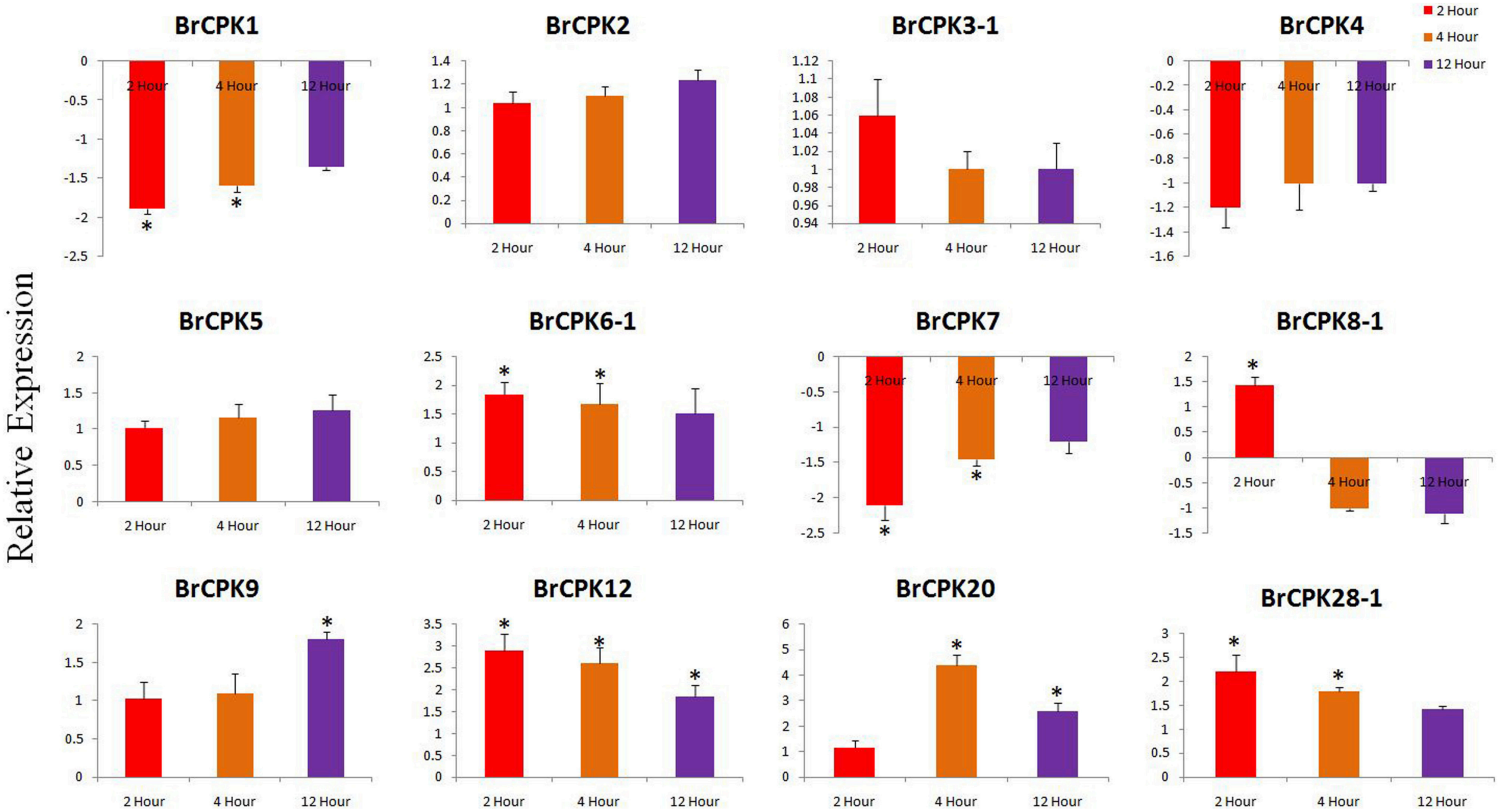
**Quantitative real time PCR of drought treated *B. rapa CPK* genes at 2, 4, and 12 h**. The transcript levels were normalized with EF1 and *ACT2* and expressed as fold change relative to normalized transcript levels in leaves of control (untreated plats in respective time point) plants. The metric bar represents the standard errors (SE) and star indicates statistical significance at ^*^p < 0.05.

Expression analysis of *B. rapa CPK* genes subjected to drought stress revealed dynamic responses (Figure 7). The *BrCPK1* down regulated in all three time periods similar to *BrCPK4* and *BrCPK7* (Figure 7). However, *BrCPK8-1* was up-regulated at 2 h, then gradually down regulated. *BrCPK12* was up-regulated at all the three time points. Similarly, the maximum expression of *BrCPK20* was observed at 4 h, when it was up-regulated by more than 4-fold relative to control. The *BrCPK1* and *BrCPK4* was up-regulated in cold stress and down regulated in drought stress. These result indicates, *BrCPKs* responds to cold and drought stress differently. Early studies show that, *CPKs* are significantly modulated by cold, drought and salt stresses. The *OsCPK4* was found to be induce under cold and drought stresses (Ray et al., 2007). The *BrCPK4* significantly up-regulated at 4 and 12 h time point similar to *OsCPK4*. Rice seedlings grown under cold stress for 3 h shows induction of *OsCPK4, OsCPK12, OsCPK15*, and *OsCPK21* (Ray et al., 2007). The *BrCPK12* found to be down regulated at 2 and 4 h time point and later get up-regulated at 12 h time point. This result shows that, *BrCPK4* and *BrCPK12* are responsive to cold stress in time dependent manner. *BrCPK7* shows up-regulation due to cold stress. The over expression line of *OsCPK7* shows enhanced tolerance to cold stress (Saijo et al., 2000). This shows that, *BrCPK7* also responds to cold stress and it's over expression might responsible for cold tolerance. Unlike cold stresses, *CPKs* are also highly modulated due to drought stresses too (Figure 7). The *AtCPK3* and *AtCPK6* regulate ABA mediated stomatal closure (Mori et al., 2006). Thus, up-regulation of *BrCPK3-1* and *BrCPK6-1* in drought stress might be directly linked to stomatal closure in ABA mediated manner. *AtCPK6* over expression plant shows enhanced tolerant to drought stress (Xu et al., 2010). So, up-regulation of *BrCPK6* in drought stress directly reflects its role in drought stress response.

## Conclusion and future perspectives

The *CPKs* constitute a large multi-gene family in various plant species that play important roles in various physiological processes, including plant growth and development, as well as abiotic and biotic stress responses. *ThCPKs* also plays considerable roles in stress tolerance. Extensive efforts using integrative approaches have provided conclusive evidence that *CPKs* are versatile and evolutionarily conserved genes that transduce Ca^2+^ signals as sensors. The *CPKs* are involved in a sophisticated Ca^2+^ signaling network via protein phosphorylation and coordinate dynamic cellular processes for plant growth and development. Further studies will lead to a better understanding of specific and redundant roles of *CPKs* and their conserved domains in different signaling networks. To fully elucidate the Ca^2+^-mediated signaling network, it is essential to establish cellular, molecular and genetic linkage with Ca^2+^ channels, transporters and pumps for spatio-temporal analysis of *CPK* gene activation and translocation. *In vivo* activation of *CPK* genes has been limited because of difficulties in maintaining required Ca^2+^ levels, which reflect the physiological state in cell extracts. Raising specific anti-phosphopeptide antibodies against CPK protein or their substrates would facilitate monitoring of CPK activation, while identification of downstream substrates and unique regulatory features of each isoform will provide insight into CPK signaling, enabling understanding of their integrated roles in diverse biological process.

## Author contributions

TM conceived the idea, design the experiment analyzed data and drafted the manuscript. NM was analyzed the data. YM and HB revised the manuscript.

### Conflict of interest statement

The authors declare that the research was conducted in the absence of any commercial or financial relationships that could be construed as a potential conflict of interest.
