# Supplementary material for: Genome-Wide Identification of Calcium Dependent Protein Kinase Gene Family in Plant Lineage Shows Presence of Novel D-x-D and D-E-L Motifs in EF-Hand Domain
Source: Front Plant Sci. 2015 Dec 24;6:1146. doi: 10.3389/fpls.2015.01146 (PMC4690006; doi:10.3389/fpls.2015.01146)
Supplement: Supplementary file 1 [file Table1.PDF]

Supplementary Table 1

Gene name, locus ID and complete genomic information of different CPK genes of different plant species.

Gene Name	Locus ID	CDS	No. of a.a	No. of introns	5’-3’ Coordinate
Aquilegia coerulea					
AcCPK2	Aquca_009_00629	1734	577	6	scaffold_9: 3739849 - 3744162
AcCPK3	Aquca_013_00210	1602	533	7	scaffold_13: 1343494 - 1347565
AcCPK7-1	Aquca_014_00772	1608	535	7	scaffold_14: 4558862 - 4564244
AcCPK7-2	Aquca_009_00303	1596	531	7	scaffold_9: 1727577 - 1731638
AcCPK11	Aquca_014_00140	1569	522	7	scaffold_14: 812449 - 816001
AcCPK13	Aquca_004_00375	2231	541	6	scaffold_4: 5346666 - 5353986
AcCPK20-1	Aquca_004_00556	1839	612	6	scaffold_4: 7115223 - 7122796
AcCPK20-2	Aquca_009_00644	2919	972	7	scaffold_9: 3830142 - 3835795
AcCPK26-1	Aquca_015_00273	1677	558	8	scaffold_15: 2341003 - 2347846
AcCPK26-2	Aquca_007_00944	1737	578	8	scaffold_7: 7155463 - 7161579
AcCPK28-1	Aquca_005_00352	1722	573	11	scaffold_5: 3593873 - 3607125
AcCPK28-2	Aquca_007_00052	1614	537	11	scaffold_7: 500593 - 507791
AcCPK29	Aquca_111_00023	1581	526	7	scaffold_111: 280443 - 284578
AcCPK30	Aquca_002_00453	1647	548	6	scaffold_2: 3860293 - 3865499
AcCPK33	Aquca_005_00072	2340	542	7	scaffold_5: 739914 - 746198
AcCPK34	Aquca_004_00793	1593	530	7	scaffold_4: 9274882 - 9278310
Arabidopsis thaliana					
AtCPK1	At5g04870	1833	610	6	1416783-1420338
AtCPK2	At3g10660	1941	646	6	3331398-3334268
AtCPK3	At4g23650	1590	529	8	12324758-12327459
AtCPK4	At4g09570	1506	501	6	6049517-6052335
AtCPK5	At4g35310	1671	556	7	16801987-16804995
AtCPK6	At2g17290	1635	544	7	7516415-7519633
AtCPK7	At5g12480	1608	535	8	4047515-4050533
AtCPK8	At5g19450	1602	533	7	6558426-6561534
AtCPK9	At3g20410	1626	541	7	7116201-7119121
AtCPK10	At1g18890	1638	545	6	6522764-6525962
AtCPK11	At1g35670	1488	495	6	13205381-13208252
AtCPK12	At5g23580	1473	490	5	7950202-7952532
AtCPK13	At3g51850	1587	528	6	19232467-19235889
AtCPK14	At2g41860	2443	530	6	17467344-17469786

AtCPK15	At4g21940	1686	561	7	11640802-11643762
AtCPK16	At2g17890	1716	571	11	7769885-7772627
AtCPK17	At5g12180	1587	528	6	3937024-3939596
AtCPK18	At4g36070	1686	561	11	17056723-17059627
AtCPK19	At1g61950	1656	551	8	22899417-22901946
AtCPK20	At2g38910	1752	583	6	16245214-16247483
AtCPK21	At4g04720	1596	531	7	2394458-2397759
AtCPK22	At4g04710	1728	575	11	2389598-2392887
AtCPK23	At4g04740	1602	533	9	2403609-2408737
AtCPK24	At2g31500	1749	582	6	13413764-13416536
AtCPK25	At2g35890	1563	520	5	15067175-15069136
AtCPK26	At4g38230	1023	340	9	17928677-17931182
AtCPK27	At4g04700	1458	485	7	2385276-2387986
AtCPK28	At5g66210	1572	523	11	26456292-26459624
AtCPK29	At1g76040	972	323	7	28538830-28540637
AtCPK30	At1g74740	1626	541	6	28079946-28082644
AtCPK31	At4g04695	1455	484	7	2381634-2383996
AtCPK32	At3g57530	1617	538	7	21296554-2129959
AtCPK33	At1g50700	1566	521	7	18781914-187845582
AtCPK34	At5g19360	1572	523	7	6521716-6523780
Brachipodium distachyon					
BdCPK1-1	Bradi4g07280	1833	610	5	Bd4: 6192806 - 6195751
BdCPK1-2	Bradi1g56970	1704	567	7	Bd1: 55653556 - 55658016
BdCPK2	Bradi1g06300	1872	623	6	Bd1: 4224981 - 4228341
BdCPK3-1	Bradi2g43910	1581	526	7	Bd2: 44420445 - 44424115
BdCPK3-2	Bradi2g15520	1647	548	7	Bd2: 13793249 - 13796809
BdCPK5-1	Bradi5g19430	1686	561	6	Bd5: 22416441 - 22420844
BdCPK5-2	Bradi3g51970	1671	556	6	Bd3: 52938242 - 52943118
BdCPK5-3	Bradi3g60750	1599	532	1	Bd3: 59589913 - 59591641
BdCPK6	Bradi1g76560	1653	550	7	Bd1: 73098658 - 73104007
BdCPK7-1	Bradi4g39870	1713	570	5	Bd4: 44427886 - 44430105
BdCPK7-2	Bradi1g04440	1602	533	7	Bd1: 2995211 - 2999367
BdCPK7-3	Bradi3g41770	1740	579	2	Bd3: 43613029 - 43615285
BdCPK12	Bradi4g24390	1557	518	6	Bd4: 29529098 - 29532964
BdCPK13-1	Bradi2g54080	1686	516	5	Bd2: 53042608 - 53049672
BdCPK13-2	Bradi2g22750	1647	548	6	Bd2: 20263728 - 20269410
BdCPK16-1	Bradi1g52567	1551	516	11	Bd1: 50881886 - 50892879
BdCPK16-2	Bradi3g02600	1563	520	11	Bd3: 1572166 - 1578366
BdCPK17-1	Bradi2g52870	1545	514	5	Bd2: 52129651 - 52132367
BdCPK17-2	Bradi4g26317	1599	532	4	Bd4: 31567408 - 31569545

BdCPK20	Bradi1g06270	1773	590	6	Bd1: 4206012 - 4209237
BdCPK24	Bradi4g35100	1698	565	7	Bd4: 40589474 - 40592706
BdCPK29	Bradi5g18250	1719	572	6	Bd5: 21294347 - 21296831
BdCPK30	Bradi1g12150	1863	620	5	Bd1: 9069968 - 9073822
BdCPK32	Bradi1g24240	1617	538	7	Bd1: 19458483 - 19462136
BdCPK33	Bradi1g26310	1599	532	8	Bd1: 21360318 - 21364935
BdCPK34-1	Bradi2g21390	1551	516	6	Bd2: 18759409 - 18761785
BdCPK34-2	Bradi4g43400	1650	549	5	Bd4: 47105422 - 47107635
Brassica rapa					
BrCPK1	Bra009420	1776	591	6	A10: 16553383 - 16555719
BrCPK2	Bra005824	1821	606	6	A03: 800326 - 802668
BrCPK3-1	Bra013719	1578	525	8	A01: 7369320 - 7371687
BrCPK3-2	Bra019282	1131	376	6	A03: 25259172 - 25260753
BrCPK3-3	Bra019281	1599	532	7	A03: 25265161 - 25267624
BrCPK3-4	Bra019284	1599	532	7	A03: 25249152 - 25251736
BrCPK4	Bra000684	1503	500	6	A03: 12448278 - 12450784
BrCPK5	Bra011605	1638	545	6	A01: 1439332 - 1441949
BrCPK6-1	Bra009653	1620	539	6	A06: 16400117 - 16402382
BrCPK6-2	Bra037277	1656	551	6	A09: 4581746 - 4584184
BrCPK7	Bra008879	1599	532	7	A10: 14254455 - 14256612
BrCPK8-1	Bra020040	1605	534	7	A02: 4772074 - 4774454
BrCPK8-2	Bra002237	1437	478	7	A10: 10734221 – 10736321
BrCPK9	Bra001789	1629	542	7	A03: 18475915 - 18478189
BrCPK10-1	Bra031055	1596	531	6	A09: 32720807 - 32723204
BrCPK10-2	Bra025696	1587	528	7	A06: 7355217 - 7357590
BrCPK11-1	Bra034407	1494	497	6	A05: 13550366 - 13552447
BrCPK11-2	Bra029376	1389	462	6	A02: 25553588 - 25555713
BrCPK11-3	Bra002324	1485	494	5	A10: 10308833 - 10311211
BrCPK11-4	Bra029377	1281	426	5	A02: 25549908 - 25551605
BrCPK12	Bra026489	1461	486	5	A01: 9022707 - 9024990
BrCPK13-1	Bra033476	1587	528	6	A04: 4434076 - 4436992
BrCPK13-2	Bra012835	1587	528	6	A03: 21999149 - 22001348
BrCPK13-3	Bra006881	1587	528	6	09: 25958833 - 25961718
BrCPK14-1	Bra004656	1599	532	7	A05: 1262159 - 1264378
BrCPK14-2	Bra016909	1596	531	7	A04: 17648913 - 17651206
BrCPK15	Bra013575	1722	573	7	A01: 6415699 - 6418093
BrCPK16	Bra024487	1746	581	11	A06: 16161826 - 16164574
BrCPK17-1	Bra008910	1569	522	5	A10: 14375701 - 14377722
BrCPK17-2	Bra006142	1572	523	5	A03: 2206432 - 2208421
BrCPK17-3	Bra029378	1455	484	5	A02: 25545790 - 25547700

BrCPK18	Bra017746	1626	541	11	A03: 30175277 - 30177826
BrCPK20	Bra000105	1701	566	6	A03: 9258803 - 9261302
BrCPK21	Bra018504	1563	520	7	A05: 8746727 - 8749271
BrCPK22-1	Bra029513	1446	481	7	A09: 17441806 - 17444210
BrCPK22-2	Bra018243	1290	429	6	A05: 6898049 - 6900236
BrCPK24-1	Bra022844	1653	550	7	A03: 7369722 - 7372562
BrCPK24-2	Bra018236	1755	584	6	A05: 6866892 - 6869171
BrCPK24-3	Bra021727	1770	589	7	A04: 14134549 - 14140977
BrCPK26	Bra033557	2688	895	22	A06: 26242459 - 26248389
BrCPK28-1	Bra012058	1596	531	10	A07: 10255800 - 10258550
BrCPK28-2	Bra037181	1617	538	11	A09: 4119191 - 4122002
BrCPK29	Bra015796	1584	527	7	A07: 20822804 - 20825575
BrCPK30	Bra015896	1641	546	6	A07: 20322205 - 20324492
BrCPK32-1	Bra003287	1617	538	7	A07: 12391920 - 12394546
BrCPK32-2	Bra007334	1626	541	6	A09: 28662952 - 28665651
BrCPK34-1	Bra020035	1554	517	6	A02: 4736958 - 4739051
BrCPK34-2	Bra002231	1554	517	6	A10: 10762241 - 10764364
BrCPK34-3	Bra023367	1542	513	5	A02: 3030905 - 3033974
Capsella rubella					
CrCPK1	Carubv10000481m.g	1857	618	6	scaffold_6: 1398132 - 1401988
CrCPK4	Carubv10000785m.g	1506	501	6	scaffold_6: 12666073 - 12670696
CrCPK5	Carubv10004491m.g	1683	560	7	scaffold_7: 1857981 - 1861084
CrCPK6	Carubv10013380m.g	1656	551	7	scaffold_3: 13259724 - 13262976
CrCPK7	Carubv10002666m.g	1605	534	7	scaffold_6: 4042899 - 4045162
CrCPK8	Carubv10000678m.g	1602	533	7	scaffold_6: 6593705 - 6596759
CrCPK9	Carubv10013403m.g	1623	540	7	scaffold_3: 7294459 - 7297724
CrCPK10	Carubv10008777m.g	1638	545	6	scaffold_1: 6561208 - 6563986
CrCPK11	Carubv10008949m.g	1488	495	6	scaffold_1: 12721313 - 12724127
CrCPK12	Carubv10000821m.g	1473	490	5	scaffold_6: 7998079 - 8001808
CrCPK13	Carubv10017005m.g	1587	528	6	scaffold_5: 9323847 - 9327476
CrCPK14	Carubv10025074m.g	1596	531	7	scaffold_4: 12600940 - 12603196
CrCPK15	Carubv10004493m.g	1680	559	7	scaffold_7: 7270469 - 7273008
CrCPK16	Carubv10013325m.g	1728	575	11	scaffold_3: 13557709 - 13560878
CrCPK17	Carubv10002812m.g	1593	530	7	scaffold_6: 3915517 - 3917828
CrCPK18	Carubv10006766m.g	1614	537	11	scaffold_7: 1582707 - 1585412
CrCPK19	Carubv10021616m.g	1644	547	8	scaffold_2: 1297246 - 1299697
CrCPK20	Carubv10025314m.g	1764	587	6	scaffold_4: 11324785 - 11327192
CrCPK21	Carubv10003219m.g	1605	534	7	scaffold_6: 14750393 - 14753274
CrCPK22	Carubv10000880m.g	1416	471	7	scaffold_6: 14746578 - 14749038
CrCPK23	Carubv10000714m.g	1575	524	7	scaffold_6: 14753960 - 14757238

CrCPK24	Carubv10025444m.g	1749	582	6	scaffold_4: 8193186 - 8195489
CrCPK26	Carubv10006526m.g	1422	473	7	scaffold_7: 58230 - 60139
CrCPK27	Carubv10003655m.g	1458	485	7	scaffold_6: 14743451 - 14746057
CrCPK28	Carubv10028399m.g	1617	538	11	scaffold_8: 13260287 - 13263195
CrCPK29	Carubv10020082m.g	1671	556	7	scaffold_2: 12188681 - 12191676
CrCPK30	Carubv10020106m.g	1626	541	6	scaffold_2: 11662872 - 11666860
CrCPK31	Carubv10000863m.g	1431	476	7	scaffold_6: 14738146 - 14740836
CrCPK32-1	Carubv10016977m.g	1620	539	8	scaffold_5: 11579109 - 11582260
CrCPK32-2	Carubv10004572m.g	1590	529	8	scaffold_7: 6537980 - 6540943
CrCPK33	Carubv10008860m.g	1563	520	7	scaffold_1: 17521468 - 17524839
CrCPK34	Carubv10003311m.g	1575	524	6	scaffold_6: 6556576 - 6558648
Carica papaya					
CpCPK1-1	evm.TU.supercontig_33.122	1665	554	7	supercontig_33: 1223982 - 1231453
CpCPK1-2	evm.TU.supercontig_222.24	1872	623	6	supercontig_222: 178494 - 181993
CpCPK2	evm.TU.supercontig_3109.1	693	231	3	supercontig_3109: 884 - 4012
CpCPK3	evm.TU.supercontig_122.15	1587	528	8	supercontig_122: 261246 - 265316
CpCPK5	evm.TU.supercontig_60.3	1701	566	6	supercontig_60: 25465 - 30280
CpCPK7	evm.TU.supercontig_84.46	1596	531	7	supercontig_84: 398823 - 402681
CpCPK10	evm.TU.supercontig_17.194	1644	547	6	supercontig_17: 2348757 - 2355707
CpCPK12	evm.TU.supercontig_138.29	1365	454	5	supercontig_138: 383860 - 388473
CpCPK16	evm.TU.supercontig_6.254	1527	508	9	supercontig_6: 1999150 - 2003214
CpCPK17	evm.TU.supercontig_157.56	1587	528	7	supercontig_157: 363288 - 365889
CpCPK21	evm.TU.supercontig_152.12	1650	549	7	supercontig_152: 69818 - 74332
CpCPK24	evm.TU.contig_33100	1758	585	7	contig_33100: 494 - 3895
CpCPK29	evm.TU.supercontig_26.269	1554	517	8	supercontig_26: 1816278 - 1821059
CpCPK32	evm.TU.supercontig_92.31	1602	533	7	supercontig_92: 295164 - 299815
CpCPK33	evm.TU.supercontig_12.305	1581	526	7	supercontig_12: 2790567 - 2794974
Chlamydomonas reinhardtii					
CreinCPK2	Cre01.g009500	2295	764	13	chromosome_1: 1763026 - 1771764
CreinCPK4	Cre13.g564500	3129	1042	13	chromosome_13: 392824 - 400949
CreinCPK9	Cre17.g705000	1842	613	11	chromosome_17: 1209839 - 1214698
CreinCPK12	g2888	1710	569	13	chromosome_3: 267426 - 273277
CreinCPK17-1	Cre08.g382800	2061	686	14	chromosome_8: 4548983 - 4557157
CreinCPK17-2	Cre07.g328900	1455	484	9	chromosome_7: 2426734 - 2431805
CreinCPK17-3	Cre02.g074370	5403	1800	15	chromosome_2: 163680 - 173615
CreinCPK17-4	Cre02.g114750	3126	1041	16	chromosome_2: 6297945 - 6306066
CreinCPK17-5	Cre02.g106650	5940	1979	11	chromosome_2: 5287410 - 5296386
CreinCPK17-6	g86	2202	733	11	chromosome_1: 653607 - 662145
CreinCPK17-7	Cre10.g418900	5490	1829	14	chromosome_10: 161899 - 172025
CreinCPK20-1	Cre06.g265550	1908	635	13	chromosome_6: 2117499 - 2123655

CreinCPK20-2	Cre13.g571700	1515	504	9	chromosome_13: 1366004 - 1370421
CreinCPK34	g18129	1626	541	15	scaffold_19: 78930 - 89155
Citrus clememtina					
CcCPK1	Ciclev10014759m.g	1701	566	7	scaffold_2: 4222562 - 4227899
CcCPK3	Ciclev10019724m.g	1563	520	7	scaffold_3: 1318119 - 1321956
CcCPK4	Ciclev10000931m.g	1503	500	6	scaffold_5: 39415804 - 39419710
CcCPK8	Ciclev10014873m.g	1596	531	8	scaffold_2: 6915377 - 6920275
CcCPK9-1	Ciclev10010314m.g	1482	494	7	scaffold_1: 22255893 - 22258660
CcCPK9-2	Ciclev10007978m.g	1599	532	7	scaffold_1: 22145495 - 22148716
CcCPK9-3	Ciclev10007983m.g	1590	529	7	scaffold_1: 22378859 - 22384122
CcCPK9-4	Ciclev10010522m.g	1368	456	6	scaffold_1: 22149811 - 22152338
CcCPK10	Ciclev10019541m.g	1674	557	6	scaffold_3: 2375432 - 2379089
CcCPK12	Ciclev10025418m.g	1518	505	6	scaffold_7: 5815049 - 5819452
CcCPK13	Ciclev10011471m.g	1584	527	6	scaffold_6: 16445359 - 16450818
CcCPK15	Ciclev10014823m.g	1635	544	7	scaffold_2: 35323615 - 35328102
CcCPK17	Ciclev10014854m.g	1605	534	7	scaffold_2: 8049816 - 8052326
CcCPK20	Ciclev10011369m.g	1728	575	6	scaffold_6: 19558582 - 19562546
CcCPK24	Ciclev10024502m.g	1617	538	8	scaffold_3: 39048548 - 39050973
CcCPK25	Ciclev10011252m.g	1962	653	6	scaffold_6: 19641520 - 19644834
CcCPK26	Ciclev10028110m.g	1695	564	8	scaffold_8: 138022 - 144197
CcCPK28	Ciclev10025251m.g	1734	577	11	scaffold_7: 995485 - 1000767
CcCPK29	Ciclev10000897m.g	1545	514	7	scaffold_5: 42272203 - 42276387
CcCPK32	Ciclev10004707m.g	1599	532	7	scaffold_9: 25094127 - 25099067
CcCPK33-1	Ciclev10010666m.g	1476	491	7	scaffold_1: 22260261 - 22263022
CcCPK33-2	Ciclev10010555m.g	1509	502	7	scaffold_1: 22289913 - 22294246
CcCPK33-3	Ciclev10007950m.g	1617	538	8	scaffold_1: 22185234 - 22188147
CcCPK33-4	Ciclev10007980m.g	1596	531	7	scaffold_1: 22138397 - 22141621
CcCPK33-5	Ciclev10007873m.g	1701	566	7	scaffold_1: 22281550 - 22284439
CcCPK33-6	Ciclev10008020m.g	1551	516	7	scaffold_1: 22168260 - 22171495
Citrus sinensis					
CsCPK1	orange1.1g007353m.g	1824	607	7	scaffold00263: 4410 - 9622
CsCPK3	orange1.1g010013m.g	1563	520	7	scaffold00009: 1154077 - 1157828
CsCPK8	orange1.1g009594m.g	1596	531	8	scaffold00054: 367742 - 372716
CsCPK9-1	orange1.1g009658m.g	1590	529	7	scaffold00046: 632250 - 637786
CsCPK9-2	orange1.1g042823m.g	1434	477	6	scaffold00937: 8746 - 13646
CsCPK9-3	orange1.1g009561m.g	1599	532	7	scaffold00046: 505676 - 508945
CsCPK9-4	orange1.1g047606m.g	1431	476	8	scaffold06547: 380 - 3140
CsCPK11	orange1.1g010806m.g	1503	500	6	scaffold00002: 1363743 - 1367520
CsCPK12	orange1.1g014949m.g	1248	415	6	scaffold00074: 410910 - 415085
CsCPK13	orange1.1g009731m.g	1584	527	6	scaffold00098: 97202 - 102978

CsCPK20	orange1.1g040003m.g	1470	489	4	scaffold00018: 1021953 - 1025934
CsCPK24	orange1.1g043828m.g	1761	586	7	scaffold00501: 53103 - 55529
CsCPK25	orange1.1g006259m.g	1962	653	6	scaffold00018: 936132 - 939434
CsCPK26	orange1.1g007895m.g	1758	585	8	scaffold00023: 1346143 - 1351915
CsCPK28	orange1.1g008127m.g	1734	577	11	scaffold00017: 811565 - 816844
CsCPK29	orange1.1g010263m.g	1545	514	7	scaffold00002: 4146577 - 4150761
CsCPK30	orange1.1g008668m.g	1674	557	6	scaffold00009: 120619 - 124415
CsCPK31	orange1.1g009091m.g	1635	544	7	scaffold00007: 1770624 - 1775055
CsCPK32	orange1.1g009367m.g	1611	536	7	scaffold00137: 211172 - 216114
CsCPK33-1	orange1.1g018604m.g	1062	353	7	scaffold00046: 546981 - 549307
CsCPK33-2	orange1.1g009382m.g	1611	536	7	scaffold00046: 498575 - 501811
CsCPK33-3	orange1.1g040917m.g	1482	494	7	scaffold00046: 509992 - 512739
CsCPK33-4	orange1.1g010756m.g	1509	502	7	scaffold00046: 554215 - 557140
CsCPK33-5	orange1.1g010164m.g	1551	516	7	scaffold00046: 528713 - 531902
Coccomyxa subellipsoidea					
CsubCPK4	estExt_fgenesh1_pg.C_20259	1437	478	11	scaffold_2: 1892803 - 1896953
CsubCPK34	estExt_fgenesh1_pg.C_90258	1542	513	11	scaffold_9: 1804284 - 1810169
Cucumis sativus					
CsatCPK1	Cucsa.242110	1933	645	6	scaffold02047: 1919024 - 1922198
CsatCPK2	Cucsa.282830	1728	575	6	scaffold02653: 163430 - 167517
CsatCPK3	Cucsa.107040	1203	400	7	scaffold00930: 479422 - 484125
CsatCPK4-1	Cucsa.240680	1506	501	6	scaffold02047: 786114 - 797532
CsatCPK4-2	Cucsa.228290	1512	503	6	scaffold01933: 229341 - 233166
CsatCPK8-1	Cucsa.164370	1608	535	7	scaffold01148: 241178 - 245133
CsatCPK8-2	Cucsa.124160	1590	529	8	scaffold01001: 188864 - 192575
CsatCPK8-3	Cucsa.143420	1596	531	7	scaffold01079: 1767277 - 1771369
CsatCPK9	Cucsa.059990	1560	519	11	scaffold00621: 536471 - 541152
CsatCPK10	Cucsa.098010	1641	546	6	scaffold00923: 168087 - 173164
CsatCPK13	Cucsa.176420	1584	527	6	scaffold01225: 682977 - 686362
CsatCPK17	Cucsa.086940	1608	535	7	scaffold00873: 293942 - 296912
CsatCPK20	Cucsa.242210	1682	559	7	scaffold02047: 1989412 - 1994019
CsatCPK21	Cucsa.045250	1659	552	7	scaffold00542: 1235379 - 1239597
CsatCPK24	Cucsa.095230	1584	527	7	scaffold00919: 1146708 - 1151916
CsatCPK26	Cucsa.099800	1701	566	7	scaffold00926: 486587 - 492864
CsatCPK28	Cucsa.043570	1632	543	11	scaffold00542: 342686 - 347933
CsatCPK29	Cucsa.343230	1593	530	7	scaffold03356: 5489389 - 5493956
Eucalyptus grandis					
EgCPK1	Eucgr.G02664	1740	579	6	scaffold_7: 44881943 - 44886934

EgCPK3	Eucgr.E00806	1620	539	8	scaffold_5: 8323291 - 8329571
EgCPK4	Eucgr.F02611	1527	508	6	scaffold_6: 35773745 - 35780953
EgCPK8	Eucgr.J00760	1614	537	7	scaffold_10: 8009112 - 8013517
EgCPK10	Eucgr.K02914	1638	545	7	scaffold_11: 37040008 - 37045219
EgCPK13	Eucgr.A00519	1632	543	6	scaffold_1: 8323188 - 8333757
EgCPK14	Eucgr.A02375	1578	525	7	scaffold_1: 34534557 - 34539753
EgCPK20	Eucgr.A02545	1746	581	6	scaffold_1: 35997151 - 36003407
EgCPK21	Eucgr.E04057	1641	546	7	scaffold_5: 69657664 - 69662976
EgCPK24-1	Eucgr.A00262	1593	530	7	scaffold_1: 3257334 - 3260454
EgCPK24-2	Eucgr.A00058	1272	423	7	scaffold_1: 770558 - 773325
EgCPK25	Eucgr.A02554	1830	609	6	scaffold_1: 36052133 - 36057861
EgCPK26-1	Eucgr.I01536	1722	573	8	scaffold_9: 25419253 - 25424700
EgCPK26-2	Eucgr.H04992	1698	565	8	scaffold_8: 71501793 - 71507012
EgCPK28-1	Eucgr.J02432	1638	545	11	scaffold_10: 30394685 - 30401059
EgCPK28-2	Eucgr.I02347	1647	548	11	scaffold_9: 34009374 - 34015038
EgCPK29-1	Eucgr.L01282	1119	372	7	scaffold_166: 37484 - 42311
EgCPK29-2	Eucgr.F01695	1467	488	8	scaffold_6: 21676876 - 21681932
EgCPK29-3	Eucgr.F01694	1590	529	7	scaffold_6: 21601955 - 21607012
EgCPK33-1	Eucgr.F00807	1632	543	7	scaffold_6: 10649878 - 10654619
EgCPK33-2	Eucgr.F00761	1551	516	7	scaffold_6: 9982677 - 9987378
EgCPK34	Eucgr.J00686	1605	534	7	scaffold_10: 7357704 - 7360532
Fragaria vesca					
FvCPK3	gene31992-v1.0-hybrid	1647	548	8	LG5: 2203768 - 2208038
FvCPK7	gene25220-v1.0-hybrid	1650	549	7	LG6: 19324137 - 19327350
FvCPK8	gene14687-v1.0-hybrid	1566	521	7	LG2: 23420992 - 23425402
FvCPK9	gene19615-v1.0-hybrid	1632	543	7	LG3: 440791 - 444005
FvCPK11-1	gene27440-v1.0-hybrid	1473	490	6	LG2: 429217 - 432145
FvCPK11-2	gene05409-v1.0-hybrid	1497	498	0	LG6: 27459291 - 27460787
FvCPK13	gene13451-v1.0-hybrid	1584	527	6	LG6: 6842534 - 6846941
FvCPK20	gene18135-v1.0-hybrid	2130	709	9	LG6: 17846926 - 17855906
FvCPK25	gene18254-v1.0-hybrid	2127	708	7	LG6: 18045101 - 18049749
FvCPK26	gene17341-v1.0-hybrid	1707	568	6	LG2: 10179942 - 10183296
FvCPK28	gene14609-v1.0-hybrid	1653	550	11	LG1: 7913710 - 7917475
FvCPK29	gene08576-v1.0-hybrid	1671	556	6	LG2: 19182892 - 19186797
FvCPK33	gene03391-v1.0-hybrid	1626	541	7	LG3: 13177898 - 13182279
FvCPK34	gene15357-v1.0-hybrid	2043	680	9	LG2: 24311895 - 24319062
Glycine max					
GmCPK1	Glyma02g34890	1764	587	7	Gm02: 39528524 - 39533926
GmCPK2-1	Glyma10g10501	1779	592	7	Gm10: 10261765 - 10267452
GmCPK2-2	Glyma20g17020	1740	579	7	Gm20: 23983441 - 23991227

GmCPK3-1	Glyma11g02260	1518	505	7	Gm11: 1409681 - 1414565
GmCPK3-2	Glyma01g43241	1629	542	7	Gm01: 54296016 - 54301335
GmCPK3-3	Glyma05g37260	1557	518	7	Gm05: 40863765 - 40870172
GmCPK4-1	Glyma10g36100	1479	492	6	Gm10: 44273615 - 44278967
GmCPK4-2	Glyma20g31510	1443	480	7	Gm20: 40136153 - 40141010
GmCPK8-1	Glyma19g32260	1608	535	7	Gm19: 40020617 - 40025453
GmCPK8-2	Glyma10g17561	1587	528	8	Gm10: 21210074 - 21215805
GmCPK9-1	Glyma18g11030	1656	551	8	Gm18: 9856626 - 9862835
GmCPK9-2	Glyma08g42850	1656	551	7	Gm08: 42814225 - 42819950
GmCPK9-3	Glyma02g46070	1587	528	7	Gm02: 50154974 - 50159606
GmCPK10-1	Glyma06g20170	1656	551	6	Gm06: 16573644 - 16578709
GmCPK10-2	Glyma04g34440	1671	556	6	Gm04: 40634730 - 40640106
GmCPK11-1	Glyma06g16920	1494	497	6	Gm06: 13300048 - 13304817
GmCPK11-2	Glyma04g38150	1491	496	6	Gm04: 44537162 - 44541892
GmCPK11-3	Glyma08g00840	1527	508	6	Gm08: 447074 - 451302
GmCPK11-4	Glyma05g33240	1524	507	6	Gm05: 37961255 - 37967134
GmCPK13	Glyma07g18310	1602	533	6	Gm07: 18181060 - 18188910
GmCPK17-1	Glyma14g04010	1590	529	7	Gm14: 2671992 - 2676605
GmCPK17-2	Glyma02g44720	1584	527	7	Gm02: 49196816 - 49204238
GmCPK17-3	Glyma20g08140	1641	546	7	Gm20: 11310728 - 11316123
GmCPK17-4	Glyma07g36000	1626	541	7	Gm07: 41372556 - 41377582
GmCPK20	Glyma10g11020	1788	595	6	Gm10: 10963978 - 10971320
GmCPK21-1	Glyma17g01730	1617	538	7	Gm17: 1053574 - 1058136
GmCPK21-2	Glyma07g39010	1590	529	7	Gm07: 43606850 - 43611483
GmCPK24-1	Glyma12g05730	1602	533	7	Gm12: 3826087 - 3830846
GmCPK24-2	Glyma11g13740	1629	542	7	Gm11: 9760217 - 9765092
GmCPK26-1	Glyma14g00320	1677	558	7	Gm14: 99206 - 105370
GmCPK26-2	Glyma02g48160	1650	549	8	Gm02: 51587966 - 51594546
GmCPK28-1	Glyma02g05440	1593	530	13	Gm02: 4375744 - 4382615
GmCPK28-2	Glyma11g08180	1689	562	11	Gm11: 5786857 - 5792495
GmCPK29-1	Glyma14g40090	1614	537	7	Gm14: 49077673 - 49082234
GmCPK29-2	Glyma17g38040	1752	583	8	Gm17: 41743259 - 41747531
GmCPK30-1	Glyma17g10410	1626	541	7	Gm17: 7787208 - 7794046
GmCPK30-2	Glyma05g01470	1620	539	6	Gm05: 991630 - 995684
GmCPK30-3	Glyma18g43160	1539	512	7	Gm18: 52541810 - 52550232
GmCPK32-1	Glyma02g31490	1578	525	8	Gm02: 34473389 - 34481404
GmCPK32-2	Glyma03g29450	1605	534	7	Gm03: 37436587 - 37441142
GmCPK33	Glyma14g02680	1620	539	7	Gm14: 1667923 - 1672565
Gossipium raimondi					
GrCPK1-1	Gorai.013G003100	1740	579	8	Chr13: 223969 - 228213

GrCPK1-2	Gorai.011G228500	1764	587	7	Chr11: 54159715 - 54164809
GrCPK1-3	Gorai.006G147600	1836	611	6	Chr06: 40605660 - 40609219
GrCPK2-1	Gorai.009G394700	1947	648	6	Chr09: 54483136 - 54487811
GrCPK2-2	Gorai.002G153600	1776	591	6	Chr02: 31402668 - 31405631
GrCPK3-1	Gorai.007G025000	1593	530	8	Chr07: 1749594 - 1753050
GrCPK3-2	Gorai.003G084000	1527	508	9	Chr03: 21113101 - 21119320
GrCPK3-3	Gorai.008G251000	1605	534	8	Chr08: 53485474 - 53489684
GrCPK4	Gorai.009G290200	1617	538	7	Chr09: 24957004 - 24962720
GrCPK6-1	Gorai.013G064400	1464	487	8	Chr13: 7213133 - 7217520
GrCPK6-2	Gorai.013G064500	1572	523	8	Chr13: 7231674 - 7235023
GrCPK6-3	Gorai.005G216500	1707	568	8	Chr05: 59941505 - 59946021
GrCPK6-4	Gorai.008G013700	1728	575	6	Chr08: 1576504 - 1579473
GrCPK6-5	Gorai.012G138900	1659	552	8	Chr12: 31259337 - 31263004
GrCPK7-1	Gorai.006G128200	1596	531	8	Chr06: 38135027 - 38139699
GrCPK7-2	Gorai.006G137800	1596	531	7	Chr06: 39398716 - 39402517
GrCPK8	Gorai.001G138000	1590	529	8	Chr01: 18239686 - 18243609
GrCPK9-1	Gorai.002G088800	1614	537	8	Chr02: 11253285 - 11257287
GrCPK9-2	Gorai.013G159300	1611	536	7	Chr13: 43588966 - 43593714
GrCPK9-3	Gorai.009G438300	1575	524	7	Chr09: 68877152 - 68880949
GrCPK10	Gorai.007G035100	1653	550	7	Chr07: 2415302 - 2419087
GrCPK11-1	Gorai.005G074300	1527	508	6	Chr05: 8240260 - 8243421
GrCPK11-2	Gorai.012G045700	1494	497	6	Chr12: 5721482 - 5727068
GrCPK13-1	Gorai.012G114600	1584	527	6	Chr12: 26404347 - 26409940
GrCPK13-2	Gorai.007G378700	1584	527	6	Chr07: 60894830 - 60901865
GrCPK17-1	Gorai.009G078000	1599	532	7	Chr09: 5620081 - 5622436
GrCPK17-2	Gorai.004G015100	1617	538	7	Chr04: 1083972 - 1087141
GrCPK17-3	Gorai.013G253100	1584	527	7	Chr13: 57043788 - 57049048
GrCPK17-4	Gorai.001G135000	1542	513	7	Chr01: 17623836 - 17626200
GrCPK17-5	Gorai.006G124800	1575	524	7	Chr06: 37646865 - 37649247
GrCPK20	Gorai.009G395400	1719	572	9	Chr09: 54682886 - 54690651
GrCPK21-1	Gorai.010G252400	1584	527	7	Chr10: 61853992 - 61856356
GrCPK21-2	Gorai.011G014200	1656	551	7	Chr11: 999465 - 1003347
GrCPK24-1	Gorai.005G019800	1599	532	7	Chr05: 1578906 - 1582704
GrCPK24-2	Gorai.009G351200	1605	534	7	Chr09: 44818826 - 44821159
GrCPK28-1	Gorai.011G098300	1635	544	11	Chr11: 10914490 - 10918887
GrCPK28-2	Gorai.003G009500	1626	541	11	Chr03: 578105 - 583466
GrCPK28-3	Gorai.007G194500	1665	554	11	Chr07: 19220289 - 19225229
GrCPK29	Gorai.010G001300	1581	526	7	Chr10: 47227 - 50465
GrCPK32	Gorai.003G092900	1608	535	7	Chr03: 28230106 - 28234296
Linum usitatissimum					

LuCPK1-1	Lus10028862.g	1797	598	6	scaffold540: 602812 - 605766
LuCPK1-2	Lus10008958.g	1485	494	6	scaffold1486: 203055 - 205723
LuCPK2-1	Lus10029358.g	1275	424	6	scaffold360: 819450 - 821313
LuCPK2-2	Lus10013603.g	1944	647	6	scaffold62: 486181 - 488689
LuCPK2-3	Lus10021248.g	1944	647	6	scaffold1123: 119585 - 122102
LuCPK3	Lus10017537.g	1380	459	7	scaffold1253: 497561 - 499718
LuCPK4	Lus10009427.g	1485	494	6	scaffold981: 56873 - 59352
LuCPK6-1	Lus10026559.g	1647	548	6	scaffold617: 398195 - 400675
LuCPK6-2	Lus10013842.g	1647	548	6	scaffold618: 251287 - 253727
LuCPK6-3	Lus10001384.g	1698	565	6	scaffold1851: 4093 - 7257
LuCPK6-4	Lus10022986.g	1692	563	6	scaffold355: 579564 - 582663
LuCPK7	Lus10025528.g	1074	357	7	scaffold145: 215542 - 217531
LuCPK8-1	Lus10027361.g	1599	532	7	scaffold472: 937024 - 939529
LuCPK8-2	Lus10014907.g	2316	771	9	scaffold2022: 15486 - 18937
LuCPK8-3	Lus10009947.g	1629	542	7	scaffold200: 272828 - 275268
LuCPK8-4	Lus10030134.g	1503	500	6	scaffold19: 1058953 - 1061081
LuCPK9-1	Lus10021531.g	1623	540	7	scaffold362: 209020 - 211373
LuCPK9-2	Lus10040071.g	1623	540	7	scaffold12: 2147759 - 2150105
LuCPK9-3	Lus10032640.g	1545	514	7	scaffold140: 1160193 - 1162287
LuCPK10	Lus10008631.g	1689	562	6	scaffold1686: 236708 - 239479
LuCPK11-1	Lus10017911.g	1476	491	6	scaffold116: 171167 - 173492
LuCPK11-2	Lus10014820.g	1476	491	6	scaffold184: 391548 - 394315
LuCPK11-3	Lus10029547.g	1539	512	6	scaffold55: 975680 - 978412
LuCPK11-4	Lus10039623.g	1542	513	6	scaffold15: 804474 - 807232
LuCPK13-1	Lus10004807.g	1584	527	6	scaffold426: 23227 - 25882
LuCPK13-2	Lus10002482.g	1584	527	6	scaffold1199: 27206 - 29812
LuCPK13-3	Lus10027808.g	1587	528	6	scaffold1143: 467290 - 470125
LuCPK13-4	Lus10005038.g	1269	422	6	scaffold1982: 37301 - 39821
LuCPK16	Lus10028459.g	1476	491	11	scaffold413: 763968 - 767236
LuCPK17-1	Lus10036050.g	1578	525	6	scaffold76: 925735 - 929121
LuCPK17-2	Lus10000889.g	1578	525	6	scaffold1936: 9117 - 12066
LsCPK20-1	Lus10016200.g	1806	601	6	scaffold947: 174031 - 176366
LuCPK20-2	Lus10012285.g	1728	575	6	scaffold273: 394970 - 397558
LuCPK20-3	Lus10015992.g	1725	574	6	scaffold172: 141684 - 144214
LuCPK20-4	Lus10029346.g	1695	564	6	scaffold360: 768893 - 771118
LuCPK21-1	Lus10006777.g	1581	526	7	scaffold204: 247611 - 250506
LuCPK21-2	Lus10002075.g	900	299	6	scaffold1302: 76028 - 77709
LuCPK21-3	Lus10020046.g	1617	538	7	scaffold23: 569436 - 572590
LuCPK24	Lus10027032.g	1599	532	7	scaffold297: 185406 - 187642
LuCPK28	Lus10041914.g	1719	572	11	scaffold272: 2365224 - 2368778

LuCPK29	Lus10017251.g	1551	516	6	scaffold117: 383430 - 386586
LuCPK30-1	Lus10036667.g	1500	499	5	scaffold57: 1744983 - 1747355
LuCPK30-2	Lus10042185.g	1296	431	6	scaffold123: 1096157 - 1098461
LuCPK32-1	Lus10042370.g	1584	527	5	scaffold123: 1870621 - 1873700
LuCPK32-2	Lus10026742.g	1530	509	7	scaffold361: 291797 - 294313
LuCPK32-3	Lus10025570.g	2079	692	8	scaffold145: 445899 - 448852
LuCPK34	Lus10038460.g	1605	534	7	scaffold28: 1452060 - 1456326
Malus domestica					
MdCPK1-1	MDP0000153100	1701	566	6	Chr2: MDC017159.84: 8453 - 14350
MdCPK1-2	MDP0000142687	1857	618	8	Chr7: MDC021045.283: 1756 - 8958
MdCPK1-3	MDP0000128057	1983	660	8	Chr7: MDC013839.354: 42 - 713
MdCPK2	MDP0000232344	2328	775	8	Chr12: MDC012227.366: 34198 - 38360
MdCPK4-1	MDP0000260834	1554	517	6	Chr9: MDC020449.143: 14625 - 18251
MdCPK4-2	MDP0000232885	1557	518	6	Chr10: MDC010220.255: 18291 - 21903
MdCPK8-1	MDP0000269423	1662	553	8	Chr2: MDC001073.515: 2333 - 8854
MdCPK8-2	MDP0000119457	1431	476	6	Chr15: MDC001073.498: 3281 - 6157
MdCPK8-3	MDP0000260857	1998	665	9	Chr12: MDC021346.204: 29191 - 34837
MdCPK10-1	MDP0000218522	1713	570	7	Chr6: MDC020438.169: 10660 - 14149
MdCPK10-2	MDP0000301254	1647	548	7	MDC016267.124: 15630 - 19053
MdCPK10-3	MDP0000308706	1647	548	7	MDC020438.160: 35695 - 39116
MdCPK11	MDP0000494270	1497	498	0	Chr9: MDC010082.361: 3158 - 4654
MdCPK13-1	MDP0000164868	1758	585	8	Chr4: MDC000306.525: 1570 - 6773
MdCPK13-2	MDP0000649496	1038	345	4	Chr13: MDC000271.449: 354 - 2825
MdCPK17-1	MDP0000802997	1602	533	7	Chr8: MDC040478.10: 1862 - 4930
MdCPK17-2	MDP0000138436	1605	534	7	MDC010071.376: 1022 - 3758
MdCPK20-1	MDP0000318339	3072	1023	10	Chr14: MDC031256.8: 21258 - 31086
MdCPK20-2	MDP0000513005	2040	679	7	Chr12: MDC008272.442: 6235 - 19231
MdCPK21	MDP0000232001	1665	554	7	Chr5: MDC002417.261: 24324 - 28052
MdCPK24-1	MDP0000262701	1626	541	7	Chr17: MDC020007.246: 24451 - 27032
MdCPK24-2	MDP0000282003	2865	954	12	Chr9: MDC006465.419: 8202 - 16334
MdCPK26-1	MDP0000297184	1716	571	6	Chr8: MDC012276.352: 7244 - 10346
MdCPK26-2	MDP0000457940	4212	1403	8	Chr8: MDC001323.383: 1559 - 7846
MdCPK28	MDP0000208913	1881	626	13	Chr2: MDC018730.149: 4526 - 9378
MdCPK29	MDP0000142398	1584	527	7	Chr12: MDC015573.110: 52421 - 55302
MdCPK32-1	MDP0000649508	2130	709	10	Chr15: MDC001801.279: 799 - 8644
MdCPK32-2	MDP0000179069	2031	676	10	Chr14: MDC006959.379: 1716 - 6520
Manihot esculenta					
MeCPK2	cassava4.1_004466m.g	1725	574	6	scaffold12865: 416791 - 425739
MeCPK3	cassava4.1_005320m.g	1599	532	8	scaffold05280: 745850 - 751642
MeCPK6-1	cassava4.1_004779m.g	1677	558	8	scaffold06582: 496693 - 502890

MeCPK6-2	cassava4.1_031489m.g	1677	558	6	scaffold02040: 210153 - 214213
MeCPK8	cassava4.1_005260m.g	1608	535	8	scaffold04681: 369192 - 373512
MeCPK11-1	cassava4.1_006083m.g	1506	501	6	scaffold00467: 131724 - 136606
MeCPK11-2	cassava4.1_005808m.g	1536	511	6	scaffold06814: 209569 - 213530
MeCPK12-1	cassava4.1_006026m.g	1512	503	6	scaffold00853: 48033 - 52094
MeCPK12-2	cassava4.1_006051m.g	1509	502	6	scaffold02811: 52985 - 57215
MeCPK17-1	cassava4.1_023323m.g	1587	528	7	scaffold07991: 65801 - 68074
MeCPK17-2	cassava4.1_029242m.g	1590	529	8	scaffold06916: 982685 - 986060
MeCPK20-1	cassava4.1_022914m.g	867	288	5	scaffold08542: 532736 - 535580
MeCPK20-2	cassava4.1_023345m.g	921	306	6	scaffold03777: 7117 - 10085
MeCPK21	cassava4.1_007132m.g	1395	464	7	scaffold06598: 72093 - 77621
MeCPK24-1	cassava4.1_021196m.g	1614	537	8	scaffold12121: 105306 - 108270
MeCPK24-2	cassava4.1_031848m.g	1587	528	7	scaffold06754: 43023 - 46027
MeCPK25	cassava4.1_029823m.g	1935	644	6	scaffold08542: 688509 - 691868
MeCPK28-1	cassava4.1_004577m.g	1707	568	11	scaffold06512: 925585 - 931029
MeCPK28-2	cassava4.1_004333m.g	1749	582	11	scaffold07520: 2142815 - 2148597
MeCPK29	cassava4.1_007356m.g	1371	456	6	scaffold02943: 457050 - 460665
MeCPK30	cassava4.1_004922m.g	1650	549	6	scaffold08359: 2362765 - 2367763
MeCPK32-1	cassava4.1_005336m.g	1596	531	7	scaffold02242: 186390 - 192047
MeCPK32-2	cassava4.1_005345m.g	1596	531	7	scaffold04075: 8822 - 13029
MeCPK32-3	cassava4.1_005177m.g	1617	538	8	scaffold10963: 249488 - 253958
MeCPK33-1	cassava4.1_005266m.g	1605	534	7	scaffold06278: 662482 - 666321
MeCPK33-2	cassava4.1_005323m.g	1599	532	8	scaffold03241: 233445 - 237541
Medicago truncatula					
MtCPK1	Medtr1g041150	1746	581	7	chr1: 11390665 - 11395730
MtCPK3	Medtr5g009830	1554	517	7	chr5: 2241283 - 2246294
MtCPK4	Medtr3g098090	1644	547	7	chr3: 33885378 - 33889054
MtCPK11-1	Medtr8g095440	1512	503	6	chr8: 27758630 - 27763068
MtCPK11-2	Medtr3g098070	1488	495	6	chr3: 33875230 - 33880120
MtCPK17-1	Medtr5g089320	1572	523	7	chr5: 37769598 - 37772048
MtCPK17-2	Medtr7g054260	1605	534	7	chr7: 14208505 - 14212434
MtCPK21	Medtr4g132070	1620	539	7	chr4: 46622087 - 46626785
MtCPK26	Medtr5g099240	1794	597	9	chr5: 42462792 - 42469958
MtCPK28	Medtr5g022030	1683	560	11	chr5: 8372103 - 8377363
MtCPK33	Medtr4g132040	1632	543	7	chr4: 46605619 - 46608392
Micromonas pusila CCMP1545					
MpCPK7	e_gw1.4.726.1	2217	738	1	scaffold_4: 113676 - 116037
MpCPK17	estExt_fgenesh2_kg.C_10200	1494	497	2	scaffold_1: 2000811 - 2002754
Mimulus guttatus					
MgCPK1	mgv1a003630m.g	1719	572	8	scaffold_8: 1804856 - 1808844

MgCPK3	mgv1a004683m.g	1548	515	7	scaffold_60: 867297 - 871330
MgCPK4	mgv1a004202m.g	1620	539	6	scaffold_3: 4250105 - 4253516
MgCPK6	mgv1a004107m.g	1632	543	8	scaffold_15: 549865 - 553622
MgCPK8	mgv1a004319m.g	1602	533	8	scaffold_290: 51824 - 55030
MgCPK9	mgv1a004414m.g	1587	528	7	scaffold_4: 244744 - 248080
MgCPK10-1	mgv1a004139m.g	1629	542	6	scaffold_55: 994225 - 998009
MgCPK10-2	mgv1a003708m.g	1707	568	7	scaffold_20: 2122937 - 2128410
MgCPK11-1	mgv1a005115m.g	1491	496	6	scaffold_64: 683986 - 686191
MgCPK11-2	mgv1a005313m.g	1470	489	6	scaffold_58: 1400866 - 1403788
MgCPK13-1	mgv1a004415m.g	1587	528	6	scaffold_44: 1270256 - 1274151
MgCPK13-2	mgv1a004417m.g	1587	528	6	scaffold_13: 1923601 - 1928146
MgCPK16	mgv1a007109m.g	1260	419	11	scaffold_31: 1297007 - 1301241
MgCPK17-1	mgv1a004352m.g	1596	531	6	scaffold_1: 1653860 - 1656446
MgCPK17-2	mgv1a004350m.g	1599	532	7	scaffold_2: 214993 - 217563
MgCPK20-1	mgv1a003550m.g	1737	578	6	scaffold_17: 2077752 - 2080004
MgCPK20-2	mgv1a003552m.g	1737	578	7	scaffold_90: 555149 - 558454
MgCPK24	mgv1a004648m.g	1551	516	7	scaffold_2: 4026521 - 4028849
MgCPK25	mgv1a002855m.g	1893	630	6	scaffold_17: 2117747 - 2120631
MgCPK28	mgv1a003874m.g	1677	558	11	scaffold_77: 923008 - 926823
MgCPK29	mgv1a004597m.g	1557	518	7	scaffold_98: 944128 - 947423
MgCPK32-1	mgv1a004318m.g	1602	533	7	scaffold_1: 1971833 - 1974678
MgCPK32-2	mgv1a004372m.g	1593	530	7	scaffold_87: 983656 - 986979
MgCPK32-3	mgv1a004430m.g	1584	527	7	scaffold_75: 377100 - 379683
MgCPK33	mgv1a004201m.g	1620	539	7	scaffold_24: 1860873 - 1864525
Oryza sativa					
OsCPK1	LOC_Os01g43410	1557	519	7	Chr1: 24839124-24843618
OsCPK2	LOC_Os01g59360	1548	516	7	Chr1: 34325310-34328118
OsCPK3	LOC_Os01g61590	1656	552	6	Chr1: 35619684-35625793
OsCPK4	LOC_Os02g03410	1569	523	11	Chr2: 1387873-1394417
OsCPK5	LOC_Os02g46090	1650	550	6	Chr2: 28086381-28092152
OsCPK6	LOC_Os02g58520	1638	546	2	Chr2: 35775030-35777266
OsCPK7	LOC_Os03g03660	1695	565	7	Chr3: 1625098-1629506
OsCPK8	LOC_Os03g59390	1617	539	7	Chr3: 33805800-33810099
OsCPK9	LOC_Os03g48270	1725	575	4	Chr3: 27467403-27472759
OsCPK10	LOC_Os03g57450	1800	600	6	Chr3: 32751649-3275567
OsCPK11	LOC_Os03g57510	1731	577	6	Chr3: 32794990-32791645
OsCPK12	LOC_Os04g47300	1602	534	7	Chr4: 28082320-28079505
OsCPK13	LOC_Os04g49510	1656	552	6	Chr4: 29531223-29536492
OsCPK14	LOC_Os05g41270	1569	523	7	Chr5: 24178191-24175473
OsCPK15	LOC_Os05g50810	1698	566	7	Chr5: 29144552-29140430

OsCPK16	LOC_Os05g39090	1644	548	6	Chr5: 22924421-22919309
OsCPK17	LOC_Os07g06740	1707	569	7	Chr7: 3293291-3288477
OsCPK18	LOC_Os07g22710	1539	513	11	Chr7: 12803568-12794960
OsCPK19	LOC_Os07g33110	1602	534	8	Chr7: 19773487-19769005
OsCPK20	LOC_Os07g38120	1653	551	7	Chr7: 22862582-22858595
OsCPK21	LOC_Os08g42750	1698	566	6	Chr8: 27059375-27054201
OsCPK22	LOC_Os09g33910	1734	578	7	Chr9: 20019391-20015571
OsCPK23	LOC_Os10g39420	1605	535	7	Chr10: 21027454-21032225
OsCPK24	LOC_Os11g07040	1542	514	7	Chr11: 3499974-3495218
OsCPK25	LOC_Os11g04170	1626	542	5	Chr11: 1691861-1694301
OsCPK26	LOC_Os12g03970	1626	542	5	Chr11: 1648300-1650655
OsCPK27	LOC_Os12g30150	1839	613	6	Chr12: 18098675-18101837
OsCPK28	LOC_Os12g07230	1581	527	6	Chr12: 3558400-3553396
OsCPK29	LOC_Os12g12860	1692	564	7	Chr12: 7112286-7116204
OsCPK30	LOC_Os07g44710	1785	595	10	Chr7: 26688827-26683038
Ostreococcus lucimarinus					
OlCPK3	e_gwEuk.14.119.1	1374	457	0	Chr_14: 206447 - 207820
OlCPK17	estExt_fgenesh1_pg.C_Chr_30268	1440	479	1	Chr_3: 566518 - 568296
OlCPK19	estExt_Genewise_ext.C_Chr_60025	1569	522	0	Chr_6: 693872 - 695665
Panicum virgatum					
PvCPK1-1	Pavirv00040420m.g	1845	614	5	sg0.contig53939: 2043 - 4569
PvCPK1-2	Pavirv00037785m.g	1821	606	7	sg0.contig00387: 1681 - 4763
PvCPK1-3	Pavirv00017178m.g	1857	618	6	sg0.contig66497: 647 - 3755
PvCPK1-4	Pavirv00031162m.g	1704	567	7	sg0.contig06363: 9485 - 14316
PvCPK1-5	Pavirv00060314m.g	1752	583	6	sg0.contig14982: 1633 - 4947
PvCPK1-6	Pavirv00010438m.g	1752	583	7	sg0.contig05298: 6492 - 10641
PvCPK1-7	Pavirv00048385m.g	954	317	4	sg0.contig158122: 1204 - 2706
PvCPK1-8	Pavirv00011826m.g	936	311	6	sg0.contig83410: 1500 - 3809
PvCPK3-1	Pavirv00016837m.g	1383	460	7	sg0.contig63324: 1120 - 4875
PvCPK3-2	Pavirv00014196m.g	1317	438	7	sg0.contig18449: 6397 - 9646
PvCPK3-3	Pavirv00030399m.g	1206	401	7	sg0.contig06530: 12005 - 15310
PvCPK3-4	Pavirv00015840m.g	1119	373	6	sg0.contig63410: 37 - 4374
PvCPK4-1	Pavirv00064150m.g	1548	515	6	sg0.contig22897: 2902 - 7301
PvCPK4-2	Pavirv00024283m.g	1548	515	6	sg0.contig02279: 13568 - 18293
PvCPK4-3	Pavirv00007698m.g	1539	512	7	sg0.contig46526: 1207 - 5223
PvCPK4-4	Pavirv00002804m.g	1431	476	6	sg0.contig34327: 1573 - 3844
PvCPK4-5	Pavirv00049634m.g	936	311	5	sg0.contig71937: 1664 - 3943
PvCPK5-1	Pavirv00003075m.g	1449	482	6	sg0.contig33946: 2230 - 7069
PvCPK5-2	Pavirv00044902m.g	1473	490	6	sg0.contig26274: 109 - 3674
PvCPK5-3	Pavirv00044833m.g	1470	489	6	sg0.contig25948: 3437 - 8134

PvCPK5-4	Pavirv00049417m.g	1470	489	6	sg0.contig72448: 45 - 4201
PvCPK5-5	Pavirv00002047m.g	1719	572	1	sg0.contig103230: 1428 - 3257
PvCPK5-6	Pavirv00059889m.g	1728	575	1	sg0.contig152377: 184 - 2023
PvCPK6	Pavirv00016031m.g	1629	542	8	sg0.contig61262: 452 - 5075
PvCPK7-1	Pavirv00069650m.g	1509	502	7	sg0.contig09681: 3 - 3607
PvCPK7-2	Pavirv00014425m.g	1047	348	6	sg0.contig40669: 667 - 3061
PvCPK7-3	Pavirv00016858m.g	1629	542	7	sg0.contig65224: 719 - 4097
PvCPK8-1	Pavirv00070851m.g	1626	541	7	sg0.contig33034: 2241 - 5686
PvCPK8-2	Pavirv00026354m.g	1596	531	7	sg0.contig24236: 4708 - 8423
PvCPK9-1	Pavirv00018537m.g	1596	531	8	sg0.contig50602: 396 - 4728
PvCPK9-2	Pavirv00063027m.g	1596	531	8	sg0.contig01879: 15562 - 21356
PvCPK9-3	Pavirv00044023m.g	1518	505	8	sg0.contig37659: 1821 - 5815
PvCPK13-1	Pavirv00065690m.g	1638	545	6	sg0.contig11695: 3779 - 8456
PvCPK13-2	Pavirv00068905m.g	1638	545	6	sg0.contig09717: 6567 - 11155
PvCPK13-3	Pavirv00021833m.g	1596	531	7	sg0.contig14663: 7534 - 10498
PvCPK16-1	Pavirv00029104m.g	1536	511	11	sg0.contig07945: 4960 - 13220
PvCPK16-2	Pavirv00048594m.g	1290	429	10	sg0.contig15920: 2142 - 6564
PvCPK17-1	Pavirv00035994m.g	1398	465	5	sg0.contig92839: 19 - 1913
PvCPK17-2	Pavirv00011855m.g	1398	465	5	sg0.contig84889: 26 - 1922
PvCPK17-3	Pavirv00014051m.g	1299	432	5	sg0.contig188016: 1 - 2155
PvCPK24	Pavirv00021668m.g	828	275	1	sg0.contig142916: 245 - 2318
PvCPK29-1	Pavirv00054378m.g	1620	539	7	sg0.contig21364: 1734 - 4309
PvCPK29-2	Pavirv00016737m.g	1194	397	6	sg0.contig64683: 2521 - 4883
PvCPK30-1	Pavirv00024932m.g	1719	572	3	sg0.contig36000: 1708 - 4888
PvCPK30-2	Pavirv00001784m.g	1734	577	4	sg0.contig10326: 1011 - 5071
PvCPK32-1	Pavirv00013424m.g	1053	350	2	sg0.contig18957: 93 - 1344
PvCPK32-2	Pavirv00011479m.g	1686	561	3	sg0.contig88557: 45 - 2191
PvCPK32-3	Pavirv00068592m.g	1743	580	6	sg0.contig100510: 27 - 2846
PvCPK32-4	Pavirv00018203m.g	1728	575	7	sg0.contig48384: 79 - 2968
PvCPK32-5	Pavirv00049858m.g	1599	532	7	sg0.contig67055: 1584 - 4815
PvCPK34-1	Pavirv00017949m.g	1563	520	6	sg0.contig47997: 2274 - 5150
PvCPK34-2	Pavirv00007044m.g	1563	520	6	sg0.contig47517: 2318 - 4986
PvCPK34-3	Pavirv00019274m.g	1395	464	6	sg0.contig47859: 125 - 2215
Phaseolus vulgaris					
PvuCPK1	Phvul.007G233900	1746	581	7	Chr07: 47402023 - 47409431
PvuCPK2	Phvul.007G266100	1749	582	7	Chr07: 50375268 - 50380318
PvuCPK3-1	Phvul.002G294500	1560	519	7	Chr02: 45785936 - 45794727
PvuCPK3-2	Phvul.002G161200	1509	502	7	Chr02: 30307579 - 30313970
PvuCPK4-1	Phvul.007G089200	1476	491	6	Chr07: 9046700 - 9051458
PvuCPK4-2	Phvul.007G089300	1677	558	6	Chr07: 9052511 - 9061441

PvuCPK8	Phvul.007G253300	1551	516	7	Chr07: 49171398 - 49176561
PvuCPK9-1	Phvul.006G043700	1389	462	7	Chr06: 15701662 - 15708144
PvuCPK9-2	Phvul.008G266600	1578	525	7	Chr08: 57769657 - 57774051
PvuCPK10	Phvul.002G087800	1653	550	6	Chr02: 13885894 - 13890809
PvuCPK11-1	Phvul.009G160100	1491	496	6	Chr09: 23183138 - 23189462
PvuCPK11-2	Phvul.002G279300	1518	505	6	Chr02: 44323845 - 44329216
PvuCPK13	Phvul.008G098400	1596	531	6	Chr08: 10383908 - 10392491
PvuCPK17-1	Phvul.008G201900	1566	521	7	Chr08: 51206612 - 51209875
PvuCPK17-2	Phvul.006G015300	1635	544	7	Chr06: 7353127 - 7356985
PvuCPK20	Phvul.007G265100	1749	582	6	Chr07: 50272848 - 50279301
PvuCPK21	Phvul.003G078400	1641	546	7	Chr03: 12300930 - 12305553
PvuCPK24	Phvul.011G055400	1620	539	7	Chr11: 4719324 - 4723394
PvuCPK25	Phvul.001G197700	1728	575	6	Chr01: 46377565 - 46381535
PvuCPK26	Phvul.008G292500	1689	562	8	Chr08: 59487940 - 59496832
PvuCPK28-1	Phvul.002G108700	1710	569	11	Chr02: 21995153 - 22001011
PvuCPK28-2	Phvul.003G261700	1584	527	11	Chr03: 48961700 - 48972324
PvuCPK29	Phvul.001G002900	1536	511	7	Chr01: 301611 - 304717
PvuCPK30	Phvul.003G194100	1614	537	6	Chr03: 40631819 - 40635469
PvuCPK32	Phvul.001G135300	1617	538	7	Chr01: 38040295 - 38044741
Physcomitrella patens					
PpCPK1-1	Pp1s49_208V6	1476	491	1	scaffold_49: 1635733 - 1639100
PpCPK1-2	Pp1s49_200V6	1476	491	0	scaffold_49: 1622173 - 1623648
PpCPK1-3	Pp1s205_14V6	1728	575	2	scaffold_205: 106030 - 110344
PpCPK1-4	Pp1s166_57V6	1653	550	3	scaffold_166: 383541 - 386920
PpCPK1-5	Pp1s138_79V6	1491	496	0	scaffold_138: 334316 - 336595
PpCPK1-6	Pp1s309_91V6	1485	494	1	scaffold_309: 470386 - 474005
PpCPK2-1	Pp1s187_88V6	1782	593	1	scaffold_187: 560003 - 564690
PpCPK2-2	Pp1s187_77V6	1479	492	1	scaffold_187: 496809 - 501148
PpCPK7-1	Pp1s232_44V6	1545	514	2	scaffold_232: 473870 - 477518
PpCPK7-2	Pp1s97_71V6	1575	524	0	scaffold_97: 544621 - 547269
PpCPK7-3	Pp1s2_191V6	1572	523	0	scaffold_2: 920544 - 922115
PpCPK7-4	Pp1s2_156V6	1572	523	0	scaffold_2: 920544 - 922115
PpCPK9	Pp1s143_92V6	1887	628	1	scaffold_143: 654636 - 658329
PpCPK13	Pp1s364_58V6	1584	527	0	scaffold_364: 311471 - 313054
PpCPK16-1	Pp1s199_57V6	1773	590	11	scaffold_199: 302843 - 307935
PpCPK16-2	Pp1s370_37V6	1650	549	11	scaffold_370: 198653 - 203685
PpCPK16-3	Pp1s83_172V6	1770	589	11	scaffold_83: 1216332 - 1220736
PpCPK16-4	Pp1s83_8V6	1968	655	12	scaffold_83: 71127 - 75911
PpCPK17-1	Pp1s96_216V6	1650	549	1	scaffold_96: 1274773 - 1277753
PpCPK17-2	Pp1s316_13V6	1638	545	0	scaffold_316: 150214 - 152724

PpCPK17-3	Pp1s325_31V6	1605	534	0	scaffold_325: 178530 - 180134
PpCPK17-4	Pp1s108_32V6	1737	578	1	scaffold_108: 181188 - 184809
PpCPK17-5	Pp1s108_25V6	1704	567	0	scaffold_108: 166096 - 168260
PpCPK30	Pp1s364_61V6	1587	528	0	scaffold_364: 352091 - 354113
Picea abies					
PaCPK3	MA_13110g0010	890	295	7	MA_13110:19..4943
PaCPK5	MA_115550g0010	1666	554	6	MA_115550:4858..14958
PaCPK6	MA_10429609g0010	938	311	6	MA_10429609:19..15254
PaCPK9	MA_98632g0020	1401	466	7	MA_98632:21890..33920
PaCPK13	MA_9458g0010	1656	551	7	MA_9458:19794..35545
PaCPK15	MA_3553g0010	1017	338	6	MA_3553:5489..7569
PaCPK17	MA_18543g0010	1602	533	7	MA_18543:25829..60996
PaCPK21	MA_10435930g0030	1113	370	2	MA_10435930:24068..25738
PaCPK29	MA_10435930g0050	939	312	7	MA_10435930:36785..39224
PaCPK33	MA_70277g0010	852	283	1	MA_70277:33622..34631
PaCPK34	MA_10437101g0010	1353	450	9	MA_10437101:6284..20753
Populus trichocarpa					
PtCPK1	Potri.010G244800	1770	589	7	Chr10: 21987202 - 21991958
PtCPK2	Potri.008G014700	1740	579	6	Chr08: 803852 - 808486
PtCPK3-1	Potri.003G134000	1572	523	9	Chr03: 15333647 - 15338743
PtCPK3-2	Potri.001G097400	1548	515	8	Chr01: 7653953 - 7659297
PtCPK6-1	Potri.009G168600	1677	558	8	Chr09: 12817517 - 12823086
PtCPK6-2	Potri.004G207300	1683	560	9	Chr04: 21612692 - 21618453
PtCPK7	Potri.001G257100	1605	534	7	Chr01: 26667674 - 26672003
PtCPK8	Potri.009G052700	1602	533	9	Chr09: 5689673 - 5694937
PtCPK9	Potri.004G015500	1599	532	7	Chr04: 994606 - 1000107
PtCPK11	Potri.019G083200	1560	519	6	Chr19: 11623968 - 11628657
PtCPK12	Potri.T012800	1512	503	6	scaffold_25: 202598 - 207090
PtCPK13-1	Potri.006G101300	1737	578	7	Chr06: 7795561 - 7802392
PtCPK13-2	Potri.016G117200	1587	528	6	Chr16: 12234530 - 12241141
PtCPK17	Potri.009G069200	1578	525	7	Chr09: 6843123 - 6845510
PtCPK20-1	Potri.006G199400	1797	598	6	Chr06: 21455094 - 21460753
PtCPK20-2	Potri.016G065700	1800	599	6	Chr16: 4631026 - 4637716
PtCPK24	Potri.007G127000	1605	534	7	Chr07: 14321126 - 14324857
PtCPK25	Potri.016G066700	1842	613	7	Chr16: 4741028 - 4745429
PtCPK28-1	Potri.007G057600	1674	557	11	Chr07: 6095917 - 6102292
PtCPK28-2	Potri.005G113600	1671	556	11	Chr05: 8726353 - 8732612
PtCPK29-1	Potri.002G017000	1629	542	7	Chr02: 986409 - 990224
PtCPK29-2	Potri.005G245000	1542	513	7	Chr05: 24953832 - 24958655
PtCPK30-1	Potri.015G066200	1689	562	6	Chr15: 9089007 - 9093044

PtCPK30-2	Potri.012G071700	1668	555	6	Chr12: 9696439 - 9701344
PtCPK32-1	Potri.006G052900	1587	528	7	Chr06: 3811387 - 3815648
PtCPK32-2	Potri.016G054600	1599	532	8	Chr16: 3549562 - 3554722
PtCPK33	Potri.011G003400	1599	532	7	Chr11: 245219 - 251121
PtCPK34	Potri.001G274700	1518	505	8	Chr01: 28118942 - 28122193
Prunus perscia					
PpeCPK1	ppa003542m.g	1704	567	6	scaffold_4: 21577698 - 21584701
PpeCPK2	ppa002734m.g	1920	639	6	scaffold_7: 12823736 - 12826713
PpeCPK3	ppa004162m.g	1581	526	7	scaffold_5: 11503594 - 11508680
PpeCPK8-1	ppa006164m.g	1278	425	7	scaffold_4: 18649008 - 18652908
PpeCPK8-2	ppa004069m.g	1596	531	7	scaffold_7: 9889985 - 9894870
PpeCPK9	ppa003830m.g	1638	545	7	scaffold_4: 13342829 - 13347541
PpeCPK10	ppa003795m.g	1647	548	7	scaffold_5: 15354135 - 15358116
PpeCPK11	ppa004665m.g	1494	497	6	scaffold_8: 7108411 - 7113030
PpeCPK12	ppa004580m.g	1509	502	0	scaffold_3: 1711490 - 1713101
PpeCPK13	ppa004141m.g	1584	527	6	scaffold_6: 24253001 - 24258038
PpeCPK17	ppa026653m.g	1605	534	7	scaffold_4: 22960834 - 22963576
PpeCPK20	ppa003092m.g	1818	605	6	scaffold_7: 12724445 - 12729905
PpeCPK21	ppa006748m.g	1194	397	7	scaffold_4: 1002107 - 1005710
PpeCPK24	ppa021005m.g	1578	526	7	scaffold_1: 16622683 - 16625601
PpeCPK26	ppa003459m.g	1722	573	7	scaffold_8: 17381330 - 17385040
PpeCPK28	ppa003676m.g	1674	557	11	scaffold_7: 17542990 - 17547513
PpeCPK29	ppa004027m.g	1605	534	7	scaffold_1: 32253459 - 32256660
Ricinus communis					
RcCPK1	29852.t000019	1737	578	6	29852: 748819 - 754396
RcCPK3	30190.t000579	1587	528	8	30190: 3182411 - 3187365
RcCPK6	30100.t000014	1686	561	6	30100: 635436 - 641118
RcCPK8	30169.t000219	1611	536	7	30169: 1209760 - 1213471
RcCPK11	29728.t000018	1494	497	6	29728: 126364 - 131585
RcCPK13	28308.t000002	1578	525	6	28308: 38002 - 44802
RcCPK17	30142.t000025	1611	536	7	30142: 206467 - 209308
RcCPK20	29333.t000051	1755	584	6	29333: 380788 - 386245
RcCPK21	29842.t000094	1656	551	7	29842: 470020 - 473807
RcCPK24	29896.t000003	1602	533	7	29896: 35082 - 37969
RcCPK25	29333.t000041	1926	641	6	29333: 292448 - 295456
RcCPK29	30170.t000694	1590	529	7	30170: 3717810 - 3721220
RcCPK30	30147.t000328	1650	549	6	30147: 4261059 - 4265373
RcCPK32	29761.t000015	1590	529	7	29761: 124474 - 128639
RcCPK33	27777.t000008	1602	533	7	27777: 46786 - 50324
Selaginella moellendorffii					

SmCPK1	99178	1485	494	6	scaffold_21: 394054 - 395977
SmCPK2	165073	1494	497	6	scaffold_1: 4925614 - 4928206
SmCPK3	118877	1482	493	7	scaffold_59: 1150368 - 1152640
SmCPK7	105846	1620	539	1	scaffold_32: 1586745 - 1588476
SmCPK13	105020	1596	531	0	scaffold_30: 543075 - 544670
SmCPK16	92726	1515	504	10	scaffold_13: 597405 - 599565
SmCPK17-1	96034	1491	496	7	scaffold_17: 1739027 - 1740942
SmCPK17-2	152133	1461	486	11	scaffold_35: 942821 - 946212
SmCPK28	164119	1632	543	12	scaffold_0: 4195613 - 4198041
Setaria italica					
SiCPK1-1	Si034743m.g	1845	614	6	scaffold_9: 3622843 - 3626164
SiCPK1-2	Si034852m.g	1749	582	6	scaffold_9: 3605681 - 3608792
SiCPK1-3	Si029343m.g	1722	573	7	scaffold_2: 3226075 - 3230166
SiCPK2	Si021787m.g	1542	513	6	scaffold_3: 14972218 - 14975951
SiCPK3-1	Si001025m.g	1569	522	7	scaffold_5: 29354916 - 29359305
SiCPK3-2	Si021672m.g	1641	546	7	scaffold_3: 8776034 - 8779649
SiCPK4	Si009864m.g	1548	515	6	scaffold_7: 33248289 - 33252464
SiCPK5-3	Si009748m.g	1671	556	8	scaffold_7: 28338562 - 28343738
SiCPK5-1	Si016803m.g	1680	559	6	scaffold_1: 34831128 - 34835537
SiCPK5-2	Si016805m.g	1677	558	0	scaffold_1: 42005655 - 42007426
SiCPK6-1	Si035094m.g	1608	535	6	scaffold_9: 40133379 - 40137419
SiCPK6-2	Si040704m.g	1584	527	6	scaffold_9: 57206117 - 57209719
SiCPK7-1	Si035115m.g	1596	531	7	scaffold_9: 2438683 - 2442448
SiCPK7-2	Si021615m.g	1707	568	7	scaffold_3: 6126351 - 6129197
SiCPK8	Si029431m.g	1626	541	7	scaffold_2: 43623945 - 43627599
SiCPK9	Si029460m.g	1596	531	8	scaffold_2: 41740491 - 41744846
SiCPK11	Si026386m.g	1308	435	6	scaffold_8: 4338508 - 4341101
SiCPK13-1	Si000940m.g	1626	541	6	scaffold_5: 40607909 - 40614620
SiCPK13-2	Si021667m.g	1647	548	7	scaffold_3: 17042942 - 17048442
SiCPK13-3	Si015574m.g	1671	556	1	scaffold_6: 34338578 - 34340381
SiCPK13-4	Si029399m.g	1662	553	7	scaffold_2: 37157363 - 37160378
SiCPK16	Si016899m.g	1578	525	11	scaffold_1: 8840815 - 8847062
SiCPK20	Si025125m.g	1881	626	5	scaffold_3: 46584357 - 46586884
SiCPK29	Si009791m.g	1605	534	7	scaffold_7: 27056637 - 27059382
SiCPK30	Si034847m.g	1752	583	2	scaffold_9: 8599271 - 8602735
SiCPK34-1	Si024765m.g	1590	529	6	scaffold_3: 14854877 - 14857100
SiCPK34-2	Si003781m.g	1134	377	6	scaffold_5: 39548186 - 39549956
Solanum lycopersicum					
SlCPK1-1	Solyc04g009800	1746	581	6	SL2.40ch04: 3108503 - 3114299
SlCPK1-2	Solyc11g006370	1737	578	6	ch11: 1080169 - 1085490

SlCPK2-1	Solyc10g081740	1500	499	7	ch10: 62082038 - 62086600
SlCPK2-2	Solyc01g006840	1797	598	6	ch01: 1410577 - 1414084
SlCPK3	Solyc08g008170	1551	516	7	ch08: 2637882 - 2642478
SlCPK4-1	Solyc06g065380	1524	507	7	ch06: 37191712 - 37195833
SlCPK4-2	Solyc11g018610	1518	505	6	ch11: 8769972 - 8773835
SlCPK6-1	Solyc10g074570	1674	557	6	ch10: 57454225 - 57458658
SlCPK6-2	Solyc01g112250	1749	582	7	ch01: 1328459 - 1332580
SlCPK7-1	Solyc10g079130	1578	525	7	ch10: 60064941 - 60068542
SlCPK7-2	Solyc11g065660	1602	533	7	ch11: 48274194 - 48279107
SlCPK9	Solyc07g064610	1566	521	8	ch07: 63903803 - 63909109
SlCPK10	Solyc03g113390	1617	538	6	ch03: 57593080 - 57598567
SlCPK11-1	Solyc04g049160	1527	508	6	ch04: 39080746 - 39092319
SlCPK11-2	Solyc05g056570	1512	503	6	ch05: 64966125 - 64971506
SlCPK13	Solyc09g005550	1590	529	6	ch09: 369215 - 377428
SlCPK16	Solyc02g083850	1713	570	11	ch02: 41690032 - 41695031
SlCPK17-1	Solyc11g064900	1590	529	7	ch11: 47269846 - 47276122
SlCPK17-2	Solyc01g008740	1626	541	7	ch01: 2776811 - 2782126
SlCPK20-1	Solyc10g076900	1506	501	7	ch10: 59195105 - 59198922
SlCPK20-2	Solyc01g006730	1749	582	7	ch01: 1328459 - 1332580
SlCPK20-3	Solyc10g081640	1740	579	6	ch10: 62001043 - 62005206
SlCPK21	Solyc03g031670	1662	553	8	ch03: 8397378 - 8406044
SlCPK24	Solyc06g073350	1611	536	8	ch06: 41589654 - 41593571
SlCPK28	Solyc03g033540	1698	565	11	ch03: 9356208 - 9363698
SlCPK29	Solyc04g081910	1566	521	7	ch04: 63365446 - 63370378
SlCPK32	Solyc01g008440	1602	533	7	ch01: 2523675 - 2529463
SlCPK34	Solyc12g099790	1608	535	8	ch12: 65130565 - 65133955
Solanum tuberosum					
StCPK1-1	PGSC0003DMG400000994	1737	578	6	Chr11: 56449 - 62816
StCPK1-2	PGSC0003DMG400028229	1824	607	6	Chr10: 49040605 - 49045872
StCPK2	PGSC0003DMG400027527	1548	515	7	Chr1: 80701293 - 80705437
StCPK3	PGSC0003DMG400005829	1554	517	7	Chr8: 5185401 - 5190332
StCPK4	PGSC0003DMG400026077	1506	501	6	Chr6: 44042067 - 44046412
StCPK6	PGSC0003DMG400016820	1704	567	6	Chr10: 39812377 - 39816690
StCPK8	PGSC0003DMG400010704	1602	533	7	Chr1: 3760775 - 3767680
StCPK9-1	PGSC0003DMG400027877	1530	509	8	Chr12: 7995763 - 8002907
StCPK9-2	PGSC0003DMG400022318	1566	521	7	Chr7: 53299362 - 53305161
StCPK11-1	PGSC0003DMG400023440	1512	503	6	Chr5: 61019583 - 61025389
StCPK11-2	PGSC0003DMG400000890	1518	505	6	Chr11: 10547948 - 10552423
StCPK16-1	PGSC0003DMG400022562	1707	568	11	Chr3: 6059932 - 6067758
StCPK16-2	PGSC0003DMG400003564	1695	564	11	Chr2: 61403984 - 61410033

StCPK17-1	PGSC0003DMG400004646	1608	535	8	Chr12: 67004446 - 67008742
StCPK17-2	PGSC0003DMG400009451	1575	524	7	Chr11: 33714734 - 33720691
StCPK20-1	PGSC0003DMG401007209	1635	544	6	Chr10: 43247336 - 43252023
StCPK20-2	PGSC0003DMG401028133	1917	638	7	Chr10: 49123120 - 49127708
StCPK20-3	PGSC0003DMG400021342	1749	582	7	Chr1: 2082018 - 2087027
StCPK24	PGSC0003DMG400026908	1611	536	8	Chr6: 47417118 - 47421989
StCPK29	PGSC0003DMG400009883	1623	540	7	Chr4: 63318768 - 63324389
StCPK32	PGSC0003DMG400008149	1575	524	7	Chr10: 44307063 - 44311558
Sorghum bicolor					
SbCPK1-1	Sb01g005780	1854	617	6	chromosome_1: 4771888 - 4775943
SbCPK1-2	Sb08g014910	1938	645	4	chromosome_8: 39069692 - 39072103
SbCPK2-1	Sb01g005750	1758	585	6	chromosome_1: 4722615 - 4726021
SbCPK2-2	Sb02g003500	1746	581	7	chromosome_2: 3949285 - 3954691
SbCPK3-1	Sb03g028340	1578	525	7	chromosome_3: 56124118 - 56128760
SbCPK3-2	Sb09g029950	1626	541	7	chromosome_9: 58613330 - 58617248
SbCPK4	Sb05g004610	1548	515	7	chromosome_5: 5837360 - 5841218
SbCPK5	Sb04g031570	1473	490	6	chromosome_4: 61527139 - 61532455
SbCPK6-1	Sb06g026530	1668	555	6	chromosome_6: 55593138 - 55597448
SbCPK6-2	Sb01g048570	1635	544	7	chromosome_1: 71541078 - 71545901
SbCPK7	Sb08g007660	1710	569	7	chromosome_8: 14078898 - 14082308
SbCPK8-1	Sb02g036730	1632	543	7	chromosome_2: 71116574 - 71119856
SbCPK8-2	Sb01g004150	1599	532	7	chromosome_1: 3344432 - 3348438
SbCPK9	Sb02g034640	1596	531	8	chromosome_2: 69150066 - 69154180
SbCPK11	Sb08g004510	1548	515	6	chromosome_8: 5413386 - 5417550
SbCPK13-1	Sb09g022960	1632	543	6	chromosome_9: 52586040 - 52590913
SbCPK13-2	Sb03g038870	1638	545	6	chromosome_3: 66661067 - 66670497
SbCPK13-3	Sb07g025560	1737	578	3	chromosome_7: 60647763 - 60649750
SbCPK16	Sb02g009790	1545	514	11	chromosome_2: 14062232 - 14076032
SbCPK17-1	Sb09g024100	1584	527	4	chromosome_9: 53675914 - 53677907
SbCPK17-2	Sb05g002110	1617	538	5	chromosome_5: 2284151 - 2286387
SbCPK17-3	Sb08g001380	1725	574	6	chromosome_8: 1344647 - 1347393
SbCPK26	Sb04g038450	1743	580	1	chromosome_4: 67841767 - 67843614
SbCPK28	Sb04g002220	1578	525	11	chromosome_4: 2023811 - 2030267
SbCPK29	Sb06g025220	1602	533	7	chromosome_6: 54190773 - 54193170
SbCPK30	Sb01g011630	1761	586	4	chromosome_1: 10484678 - 10488479
SbCPK32	Sb03g043700	1674	557	7	chromosome_3: 70965233 - 70968784
SbCPK34	Sb03g037570	1389	462	6	chromosome_3: 65485324 - 65487344
Thellungiella halophila					
ThCPK1	Thhalv10012990m.g	1842	613	6	scaffold_2: 1359841 - 1363099
ThCPK2	Thhalv10020268m.g	1938	645	6	scaffold_13: 6030608 - 6033377

ThCPK3	Thhalv10024899m.g	1584	527	8	scaffold_1: 7029435 - 7032336
ThCPK4	Thhalv10028598m.g	1506	501	6	scaffold_3: 9261644 - 9264454
ThCPK5	Thhalv10024803m.g	1692	563	6	scaffold_1: 1964698 - 1967485
ThCPK6	Thhalv10022628m.g	1662	553	7	scaffold_11: 2132434 - 2135710
ThCPK7	Thhalv10013170m.g	1647	548	7	scaffold_2: 4122132 - 4125005
ThCPK8	Thhalv10013210m.g	1605	534	8	scaffold_2: 6691217 - 6694648
ThCPK9	Thhalv10020475m.g	1614	537	8	scaffold_13: 2066531 - 2069704
ThCPK10	Thhalv10007293m.g	1638	545	6	scaffold_5: 8997208 - 9000754
ThCPK11	Thhalv10001816m.g	1494	497	6	scaffold_23: 909674 - 912506
ThCPK12	Thhalv10004083m.g	1473	490	5	scaffold_6: 2914437 - 2916988
ThCPK13	Thhalv10010284m.g	1587	528	6	scaffold_16: 1790222 - 1793674
ThCPK14	Thhalv10016499m.g	1593	530	7	scaffold_10: 11400965 - 11403460
ThCPK15	Thhalv10027200m.g	1005	334	7	scaffold_1: 7778892 - 7781197
ThCPK16	Thhalv10022615m.g	1740	579	11	scaffold_11: 1177603 - 1180461
ThCPK17	Thhalv10013236m.g	1587	528	7	scaffold_2: 4006811 - 4009098
ThCPK18	Thhalv10027243m.g	1560	519	11	scaffold_1: 1695829 - 1698563
ThCPK19	Thhalv10023804m.g	1662	553	8	scaffold_8: 2374744 - 2377263
ThCPK20	Thhalv10017647m.g	1761	586	6	scaffold_10: 9946368 - 9948721
ThCPK21	Thhalv10028567m.g	1608	535	7	scaffold_3: 2302485 - 2305894
ThCPK22	Thhalv10028617m.g	1458	485	7	scaffold_3: 2312498 - 2315067
ThCPK23	Thhalv10026767m.g	1146	381	8	scaffold_1: 7795638 - 7800116
ThCPK24	Thhalv10016430m.g	1746	581	6	scaffold_10: 6442661 - 6445221
ThCPK26	Thhalv10025058m.g	1452	483	8	scaffold_1: 49782 - 52195
ThCPK28	Thhalv10003969m.g	1605	534	11	scaffold_6: 80941 - 84259
ThCPK29	Thhalv10018359m.g	1668	555	7	scaffold_9: 2009835 - 2012741
ThCPK30	Thhalv10018366m.g	1641	546	6	scaffold_9: 2585413 - 2588670
ThCPK31	Thhalv10028618m.g	1458	485	7	scaffold_3: 2327372 - 2330514
ThCPK32	Thhalv10005884m.g	1605	534	7	scaffold_19: 2110292 - 2113444
ThCPK34	Thhalv10013240m.g	1584	527	6	scaffold_2: 6651471 - 6653603
Theobroma cacao					
TcCPK1	Thecc1EG044380	1752	583	7	scaffold_10r: 16353607 - 16361005
TcCPK2	Thecc1EG023099	1824	607	6	scaffold_5: 10392248 - 10397467
TcCPK3	Thecc1EG016367	1596	531	8	scaffold_3: 32575810 - 32580942
TcCPK4	Thecc1EG001677	1485	494	7	scaffold_1: 8675974 - 8684419
TcCPK6	Thecc1EG007580	1689	562	8	scaffold_2: 7761495 - 7767904
TcCPK7	Thecc1EG024724	1602	533	7	scaffold_5: 28095829 - 28100863
TcCPK8	Thecc1EG040717	1599	532	11	scaffold_9: 32853676 - 32862554
TcCPK11	Thecc1EG036367	1701	566	7	scaffold_8: 18433941 - 18440041
TcCPK13	Thecc1EG021684	1665	554	6	scaffold_5: 775525 - 781379
TcCPK21	Thecc1EG030220	1617	538	7	scaffold_6: 26252582 - 26257221

TcCPK24	Thecc1EG020113	1614	537	7	scaffold_4: 26719066 - 26722572
TcCPK28	Thecc1EG000206	1848	615	11	scaffold_1: 801889 - 807658
TcCPK29-1	Thecc1EG034291	1551	516	8	scaffold_8: 2315034 - 2321279
TcCPK29-2	Thecc1EG034289	1584	527	7	scaffold_8: 2306516 - 2311310
TcCPK30	Thecc1EG013784	1653	550	6	scaffold_3: 17154186 - 17160341
TcCPK33	Thecc1EG030876	1611	536	7	scaffold_7: 1763829 - 1768646
TcCPK34	Thecc1EG040893	1608	535	7	scaffold_9: 34164588 - 34168024
Vitis venifera					
VvCPK1	GSVIVG01001931001	1029	342	6	Chr13: 14945177 - 14952111
VvCPK2	GSVIVG01022606001	1695	564	7	Chr8: 5882896 - 5886842
VvCPK3	GSVIVG01019446001	1200	399	7	Chr2: 734377 - 743551
VvCPK4	GSVIVG01034489001	1041	346	6	Chr18: 20503034 - 20524687
VvCPK6	GSVIVG01023866001	1392	463	9	Chr3: 2890432 - 2895797
VvCPK8	GSVIVG01025249001	1557	518	7	Chr6: 2714924 - 2721367
VvCPK9	GSVIVG01037652001	996	331	7	Chr19: 6574167 - 6579250
VvCPK10	GSVIVG01008077001	1218	405	6	Chr17: 6012605 - 6021669
VvCPK12	GSVIVG01000238001	1044	347	6	Chr7: 20409585 - 20417391
VvCPK13	GSVIVG01011167001	1143	380	8	Chr8: 7623465 - 7641697
VvCPK17	GSVIVG01037295001	1494	497	8	Chr6: 16322877 - 16325979
VvCPK20	GSVIVG01022524001	1659	552	7	Chr8: 4329896 - 4341074
VvCPK21	GSVIVG01012730001	1545	514	8	Chr10: 1067226 - 1075577
VvCPK24	GSVIVG01010743001	1731	576	8	Chr5: 22039047 - 22055956
VvCPK28	GSVIVG01018778001	1677	558	11	Chr4: 20005136 - 20011413
VvCPK29	GSVIVG01008749001	1572	523	7	Chr18: 5266543 - 5272067
VvCPK32	GSVIVG01033306001	1191	369	7	Chr8: 21643456 - 21648959
Volvox carteri					
VcCPK4	Vocar20014761m.g	2919	972	13	scaffold_28: 546339 - 557503
VcCPK12	Vocar20008501m.g	1524	507	9	scaffold_1: 2436838 - 2441117
VcCPK15	Vocar20001731m.g	1590	529	3	scaffold_22: 488110 - 490656
VcCPK17	Vocar20000362m.g	1455	484	6	scaffold_17: 296545 - 302538
VcCPK20	Vocar20014333m.g	1956	651	13	scaffold_11: 752838 - 758649
VcCPK34	Vocar20005450m.g	1956	651	13	scaffold_11: 752838 - 758649
Zea mays					
ZmCPK1-1	GRMZM2G028926_T01	1827	608	6	Chr1: 285974147-285978219
ZmCPK1-2	GRMZM2G121228_T01	1743	580	7	Chr1: 286121446-286125765
ZmCPK1-3	GRMZM2G027351_T01	1755	584	7	Chr5: 4651173-4655150
ZmCPK1-4	GRMZM2G353957_T01	1941	646	4	Chr3: 103172508-103175313
ZmCPK1-5	GRMZM2G117796_T01	1788	595	10	Chr9: 127506454-127524007
ZmCPK2	GRMZM2G320506_T01	1863	620	6	Chr5: 4670108-4673481
ZmCPK3-1	GRMZM2G058305_T01	1620	539	7	Chr8: 75601621-75605633

ZmCPK3-2	GRMZM2G025387_T01	1593	530	8	Chr8: 145082985-145087859
ZmCPK3-3	GRMZM5G856738_T02	909	302	6	Chr3: 217700066-217702818
ZmCPK4-1	GRMZM2G347226_T01	1548	515	7	Chr10: 10197438-10201868
ZmCPK4-2	GRMZM2G035843_T01	1527	508	7	Chr4: 204664489-204668662
ZmCPK4-3	GRMZM2G463464_T01	1548	515	7	Chr3: 140852452-140857698
ZmCPK4-4	GRMZM2G047486_T01	1533	510	8	Chr2: 136178040-136182277
ZmCPK5-1	GRMZM2G321239_T01	1671	556	6	Chr10: 134917888-134922506
ZmCPK5-2	GRMZM2G314396_T01	1644	547	6	Chr2: 14534118-14542446
ZmCPK5-3	GRMZM2G347047_T01	1467	488	1	Chr4: 168736714-168738634
ZmCPK5-4	GRMZM2G040743_T01	1623	540	7	Chr1: 84293878-84298816
ZmCPK6-1	GRMZM2G081310_T01	1689	562	6	Chr4: 156993018-156999006
ZmCPK6-2	GRMZM2G032852_T02	1635	544	7	Chr1: 6193683-6198902
ZmCPK7	GRMZM2G097533_T01	1317	438	6	Chr3: 97780311-97783252
ZmCPK8-1	AC233871.1_FGT003	1620	539	7	Chr6: 1800002-1803000
ZmCPK8-2	GRMZM2G104125_T01	1608	535	7	Chr1: 290930529-290935131
ZmCPK8-3	GRMZM5G839017_T02	1503	500	10	Chr1: 179712399-179718770
ZmCPK8-4	GRMZM5G874665_T02	1467	488	10	Chr1: 20038692-20044821
ZmCPK8-5	GRMZM2G006404_T01	1068	355	6	Chr1: 54331306-54334007
ZmCPK8-6	GRMZM2G047479_T01	1068	355	6	Chr8: 153374915-153377610
ZmCPK9-1	GRMZM2G154489_T01	1596	531	8	Chr7: 152948218-152952482
ZmCPK9-2	GRMZM2G168706_T01	1596	531	8	Chr2: 202956904-202961091
ZmCPK13-1	GRMZM2G311220_T01	1611	536	6	Chr8: 169657383-169664344
ZmCPK13-2	GRMZM2G088361_T01	1623	540	6	Chr6: 152463328-152467959
ZmCPK13-3	GRMZM2G030673_T01	1626	541	6	Chr8: 117896321-117901322
ZmCPK16-1	GRMZM2G053868_T01	1569	522	11	Chr4: 13575055-13582169
ZmCPK16-2	GRMZM2G157068_T01	1569	522	11	Chr5: 73215830-73222945
ZmCPK17-1	GRMZM2G340224_T01	1842	613	7	Chr8: 120172631-120175620
ZmCPK17-2	GRMZM2G365815_T01	1659	552	6	Chr2: 148506696-148509137
ZmCPK17-3	AC203294.3_FGT001	1395	464	5	Chr8: 171275246-171277488
ZmCPK19	GRMZM2G028086_T01	1620	539	7	Chr7: 158740859-158744497
ZmCPK20	GRMZM2G012326_T01	2058	685	9	Chr2: 234205930-234212192
ZmCPK24	GRMZM2G332660_T01	1707	568	4	Chr4: 194112157-194114320
ZmCPK28	GRMZM2G365035_T01	1539	512	11	Chr2: 157290493-157298357
ZmCPK29	GRMZM2G112057_T01	1620	539	7	Chr10: 138210220-138213031
ZmCPK30	GRMZM2G076634_T01	972	323	3	Chr5: 13273540-13275228
ZmCPK32-1	AC210013.4_FGT014	1617	538	7	Chr5: 3408485-3412162
ZmCPK32-2	GRMZM2G099425_T01	1620	539	7	Chr2: 205927101-205930764
ZmCPK32-3	GRMZM2G158721_T01	1971	656	6	Chr2: 193207270-193210864
ZmCPK34-1	GRMZM2G167276_T01	1533	510	6	Chr3: 187261337-187263649
ZmCPK34-2	GRMZM2G472311_T01	1746	581	5	Chr4: 186592295-186594498
