# Supplementary material for: Genome-Wide Identification of Calcium Dependent Protein Kinase Gene Family in Plant Lineage Shows Presence of Novel D-x-D and D-E-L Motifs in EF-Hand Domain
Source: Front Plant Sci. 2015 Dec 24;6:1146. doi: 10.3389/fpls.2015.01146 (PMC4690006; doi:10.3389/fpls.2015.01146)
Supplement: Supplementary file 2 [file Table2.PDF]

Supplementary Table 2

The forward and reverse primer sequences of selected *Brassica rapa* CPK genes.

Gene Name	Forward Primer	Reverse Primer
BrCPK1	GGTGATACCGCAAGTGAAGCTGC	CCGGTTTTGGACTCTCAGGCTGA
BrCPK2	ACACCAGACGAGCTTCAACAGGC	TCCCTTCTGCATCATCGCCACAA
BrCPK3-1	CCTCCTCCTCCTCTTCCTCCGAC	CATCGGCTTGCCTAGGATCCGAC
BrCPK4	CCTTCAAGCAGCGTCCTCCCTTA	GCGCCACACATCCTCGTAATCCT
BrCPK5	AGCTCACAACAAGGATCCAGCTCT	GTGTAGAGGTCGCGGATGTTCGG
BrCPK6-1	GATGAGCTGCAACAATCGTGCGT	TTGTTCGTCTCCCAACACCAGCA
BrCPK7	GTTGATACTGACAAGGACGGGCG	TCCCTTGAATACTGCCGTGACGC
BrCPK8-1	GCAGTGAGGAGGTTATTGCAGCC	GTACTGCCTCGACGCTTTCCTCC
BrCPK12	CAGCAGGCTTTGAAGGAGTTTGGT	TGCCAATCCCTCCACTGCCATTT
BrCPK20	GAGCCTCGAGAGCAACAAGGACG	AGGAGGTGGTGTGCTTGGAGTTG
BrCPK28-1	TGGGTGTGTGTTTCTCCGCCATT	TGTTGGTGGTGGTGGTAGATGGC
