# Supplementary material for: Genome-Wide Identification of Calcium Dependent Protein Kinase Gene Family in Plant Lineage Shows Presence of Novel D-x-D and D-E-L Motifs in EF-Hand Domain
Source: Front Plant Sci. 2015 Dec 24;6:1146. doi: 10.3389/fpls.2015.01146 (PMC4690006; doi:10.3389/fpls.2015.01146)
Supplement: Supplementary file 3 [file Table3.PDF]

Supplementary Table 3

Putative palmitoylation site, molecular weight in kilo Dalton (kDa) and probable isoelectric point of different CPK genes.

Gene Name	Locus ID	Palmitoylation site	Mol. Weight (kDa)	pI
Aquilegia coerulea				
AcCPK2	Aquca_009_00629	***MGNTCVAPTASK	64.359	5.91
AcCPK3	Aquca_013_00210	****MGNCNGLPSDT	59.794	6.19
AcCPK7-1	Aquca_014_00772	****MGNCCASPASS	60.372	7.15
AcCPK7-2	Aquca_009_00303	****MGNCCGTPQVS	60.082	6.68
AcCPK11	Aquca_014_00140	QFGTTFLCTEKS	58.715	5.68
AcCPK13	Aquca_004_00375	****MGNCCRSPASV	60.91	6.25
AcCPK20-1	Aquca_004_00556	***MGNTCVGPSLGQ	67.128	5.59
AcCPK20-2	Aquca_009_00644	QFGTTFYCFEKATGK	106.94	4.94
AcCPK26-1	Aquca_015_00273	***MGNTCRRGSFGRK	62.261	5.89
AcCPK26-2	Aquca_007_00944	MGNSCRGSFRGK	64.578	6.17
AcCPK28-1	Aquca_005_00352	****MGICFSTSNTK	64.956	8.38
AcCPK28-2	Aquca_007_00052	****MGLCLSTTSNK	61.127	8.93
AcCPK29	Aquca_111_00023	****MGICFTRTHEI	59.505	6.21
AcCPK30	Aquca_002_00453	****MGNCCACVSIF	62.304	6.81
AcCPK33	Aquca_005_00072	****MGVCFSKKHGS	61.425	6.89
AcCPK34	Aquca_004_00793	****MGNCCSHGAPA	58.984	6.03
Arabidopsis thaliana				
AtCPK1	At5g04870	****MGNTCVGPSRNG	68.253	5.51
AtCPK2	At3g10660	***MGNACVGPNISG	72.253	5.42
AtCPK3	At4g23650	ETKQQVACKSIPTRR	59.336	6.37
AtCPK4	At4g09570	QFGTTYLLCTEKSSSA	56.416	5.38
AtCPK5	At4g35310	***MGNSCRGSFKDK	62.127	5.34
AtCPK6	At2g17290	***MGNSCRGSFKDK	61.111	5.67
AtCPK7	At5g12480	****MGNCCGNPSSA	60.309	6.23
AtCPK8	At5g19450	****MGNCCASPGSE	59.940	6.36
AtCPK9	At3g20410	****MGNCFAKNHGL	60.362	6.18
AtCPK10	At1g18890	****MGNCNACVRPD	61.459	6.62

AtCPK11	At1g35670	QFGTTYLCTEKSTSA	55.915	5.43
AtCPK12	At5g23580	QFGTTFLCTHKQTGQ	55.379	5.41
AtCPK13	At3g51850	****MGNCCRSPAAV	59.375	6.68
AtCPK14	At2g41860	****MGNCCGTAGSL	60.053	7.19
AtCPK15	At4g21940	*****MGCFSSKHRN	62.575	6.19
AtCPK16	At2g17890	****MGLCFSSAAKS	64.753	9.33
AtCPK17	At5g12180	****MGNCCSHGRDS	58.484	6.06
AtCPK18	At4g36070	****MGLCFSSPKAT	60.213	8.57
AtCPK19	At1g61950	*****MGCLCINLKK	62.948	7.02
AtCPK20	At2g38910	***MGNTCVGPNLNP	64.720	5.57
AtCPK21	At4g04720	*****MGCFSSKHRK	59.894	6.66
AtCPK22	At4g04710	****MGNCCGSKPLT	64.674	5.53
AtCPK23	At4g04740	*****MGCFSSKHRK	58.653	6.54
AtCPK24	At2g31500	****MGSCVSSPLKG	66.243	7.38
AtCPK25	At2g35890	***MGNVCVHMVNNC	58.851	5.86
AtCPK26	At4g38230	NLIRGMLCSRPSERL	38.204	5.04
AtCPK27	At4g04700	****MGCFSSKELQ	54.898	5.15
AtCPK28	At5g66210	****MGVCFSAIRVT	58.971	8.41
AtCPK29	At1g76040	****MFECILAKTLV	37.161	5.45
AtCPK30	At1g74740	****MGNCIACVKFD	61.404	6.73
AtCPK31	At4g04695	*****MGCYSSKNLK	54.696	6.38
AtCPK32	At3g57530	****MGNCCGTAGSL	60.935	6.41
AtCPK33	At1g50700	****MGNCLAKKYGL	58.605	6.44
AtCPK34	At5g19360	****MGNCCSHGRDS	58.173	5.93
Brachypodium distachyon				
BdCPK1-1	Bradi4g07280	***MGNVCCVGPRRS	67.783	6.34
BdCPK1-2	Bradi1g56970	***MGNTCAGPSAAP	62.416	5.34
BdCPK2	Bradi1g06300	***MGNTCVGPSIAK	68.479	6.23
BdCPK3-1	Bradi2g43910	ATGQRFACKSIATRK	59.439	6.19
BdCPK3-2	Bradi2g15520	EVVNVVHCCHSMGVM	62.120	7.06
BdCPK5-1	Bradi5g19430	***MGNACGGSLRSK	61.589	5.80
BdCPK5-2	Bradi3g51970	***MGNTCGVTFRSM	61.223	5.58
BdCPK5-3	Bradi3g60750	**MGNYSCRDSNSST	58.498	5.33
BdCPK6	Bradi1g76560	NGTLGTDCYHNRYPR	61.512	5.69
BdCPK7-1	Bradi4g39870	****MGNCCVARPSF	63.303	6.55
BdCPK7-2	Bradi1g04440	****MGNCCGAPSSS	59.957	6.78
BdCPK7-3	Bradi3g41770	****MGGCYSAYACS	63.735	6.95
BdCPK12	Bradi4g24390	QFGTTYQCVAKEGGG	57.053	5.50

BdCPK13-1	Bradi2g54080	****MGNCCRSPAAA	63.258	6.95
BdCPK13-2	Bradi2g22750	****MGNCCRSPAAA	60.794	6.71
BdCPK16-1	Bradi1g52567	****MGLCSSSSAAS	57.848	7.80
BdCPK16-2	Bradi3g02600	****MGGCFSTNTPA	58.642	8.89
BdCPK17-1	Bradi2g52870	****MGNCCPGSGDA	56.780	5.65
BdCPK17-2	Bradi4g26317	****MGQCCSKGAAA	58.444	5.88
BdCPK20	Bradi1g06270	***MGNNCVGPNAPG	64.593	5.49
BdCPK24	Bradi4g35100	****MGGCYSVIAAS	62.541	6.31
BdCPK29	Bradi5g18250	****MGNCCAKTYEM	64.049	7.02
BdCPK30	Bradi1g12150	***MGNACLFCCTTT	68.738	9.28
BdCPK32	Bradi1g24240	****MGNCCVTAGEG	60.497	7.39
BdCPK33	Bradi1g26310	****MGQCCSRATSP	59.378	6.18
BdCPK34-1	Bradi2g21390	****MGNCCPGSKDA	56.943	5.88
BdCPK34-2	Bradi4g43400	****MGQCCTTGAAQ	60.068	5.08
Brassica rapa				
BrCPK1	Bra009420	***MGNTCVGPSRNG	65.760	5.58
BrCPK2	Bra005824	***MGNTCVGPSRNG	67.516	5.35
BrCPK3-1	Bra013719	ETKKVFACKSIPTRR	58.804	6.54
BrCPK3-2	Bra019282	****MELCEGGELFD	42.626	5.18
BrCPK3-3	Bra019281	ETKKLFACKSIPTRR	59.488	6.78
BrCPK3-4	Bra019284	ETKKLFACKSIPTRR	59.488	6.78
BrCPK4	Bra000684	QFGTTYLCTEKSSSA	56.284	5.37
BrCPK5	Bra011605	***MGNSCRRSSKDK	61.161	5.76
BrCPK6-1	Bra009653	***MGNSCRGSFKDK	60.592	5.38
BrCPK6-2	Bra037277	***MGNSCRGSFKDK	61.766	5.42
BrCPK7	Bra008879	****MGNCCGSPSSA	59.466	6.25
BrCPK8-1	Bra020040	****MGNCCASPGSD	60.143	6.41
BrCPK8-2	Bra002237	EFGITYLCTDINTGE	54.177	5.88
BrCPK9	Bra001789	****MGNCFAKNHGL	60.710	5.92
BrCPK10-1	Bra031055	****MGNCNVCVRPP	60.150	7.09
BrCPK10-2	Bra025696	EFGITHLCTDRETRE	60.295	7.26
BrCPK11-1	Bra034407	QFGTTYLCTEKSTSA	56.131	5.38
BrCPK11-2	Bra029376	QYGTTSLCTHKETGQ	52.690	5.25
BrCPK11-3	Bra002324	QFGTTFLCTHSETGQ	55.754	5.14
BrCPK11-4	Bra029377	ELKDSVRCVGSELVE	48.020	5.58
BrCPK12	Bra026489	KTAQKLACKSIPKRK	54.997	5.47
BrCPK13-1	Bra033476	****MGNCCRSPAAV	59.513	7.09
BrCPK13-2	Bra012835	****MGNCCRSPAAV	59.465	6.62

BrCPK13-3	Bra006881	****MGNCCRSPAAV	59.430	6.94
BrCPK14-1	Bra004656	****MGNCCGTAGSL	60.278	7.02
BrCPK14-2	Bra016909	****MGNCCGTGGTL	60.145	7.75
BrCPK15	Bra013575	*****MGCFSSKHRR	64.846	6.06
BrCPK16	Bra024487	****MGLCFSSAKVS	65.701	9.3
BrCPK17-1	Bra008910	****MGNCCSNGRDS	57.842	6.21
BrCPK17-2	Bra006142	****MGNCCSSGRDSE	58.238	6.00
BrCPK17-3	Bra029378	QFGTTFLCSHKETGQ	54.629	5.30
BrCPK18	Bra017746	****MGLCFSSPKAT	61.522	8.03
BrCPK20	Bra000105	***MGNTCTGPKLNNP	62.867	5.69
BrCPK21	Bra018504	*****MGCLSSKHRQ	58.658	6.6
BrCPK22-1	Bra029513	*****MGCCGSKSLP	54.278	5.69
BrCPK22-2	Bra018243	****MGCFWSKNQP	48.271	5.90
BrCPK24-1	Bra022844	****MGSCVSSPLKG	62.430	5.99
BrCPK24-2	Bra018236	****MGSCVSSPLKG	66.302	6.86
BrCPK24-3	Bra021727	EFGVTHECIEITTRE	67.353	6.11
BrCPK26	Bra033557	**MARVPCDLRFLLI	101.896	6.84
BrCPK28-1	Bra012058	****MGVCFSAIRVT	60.025	8.59
BrCPK28-2	Bra037181	****MGVCFSAIRVT	60.805	8.85
BrCPK29	Bra015796	****MGFCFSKSQTQ	59.518	6.12
BrCPK30	Bra015896	****MGNCIACVNFD	61.954	6.81
BrCPK32-1	Bra003287	****MGNCCGTAGSF	60.842	6.29
BrCPK32-2	Bra007334	****MGNCCGSAGSL	61.315	6.7
BrCPK34-1	Bra020035	****MGNCCGRDTGN	57.733	5.84
BrCPK34-2	Bra002231	****MGNCCAHGREE	57.676	5.68
BrCPK34-3	Bra023367	QFGVTHLCTQKATGQ	57.668	6.03
Capsella rubella				
CrCPK1	Carubv10000481m.g	*****MGNTCVGPSRNG	69.221	5.56
CrCPK4	Carubv10000785m.g	QFGTTYLCTEKSSSA	56.507	5.27
CrCPK5	Carubv10004491m.g	***MGNSCRGSFKDK	62.659	5.67
CrCPK6	Carubv10013380m.g	***MGNSCRGSFKDK	61.834	5.44
CrCPK7	Carubv10002666m.g	****MGNCCGNPSSA	60.215	6.12
CrCPK8	Carubv10000678m.g	****MGNCCASPGPD	59.978	6.45
CrCPK9	Carubv10013403m.g	****MGNCFAKNNGL	60.211	6.30
CrCPK10	Carubv10008777m.g	****MGNCNACVRPD	61.570	6.68
CrCPK11	Carubv10008949m.g	QFGTTYLCTEKSTSA	55.900	5.56
CrCPK12	Carubv10000821m.g	QFGTTFLCTHKQTGQ	55.260	5.62
CrCPK13	Carubv10017005m.g	****MGNCCRSPAAV	59.317	6.68

CrCPK14	Carubv10025074m.g	****MGNCCGTAGSL	60.125	6.95
CrCPK15	Carubv10004493m.g	*****MGCFSSKHRN	62.975	5.91
CrCPK16	Carubv10013325m.g	****MGLCFSSAAKA	65.141	9.26
CrCPK17	Carubv10002812m.g	****MGNCCSHGRDS	58.770	6.06
CrCPK18	Carubv10006766m.g	****MGLCCSSPKAT	60.768	8.68
CrCPK19	Carubv10021616m.g	*****MACLCINLKK	62.425	6.86
CrCPK20	Carubv10025314m.g	***MGNTCVGPNLNA	64.896	5.78
CrCPK21	Carubv10003219m.g	*****MGCFSSKHRK	60.359	6.78
CrCPK22	Carubv10000880m.g	****MGCCGSKPLT	52.931	5.63
CrCPK23	Carubv10000714m.g	*****MGCFSSKHRE	59.024	5.96
CrCPK24	Carubv10025444m.g	****MGSCVSSPLKG	66.268	6.74
CrCPK26	Carubv10006526m.g	HSGGNQACYVLGQKT	53.052	5.89
CrCPK27	Carubv10003655m.g	*****MGCFISKDPK	54.716	5.66
CrCPK28	Carubv10028399m.g	****MGVCFSAIRVT	60.787	8.82
CrCPK29	Carubv10020082m.g	RKKIMGFCFSKSQTH	63.284	7.87
CrCPK30	Carubv10020106m.g	****MGNCIACVFKD	61.539	6.73
CrCPK31	Carubv10000863m.g	*****MGCYSSKYLR	54.206	6.25
CrCPK32-1	Carubv10016977m.g	****MGNCCGTAGSL	60.899	6.51
CrCPK32-2	Carubv10004572m.g	ETKQQFACKSIPTRR	59.451	6.46
CrCPK33	Carubv10008860m.g	****MGNCLAKKYGV	58.334	6.06
CrCPK34	Carubv10003311m.g	***MGNCCSHGRDS	58.082	6.02
Carica papaya				
CpCPK1-1	evm.TU.supercontig_33.122	QFGTTFLCVEKETGK	62.087	5.51
CpCPK1-2	evm.TU.supercontig_222.24	***MGNACVHMGSNC	70.105	5.74
CpCPK2	evm.TU.supercontig_3109.1	LTAHEVLCHPWIVDD	26.134	4.88
CpCPK3	evm.TU.supercontig_122.15	****MGNCNGMPSNR	59.427	6.50
CpCPK5	evm.TU.supercontig_60.3	***MGNTCRGSFKGK	63.459	5.82
CpCPK7	evm.TU.supercontig_84.46	***MGNCCATPSSSS	59.643	7.02
CpCPK10	evm.TU.supercontig_17.194	****MGNCNACVRPE	62.097	6.78
CpCPK12	evm.TU.supercontig_138.29	QFGTTYLCTHKQTAE	51.014	5.48
CpCPK16	evm.TU.supercontig_6.254	****MGTCLSTTKVS	57.201	8.97
CpCPK17	evm.TU.supercontig_157.56	****MGNCCSQGNSA	58.746	5.83
CpCPK21	evm.TU.supercontig_152.12	*****MGCCSSKPKA	61.898	6.37
CpCPK24	evm.TU.contig_33100	****MGSCVSSQVKS	65.999	7.35
CpCPK29	evm.TU.supercontig_26.269	****MGLCFSKSSSQ	58.285	5.52
CpCPK32	evm.TU.supercontig_92.31	***MGNCCATPSTGT	60.449	7.08
CpCPK33	evm.TU.supercontig_12.305	****MGCCFSDGKG	59.107	6.24
Chlamydomonas reinhardtii				

CreinCPK2	Cre01.g009500	*****MGCSSSKDEV	82.622	6.88
CreinCPK4	Cre13.g564500	*****MGCSSSKPSY	107.598	7.32
CreinCPK9	Cre17.g705000	PTYADTDCWKDYEPG	67.447	5.77
CreinCPK12	g2888	****MVSCFSCGSGE	61.477	5.79
CreinCPK17-1	Cre08.g382800	SGRGATTCGTFIPGL	75.763	6.14
CreinCPK17-2	Cre07.g328900	ISKAKLVCKEDVKDV	53.984	6.21
CreinCPK17-3	Cre02.g074370	****MGGCHSKKTSS	179.971	7.67
CreinCPK17-4	Cre02.g114750	*****MGCSSSIAAK	107.254	6.23
CreinCPK17-5	Cre02.g106650	****MGACASKGGPD	198.916	7.36
CreinCPK17-6	g86	*****MGCSSSKPGV	78.553	6.47
CreinCPK17-7	Cre10.g418900	****MGLCLSKAPLG	184.179	8.34
CreinCPK20-1	Cre06.g265550	LQAAEITCRQAVTPQ	67.966	5.42
CreinCPK20-2	Cre13.g571700	***MGNLCSCVGEGQ	56.264	6.37
CreinCPK34	g18129	****MGNCSSQDNTV	61.007	6.16
Citrus clementina				
CcCPK1	Ciclev10014759m.g	QFGTTFLCVECGTGK	63.508	5.66
CcCPK3	Ciclev10019724m.g	****MGNCNGLPSSQ	58.351	6.50
CcCPK4	Ciclev10000931m.g	QFGTTYLCIHKTTNA	56.144	5.46
CcCPK8	Ciclev10014873m.g	***MGNCCARPDSSS	59.805	6.23
CcCPK9-1	Ciclev10010314m.g	*****MGCIFSKGQD	55.909	5.56
CcCPK9-2	Ciclev10007978m.g	HAKQQDYCHGNPSQA	60.710	8.35
CcCPK9-3	Ciclev10007983m.g	****MGGCLTKSKDP	59.180	6.74
CcCPK9-4	Ciclev10010522m.g	*****MGCIFSKGQD	51.501	5.69
CcCPK10	Ciclev10019541m.g	****MGNCYACVRPS	63.333	7.94
CcCPK12	Ciclev10025418m.g	DTYEDKSCVHIVMEL	57.073	5.48
CcCPK13	Ciclev10011471m.g	***MGNCCRSPAAVA	59.469	6.30
CcCPK15	Ciclev10014823m.g	*****MGCWGSKERV	61.234	6.61
CcCPK17	Ciclev10014854m.g	***MGNCCSNGKDES	59.052	6.10
CcCPK20	Ciclev10011369m.g	***MGNTCVGPNLNS	63.923	5.88
CcCPK24	Ciclev10024502m.g	****MGSCISLARKD	61.166	5.78
CcCPK25	Ciclev10011252m.g	***MGNNCVGSRVSK	72.917	6.19
CcCPK26	Ciclev10028110m.g	***MGNTCRGSFTGK	63.224	5.68
CcCPK28	Ciclev10025251m.g	****MGICLSTTKVS	65.858	9.05
CcCPK29	Ciclev10000897m.g	****MGLCFTRSRSH	58.329	6.11
CcCPK32	Ciclev10004707m.g	***MGNCCVTPAPAK	60.350	7.42
CcCPK33-1	Ciclev10010666m.g	****MGGCLSKIPGS	55.901	7.28
CcCPK33-2	Ciclev10010555m.g	****MGGCLSKILGF	57.014	5.64
CcCPK33-3	Ciclev10007950m.g	****MGGCLRKILCC	60.829	6.77

CcCPK33-4	Ciclev10007980m.g	KPYEDVKCHYTMGKE	60.157	7.06
CcCPK33-5	Ciclev10007873m.g	****MGGCLRKILCC	63.897	6.45
CcCPK33-6	Ciclev10008020m.g	*****MGCIFSKGQD	58.655	5.92
Citrus sinensis				
CsCPK1	orange1.1g007353m.g	QFGTTFLCVEKGTGK	68.050	6.20
CsCPK3	orange1.1g010013m.g	****MGNCNGLPSSQ	58.269	6.38
CsCPK8	orange1.1g009594m.g	***MGNCCARPDSSS	59.805	6.23
CsCPK9-1	orange1.1g009658m.g	****MGGCLTKSKDP	59.180	6.74
CsCPK9-2	orange1.1g042823m.g	*****MGCSVSKNQR	54.210	5.31
CsCPK9-3	orange1.1g009561m.g	HAKQQDYCHGNPSQA	60.710	8.35
CsCPK9-4	orange1.1g047606m.g	****MGGCLSKIPGS	54.189	7.25
CsCPK11	orange1.1g010806m.g	QFGTTYLCIHKTTNA	56.144	5.46
CsCPK12	orange1.1g014949m.g	DTYEDKSCVHIVMEL	46.886	5.14
CsCPK13	orange1.1g009731m.g	***MGNCCRSPAAVA	59.469	6.30
CsCPK20	orange1.1g040003m.g	***MGNTCVGPNLNS	54.617	6.24
CsCPK24	orange1.1g043828m.g	****MGSCISLARKG	66.241	6.57
CsCPK25	orange1.1g006259m.g	***MGNNCVGSRVSK	72.961	6.25
CsCPK26	orange1.1g007895m.g	***MGNTCRGSFTGK	65.508	5.75
CsCPK28	orange1.1g008127m.g	****MGICLSTTKVS	65.827	8.99
CsCPK29	orange1.1g010263m.g	****MGLCFTRSRSH	58.315	6.00
CsCPK30	orange1.1g008668m.g	****MGNCFACVRPS	63.343	7.94
CsCPK31	orange1.1g009091m.g	*****MGCWGSKERV	61.249	6.61
CsCPK32	orange1.1g009367m.g	***MGNCCVTPAAAK	60.697	7.42
CsCPK33-1	orange1.1g018604m.g	*MTVANVCHSKGVMH	40.588	6.13
CsCPK33-2	orange1.1g009382m.g	QYGIIYLCIENSTGR	60.911	7.45
CsCPK33-3	orange1.1g040917m.g	*****MGCIFSKGQD	55.739	5.89
CsCPK33-4	orange1.1g010756m.g	****MGGCLSKILGF	57.042	5.64
CsCPK33-5	orange1.1g010164m.g	*****MGCIFSKGQD	58.655	5.92
Coccomyxa subellipsoidea				
CsubCPK4	estExt_fgenesh1_pg.C_20259	ADNRQYACKSINKAK	57.374	6.68
CsubCPK34	estExt_fgenesh1_pg.C_90258	****MGNCLPKFGKK	53.596	5.19
Cucumis sativus				
CsatCPK1	Cucsa.242110	***MGNNCLRRNSKG	72.519	5.04
CsatCPK2	Cucsa.282830	***MGNTCVGPSISK	63.855	5.56
CsatCPK3	Cucsa.107040	AKGHYSECTAASLCR	45.364	5.41
CsatCPK4-1	Cucsa.240680	QFGTTYLCTHRATGD	56.292	5.60
CsatCPK4-2	Cucsa.228290	QTGFNYACKTIPKRK	56.296	5.21
CsatCPK8-1	Cucsa.164370	***MGNCCVAPPRNP	60.420	6.92

CsatCPK8-2	Cucsa.124160	***MGNCCATPSTPS	59.532	7.40
CsatCPK8-3	Cucsa.143420	***MGNCCATPATPS	59.652	6.73
CsatCPK9	Cucsa.059990	****MGICTSKGKYS	58.602	6.81
CsatCPK10	Cucsa.098010	****MGNCNACVRPE	62.134	6.45
CsatCPK13	Cucsa.176420	***MGNCCRSPAAVA	59.454	6.23
CsatCPK17	Cucsa.086940	***MGNCCSRENPEE	59.800	6.03
CsatCPK20	Cucsa.242210	QFGTTFLCVEKASGK	62.355	5.46
CsatCPK21	Cucsa.045250	*****MGCCSSTQMP	61.560	6.17
CsatCPK24	Cucsa.095230	****MGSCVSIQARP	60.006	5.55
CsatCPK26	Cucsa.099800	***MGNTCRGSFKGN	63.334	5.92
CsatCPK28	Cucsa.043570	****MGVCFSASKVS	61.596	8.63
CsatCPK29	Cucsa.343230	****MGLCFTRTRDI	60.228	6.10
Eucalyptus grandis				
EgCPK1	Eucgr.G02664	***MGNTCVGPSITK	64.957	5.73
EgCPK3	Eucgr.E00806	****MGNCNGLPSAY	59.876	6.75
EgCPK4	Eucgr.F02611	QFGTTYLCTHKATQN	56.880	5.61
EgCPK8	Eucgr.J00760	***MGNCCVTPAPSS	61.238	7.02
EgCPK10	Eucgr.K02914	****MGNCNACVRTE	61.792	6.61
EgCPK13	Eucgr.A00519	***MGNCCRSPAAVA	60.157	6.36
EgCPK14	Eucgr.A02375	***MGNCCATPQAEG	59.401	6.66
EgCPK20	Eucgr.A02545	***MGNSCVGPKLGA	64.341	5.31
EgCPK21	Eucgr.E04057	*****MGCRGSKEKS	60.963	6.64
EgCPK24-1	Eucgr.A00262	****MGGCISAPAKR	60.530	5.52
EgCPK24-2	Eucgr.A00058	VYIMMELCEGGELFD	48.597	5.14
EgCPK25	Eucgr.A02554	***MGNNCVGSRVSR	68.408	6.26
EgCPK26-1	Eucgr.I01536	***MGNTCRGSFGGK	64.047	5.92
EgCPK26-2	Eucgr.H04992	***MGNACQGSLNDK	63.103	5.61
EgCPK28-1	Eucgr.J02432	****MGLCISTTRVS	61.177	9.10
EgCPK28-2	Eucgr.I02347	****MGICASSAARR	61.129	8.73
EgCPK29-1	Eucgr.L01282	***MGNCCATPPQAEG	59.401	6.66
EgCPK29-2	Eucgr.F01695	****MGLCCFRSQDI	55.535	5.17
EgCPK29-3	Eucgr.F01694	****MGLCCSRSQDI	59.848	6.61
EgCPK33-1	Eucgr.F00807	*****MGCCFTKDKA	61.388	6.88
EgCPK33-2	Eucgr.F00761	*****MGCCFTKDKA	58.325	6.89
EgCPK34	Eucgr.J00686	****MGNCCFRGGPA	59.475	6.54
Fragaria vesca				
FvCPK3	gene31992-v1.0-hybrid	***MGGNCSSAAADS	61.270	5.92
FvCPK7	gene25220-v1.0-hybrid	***MGNCCVTPPQTG	62.082	6.70

FvCPK8	gene14687-v1.0-hybrid	***MGNCCVCLGNGA	58.280	5.98
FvCPK9	gene19615-v1.0-hybrid	*****MGCCGSLPKP	61.074	6.60
FvCPK11-1	gene27440-v1.0-hybrid	QFGTTYLCTHKTTGE	55.346	5.74
FvCPK11-2	gene05409-v1.0-hybrid	QFGTTYLCTDKSTGL	55.944	5.08
FvCPK13	gene13451-v1.0-hybrid	***MGNCCRSPAAVA	59.234	6.50
FvCPK20	gene18135-v1.0-hybrid	***MGNTCVGPNLGN	78.786	6.16
FvCPK25	gene18254-v1.0-hybrid	***MGNNCVGNSKAG	78.881	6.31
FvCPK26	gene17341-v1.0-hybrid	***MGNTCRGSYRGK	63.587	6.16
FvCPK28	gene14609-v1.0-hybrid	****MGGCLTTAKVT	62.202	8.77
FvCPK29	gene08576-v1.0-hybrid	****MGLCFTKTSHT	62.846	6.19
FvCPK33	gene03391-v1.0-hybrid	****MGKCLSSKREP	61.085	7.01
FvCPK34	gene15357-v1.0-hybrid	***MGNCCSQRNTED	75.305	7.04
		Glycine max		
GmCPK1	Glyma02g34890	***MGNNCVGSRTSS	65.598	6.46
GmCPK2-1	Glyma10g10501	***MGNNCVGSRTSS	66.223	6.55
GmCPK2-2	Glyma20g17020	***MGNTCVGPSISK	64.968	5.43
GmCPK3-1	Glyma11g02260	****MGNCNSEPSSQ	57.130	6.81
GmCPK3-2	Glyma01g43241	DTVSMGNCNSEASSQ	61.591	7.68
GmCPK3-3	Glyma05g37260	****MGNCSSGAGAP	58.251	6.41
GmCPK4-1	Glyma10g36100	QFGTTYLCTHKVTGK	55.342	5.73
GmCPK4-2	Glyma20g31510	QFGTTYLCTHKVTGK	53.976	5.32
GmCPK8-1	Glyma19g32260	***MGNCCATPSTD	60.287	6.51
GmCPK8-2	Glyma10g17561	***MGNCCVVPSGQT	60.175	6.55
GmCPK9-1	Glyma18g11030	*****MGCCVSKDSE	61.937	6.71
GmCPK9-2	Glyma08g42850	*****MGCCASKESE	61.824	6.58
GmCPK9-3	Glyma02g46070	*****MGCCMSKKGS	59.491	6.80
GmCPK10-1	Glyma06g20170	****MGNCNVCAKAD	62.485	6.45
GmCPK10-2	Glyma04g34440	****MGNCNACAKAD	62.889	6.45
GmCPK11-1	Glyma06g16920	QFGTTFLCTHNATGR	55.996	5.35
GmCPK11-2	Glyma04g38150	QFGTTFLCTHKGTGR	55.941	5.61
GmCPK11-3	Glyma08g00840	QFGTTFECTRRASGG	57.168	5.38
GmCPK11-4	Glyma05g33240	QFGTTFECTRRASGG	57.063	5.44
GmCPK13	Glyma07g18310	***MGNCCRSPAAVA	59.720	6.23
GmCPK17-1	Glyma14g04010	***MGNCCSGGTNTD	58.972	5.99
GmCPK17-2	Glyma02g44720	***MGNCCSGGTNDT	58.684	5.84
GmCPK17-3	Glyma20g08140	****MGNCCSQGNVA	60.878	5.76
GmCPK17-4	Glyma07g36000	****MGNCCSQGNGA	60.348	5.62
GmCPK20	Glyma10g11020	***MGNNCVGPNVGN	65.752	5.55

GmCPK21-1	Glyma17g01730	*****MGCQGSKEKK	59.761	6.17
GmCPK21-2	Glyma07g39010	*****MGCHHSKEKK	58.721	6.21
GmCPK24-1	Glyma12g05730	****MGSCISTQGVR	60.599	5.90
GmCPK24-2	Glyma11g13740	****MGSCITQAVR	61.652	6.16
GmCPK26-1	Glyma14g00320	***MGNTCRGSLKGK	62.719	5.82
GmCPK26-2	Glyma02g48160	***MGNTCRGSLKGK	61.540	5.83
GmCPK28-1	Glyma02g05440	****MGACFSATKVS	60.134	9.21
GmCPK28-2	Glyma11g08180	****MGICFSATKVS	63.483	8.97
GmCPK29-1	Glyma14g40090	GMFKALFCCSKPHEI	59.352	5.38
GmCPK29-2	Glyma17g38040	YMTKYYICVHKETLL	66.930	6.40
GmCPK30-1	Glyma17g10410	****MGNCNACVRVD	61.353	6.20
GmCPK30-2	Glyma05g01470	****MGNCNACVRVD	61.104	6.51
GmCPK30-3	Glyma18g43160	***MGNCCRSPAAVA	57.863	6.12
GmCPK32-1	Glyma02g31490	***MGNCCVLPSRQI	60.029	6.65
GmCPK32-2	Glyma03g29450	***MGNCCATPSTDE	60.093	6.60
GmCPK33	Glyma14g02680	*****MGCCLSKKGS	60.687	6.96
Gossipium raimondii				
GrCPK1-1	Gorai.013G003100	****MGNTCVGPSISK	64.624	5.98
GrCPK1-2	Gorai.011G228500	***MGNTCVGPSISK	65.360	5.47
GrCPK1-3	Gorai.006G147600	***MGNNCFKTISNS	68.356	5.31
GrCPK2-1	Gorai.009G394700	***MGNVCATLGICS	71.846	5.46
GrCPK2-2	Gorai.002G153600	***MGNSCAKSAATE	66.336	5.51
GrCPK3-1	Gorai.007G025000	****MGNCNRPPIVS	60.384	6.68
GrCPK3-2	Gorai.003G084000	****MGNCNGLPSTG	57.546	6.70
GrCPK3-3	Gorai.008G251000	****MGNCNSQPSAD	60.243	6.43
GrCPK4	Gorai.009G290200	QFGTTYLCTDKVTGI	60.417	6.71
GrCPK6-1	Gorai.013G064400	QFGVTYLCTEISTGI	54.635	5.23
GrCPK6-2	Gorai.013G064500	***MGNTCLGSFKGN	58.305	6.40
GrCPK6-3	Gorai.005G216500	***MGNTCRGSLKGK	63.599	6.03
GrCPK6-4	Gorai.008G013700	**MRLHYCMRLHYYI	64.604	6.14
GrCPK6-5	Gorai.012G138900	***MGNTCRGPSKGN	61.957	5.46
GrCPK7-1	Gorai.006G128200	***MGNCCATPGSPV	59.846	6.68
GrCPK7-2	Gorai.006G137800	***MGNCCVTSAAAS	60.215	6.78
GrCPK8	Gorai.001G138000	***MGNCCATTGPLV	59.459	7.36
GrCPK9-1	Gorai.002G088800	****MGSCLTKSKDS	60.457	6.59
GrCPK9-2	Gorai.013G159300	****MGGCLTKNKDP	60.427	6.41
GrCPK9-3	Gorai.009G438300	****MGGCLTKTKGS	58.744	6.50
GrCPK10	Gorai.007G035100	MGNCNACVRPDESP	62.284	6.57

GrCPK11-1	Gorai.005G074300	QFGTTYECIHKATGT	56.857	5.75
GrCPK11-2	Gorai.012G045700	QFGTTYLCTHKPTGQ	55.917	5.52
GrCPK13-1	Gorai.012G114600	***MGNCCRSPAAVA	59.434	6.45
GrCPK13-2	Gorai.007G378700	***MGNCCRSPAAVA	59.244	6.45
GrCPK17-1	Gorai.009G078000	***MGNLCSRSDPAA	59.506	5.59
GrCPK17-2	Gorai.004G015100	****MGNCCSRGNPE	60.511	5.44
GrCPK17-3	Gorai.013G253100	***MGNCCTRGDGSD	58.791	6.46
GrCPK17-4	Gorai.001G135000	***MGNCCSRGSPAA	57.337	5.47
GrCPK17-5	Gorai.006G124800	***MGNCCSCGSSAE	58.594	5.76
GrCPK20	Gorai.009G395400	***MGNACAGPSNLG	63.557	5.44
GrCPK21-1	Gorai.010G252400	*****MGCCSSKNRL	59.318	5.55
GrCPK21-2	Gorai.011G014200	*****MGCFSSKHKP	61.954	6.27
GrCPK24-1	Gorai.005G019800	****MGSCVARPSKL	60.714	5.73
GrCPK24-2	Gorai.009G351200	****MGSCISAPRKL	60.716	7.22
GrCPK28-1	Gorai.011G098300	****MGICLSTTKVF	61.968	8.61
GrCPK28-2	Gorai.003G009500	****MGACLSATKVS	61.273	8.75
GrCPK28-3	Gorai.007G194500	****MGACLSTTKVI	62.788	8.99
GrCPK29	Gorai.010G001300	****MGLCQSLGFCL	59.088	6.33
GrCPK32	Gorai.003G092900	***MGNCCATPSTTA	60.551	6.87
Linum usitatissimum				
LuCPK1-1	Lus10028862.g	***MGNNCVGPSATK	66.608	5.42
LuCPK1-2	Lus10008958.g	QFGTTFLCVEKATGN	55.175	5.54
LuCPK2-1	Lus10029358.g	VHVVMELCSGGELFD	48.224	5.06
LuCPK2-2	Lus10013603.g	***MGNNCVQPKGAY	72.419	6.57
LuCPK2-3	Lus10021248.g	***MGNNCVQAKGAY	72.570	6.74
LuCPK3	Lus10017537.g	ETEQLACKSIAARK	52.032	5.83
LuCPK4	Lus10009427.g	QFGITYLCTHKASNNI	55.773	5.82
LuCPK6-1	Lus10026559.g	***MGNTCRGSFKGK	61.473	5.59
LuCPK6-2	Lus10013842.g	***MGNTCRGSFKGK	61.456	5.72
LuCPK6-3	Lus10001384.g	***MGNTCRGSFKGK	63.001	5.93
LuCPK6-4	Lus10022986.g	***MGNTCRGSFKGK	62.862	5.99
LuCPK7	Lus10025528.g	VHLVMELCQGGELFD	40.297	5.70
LuCPK8-1	Lus10027361.g	***MGNCCASPGNSS	60.155	6.96
LuCPK8-2	Lus10014907.g	***MGNCCASPGNSS	87.336	6.92
LuCPK8-3	Lus10009947.g	***MGNCCTSPATSS	61.284	6.80
LuCPK8-4	Lus10030134.g	***MGNCCTSPATSS	56.300	6.74
LuCPK9-1	Lus10021531.g	****MGHCISKGGRG	60.643	7.01
LuCPK9-2	Lus10040071.g	****MGHCISKGGRG	60.702	6.88

LuCPK9-3	Lus10032640.g	****MGHCISKSAGK	57.724	6.37
LuCPK10	Lus10008631.g	****MGNCNACVRPD	63.426	7.52
LuCPK11-1	Lus10017911.g	TSNMVYACKSISKRK	55.821	6.18
LuCPK11-2	Lus10014820.g	QFGITYLCTHKTSNT	55.660	6.00
LuCPK11-3	Lus10029547.g	QFGTTYLCTHKSTGH	57.311	5.41
LuCPK11-4	Lus10039623.g	QFGTTYLCTHKSTGH	57.266	5.35
LuCPK13-1	Lus10004807.g	***MGNCCRSPAAVA	59.377	6.23
LuCPK13-2	Lus10002482.g	***MGNCCRSPAAVA	59.235	6.23
LuCPK13-3	Lus10027808.g	***MGNCCRSPAAVA	59.649	6.05
LuCPK13-4	Lus10005038.g	IVSFKEACEDDNAVH	47.754	5.79
LuCPK16	Lus10028459.g	VFIVMELCEGGELLD	55.761	8.17
LuCPK17-1	Lus10036050.g	****MGNCCSRGNEGD	58.938	5.90
LuCPK17-2	Lus10000889.g	****MGNCCSRGNEG	58.170	5.98
LsCPK20-1	Lus10016200.g	***MGNTCAGPNNVF	66.607	6.29
LuCPK20-2	Lus10012285.g	***MGNTCNGPTSGS	63.317	5.78
LuCPK20-3	Lus10015992.g	***MGNTCNGPTNGS	63.337	6.04
LuCPK20-4	Lus10029346.g	QFGTTFLCIEKATRK	62.913	6.71
LuCPK21-1	Lus10006777.g	*****MGCSSSKGPH	59.102	5.93
LuCPK21-2	Lus10002075.g	***************	33.556	5.04
LuCPK21-3	Lus10020046.g	***MGCSSSKGPH	60.166	6.54
LuCPK24	Lus10027032.g	****MGGCISSAANA	60.668	5.73
LuCPK28	Lus10041914.g	****MGLCFSTSKVS	63.831	8.89
LuCPK29	Lus10017251.g	****MGLCFTRTKSH	57.928	6.86
LuCPK30-1	Lus10036667.g	****MGNCNTCVRPE	56.828	5.65
LuCPK30-2	Lus10042185.g	VHLVMELCEGGELFD	48.888	5.51
LuCPK32-1	Lus10042370.g	***MGNCCVTPLVTT	59.629	6.85
LuCPK32-2	Lus10026742.g	VHLVMELCEGGELFD	56.980	6.37
LuCPK32-3	Lus10025570.g	****MGGCISSAAKA	77.440	6.67
LuCPK34	Lus10038460.g	****MGNCCSRGKEG	58.982	6.27
Malus domestica				
MdCPK1-1	MDP0000153100	***MGNTCVGPSISK	62.753	5.30
MdCPK1-2	MDP0000142687	***MGNTCVGPSISK	68.486	5.65
MdCPK1-3	MDP0000128057	***MGNTCVGPSISK	73.252	6.44
MdCPK2	MDP0000232344	PRDDQIGCQXYLQLS	85.954	6.48
MdCPK4-1	MDP0000260834	QFGTTYLCTHKPTGA	58.051	5.73
MdCPK4-2	MDP0000232885	QFGTTYLCTHKPTGA	58.149	5.91
MdCPK8-1	MDP0000269423	KDGDGKLCVLKDPTG	61.558	7.09
MdCPK8-2	MDP0000119457	***MGNCCVTLGAPG	52.644	6.44

MdCPK8-3	MDP0000260857	***MGNCCATPQTGS	74.052	6.00
MdCPK10-1	MDP0000218522	****MGNCNVCVRAD	64.210	6.89
MdCPK10-2	MDP0000301254	****MGNCNVCVRAD	61.870	7.36
MdCPK10-3	MDP0000308706	****MGNCNVCVRAD	61.870	7.36
MdCPK11	MDP0000494270	QFGTTYLCTEISSGH	55.109	5.35
MdCPK13-1	MDP0000164868	***MGNCCRSPAAVA	65.774	6.64
MdCPK13-2	MDP0000649496	VILYILLCGVPPFWA	39.061	5.36
MdCPK17-1	MDP0000802997	***MGNCCSQCNTED	59.208	6.09
MdCPK17-2	MDP0000138436	***MGNCCSQCNTED	59.754	6.03
MdCPK20-1	MDP0000318339	MKKMNKNCSTVKCGC	114.668	6.26
MdCPK20-2	MDP0000513005	***************	75.738	5.83
MdCPK21	MDP0000232001	*****MGCYSSKENA	61.524	6.50
MdCPK24-1	MDP0000262701	****MGSCLCTPANA	61.346	5.55
MdCPK24-2	MDP000028200	****MGSCVCTPAKA	108.159	5.97
MdCPK26-1	MDP0000297184	***MGNTCRGSFRGK	63.162	6.38
MdCPK26-2	MDP0000457940	IGTPLYLCCRSLTFS	155.079	5.84
MdCPK28	MDP0000208913	****MGICFSAVKVS	67.680	8.86
MdCPK29	MDP0000142398	****MGLCFTKCQSH	59.855	6.25
MdCPK32-1	MDP0000649508	***MGNCCVTLGAPG	78.669	8.71
MdCPK32-2	MDP0000179069	***MGNCCVTPQTGS	74.907	8.35
Manihot esculenta				
MeCPK2	cassava4.1_004466m.g	***MGNTCVGPSISK	64.055	5.20
MeCPK3	cassava4.1_005320m.g	****MGNCSSSSSLP	59.686	6.50
MeCPK6-1	cassava4.1_004779m.g	***MGNTCRGSFKGK	62.573	5.82
MeCPK6-2	cassava4.1_031489m.g	***MGNTCRGSFKGK	62.874	5.83
MeCPK8	cassava4.1_005260m.g	***MGNCCVTPASSS	60.124	6.89
MeCPK11-1	cassava4.1_006083m.g	QFGTTYLCINKATSK	56.753	5.75
MeCPK11-2	cassava4.1_005808m.g	QFGTTYLCTNKATNK	57.583	5.90
MeCPK12-1	cassava4.1_006026m.g	**************	56.559	5.52
MeCPK12-2	cassava4.1_006051m.g	QFGTTYLCTHKPSGL	56.697	5.61
MeCPK17-1	cassava4.1_023323m.g	****MGNCCSRGESG	58.744	5.66
MeCPK17-2	cassava4.1_029242m.g	QFGVTHLCTLKATGE	58.756	5.42
MeCPK20-1	cassava4.1_022914m.g	QKHYGPECDVWSVGV	32.541	4.79
MeCPK20-2	cassava4.1_023345m.g	SKQGNSSCFPYVGEI	34.353	4.85
MeCPK21	cassava4.1_007132m.g	QFGITYLCTENPTAH	52.545	5.63
MeCPK24-1	cassava4.1_021196m.g	****MGCCISAPGKG	61.183	6.31
MeCPK24-2	cassava4.1_031848m.g	****MGGCISAPGKA	60.259	5.79
MeCPK25	cassava4.1_029823m.g	QFGTTFLCVEKGTGK	72.133	5.11

MeCPK28-1	cassava4.1_004577m.g	****MGACFSTIKIS	64.445	8.90
MeCPK28-2	cassava4.1_004333m.g	****MGVCFSTIKVS	66.122	8.89
MeCPK29	cassava4.1_007356m.g	****MGHCFTKSRSH	51.899	6.89
MeCPK30	cassava4.1_004922m.g	****MGNCNACVRAD	62.586	7.02
MeCPK32-1	cassava4.1_005336m.g	***MGNCCVTPSTAA	60.293	6.25
MeCPK32-2	cassava4.1_005345m.g	***MGNCCLTPSRAV	60.388	7.42
MeCPK32-3	cassava4.1_005177m.g	***MGNCCATPASSS	60.531	6.55
MeCPK33-1	cassava4.1_005266m.g	****MGLCLSKDKRT	60.172	6.66
MeCPK33-2	cassava4.1_005323m.g	****MGYCLSKEKNT	59.901	7.14
Medicago truncatula				
MtCPK1	Medtr1g041150	***MGNTCVGPSISK	65.077	5.54
MtCPK3	Medtr5g009830	****************	58.731	6.92
MtCPK4	Medtr3g098090	QFGITYHCTHNSTGR	61.875	5.84
MtCPK11-1	Medtr8g095440	QFGTTYLCTHKSTNK	56.818	5.37
MtCPK11-2	Medtr3g098070	QFGITYHCIHNSTGR	55.921	5.34
MtCPK17-1	Medtr5g089320	***MGNCCSGGTEDP	58.496	5.68
MtCPK17-2	Medtr7g054260	****MGNCCSQGGDT	59.848	5.99
MtCPK21	Medtr4g132070	*****MGCHGSKEKK	60.557	6.60
MtCPK26	Medtr5g099240	KRETEVVCVLLSLRR	66.971	5.91
MtCPK28	Medtr5g022030	****MGLCFSSTKVV	63.499	9.03
MtCPK33	Medtr4g132040	*****MGCYTSKPEV	60.879	6.21
Micromonas pusila				
MpCPK7	e_gw1.4.726.1	TATATAACGGGATPS	55.479	6.30
MpCPK17	estExt_fgenesh2_kg.C_10200	*****MGCAGSKPDP	80.654	5.56
Mimulus guttatus				
MgCPK1	mgv1a003630m.g	***MGNTCVGPGITK	63.827	5.70
MgCPK3	mgv1a004683m.g	******************	57.373	5.85
MgCPK4	mgv1a004202m.g	*****************	60.516	5.44
MgCPK6	mgv1a004107m.g	***MGNTCRGSFGGK	60.589	5.98
MgCPK8	mgv1a004319m.g	***MGNCCVTPSSDK	60.200	6.78
MgCPK9	mgv1a004414m.g	****MGMCFSHQRNG	58.885	6.37
MgCPK10-1	mgv1a004139m.g	****MGNCNACIRPS	61.332	6.33
MgCPK10-2	mgv1a003708m.g	****MGNCNVCFRPS	64.523	6.78
MgCPK11-1	mgv1a005115m.g	QFGTTYHCVEKATGI	55.889	5.54
MgCPK11-2	mgv1a005313m.g	QFGTTFLCTDKSNGS	55.153	5.42
MgCPK13-1	mgv1a004415m.g	***MGNCCRSPAAVA	59.975	6.30
MgCPK13-2	mgv1a004417m.g	***MGNCCRSPAAVA	59.378	5.96
MgCPK16	mgv1a007109m.g	*******************	47.628	6.25

MgCPK17-1	mgv1a004352m.g	****MGSCCSRGTTE	58.948	5.54
MgCPK17-2	mgv1a004350m.g	****MGICCSRSNAD	59.196	5.86
MgCPK20-1	mgv1a003550m.g	***MGNNCVGPNLGN	64.230	5.39
MgCPK20-2	mgv1a003552m.g	***MGNTCVGPKLGN	64.035	5.69
MgCPK24	mgv1a004648m.g	****MGTCMSLQRSS	58.979	5.42
MgCPK25	mgv1a002855m.g	***MGNNCVNGRIAK	70.935	5.97
MgCPK28	mgv1a003874m.g	****MGICFSTKKGS	63.147	9.14
MgCPK29	mgv1a004597m.g	****MGFCLSKFKKS	58.724	6.16
MgCPK32-1	mgv1a004318m.g	***MGNCCATPPSHS	60.057	6.93
MgCPK32-2	mgv1a004372m.g	***MGNCCTSPKTSD	59.881	6.50
MgCPK32-3	mgv1a004430m.g	***MGNCCTVPKTSD	59.747	6.23
MgCPK33	mgv1a004201m.g	****MGGCFSKGKYS	60.767	6.71
Oryza sativa				
OsCPK1	LOC_Os01g43410	ATGKRFACKSIATRK	58.739	6.50
OsCPK2	LOC_Os01g59360	****MGNCCPGSGDA	56.950	5.82
OsCPK3	LOC_Os01g61590	****MGACFSSHTAT	58.575	8.22
OsCPK4	LOC_Os02g03410	**** MGACFSSHTAT	58.575	8.22
OsCPK5	LOC_Os02g46090	***MGNTCGVTLRSK	60.387	5.83
OsCPK6	LOC_Os02g58520	*MGNYYSCGASSTSS	60.259	5.58
OsCPK7	LOC_Os03g03660	***MGNQCQNGTLGS	63.863	5.99
OsCPK8	LOC_Os03g59390	****MGNCCGTPATA	60.657	6.61
OsCPK9	LOC_Os03g48270	***MGNTCCVAPATT	63.977	8.52
OsCPK10	LOC_Os03g57450	***MGNTCVGPSISK	65.861	5.65
OsCPK11	LOC_Os03g57510	***MGNNCVGPSAAG	63.170	5.42
OsCPK12	LOC_Os04g47300	****MGNCFTKTYEI	59.561	6.68
OsCPK13	LOC_Os04g49510	***MGNACGGSLRSK	60.966	5.74
OsCPK14	LOC_Os05g41270	***MGNCCPPGSSSE	57.631	6.16
OsCPK15	LOC_Os05g50810	***************	63.744	7.08
OsCPK16	LOC_Os05g39090	***MGNCCRSPAAAA	60.993	6.89
OsCPK17	LOC_Os07g06740	***MGNTCVGPSSAA	62.618	5.92
OsCPK18	LOC_Os07g22710	****MGLCSSSSARR	57.584	7.77
OsCPK19	LOC_Os07g33110	***MGSCCSRATSPD	59.505	6.32
OsCPK20	LOC_Os07g38120	***MGNCCVTPEGSG	62.407	7.42
OsCPK21	LOC_Os08g42750	****MGGCYSAYASS	61.916	6.19
OsCPK22	LOC_Os09g33910	****MGGCSSAFAVS	63.760	6.84
OsCPK23	LOC_Os10g39420	***MGNSCQNGTYGN	60.546	5.82
OsCPK24	LOC_Os11g07040	QFGTTYLCVDKASGG	56.672	5.40
OsCPK25	LOC_Os11g04170	****MGQCCTGGGKA	59.463	6.29

OsCPK26	LOC_Os12g03970	****MGQCCTGGGKA	59.433	6.29
OsCPK27	LOC_Os12g30150	***MGNVCIGPRRNF	67.580	6.19
OsCPK28	LOC_Os12g07230	QFGTTYLCVGKPDGG	57.988	5.75
OsCPK29	LOC_Os12g12860	***MGNCCVSRPGSA	62.871	7.34
OsCPK30	LOC_Os07g44710	****MGLCHGKPSQI	65.242	8.76
Ostreococcus lucimarinus				
OlCPK3	e_gwEuk.14.119.1	DAFEDDECVHLLMEL	50.868	4.61
OlCPK17	estExt_fgenesh1_pg.C_Chr_30268	****MGVCGSSPAVP	53.171	5.24
OlCPK19	estExt_Genewise_ext.C_Chr_60025	******************	57.497	5.49
Panicum virgatum				
PvCPK1-1	Pavirv00040420m.g	***MGNVCVGNRFSK	68.113	6.07
PvCPK1-2	Pavirv00037785m.g	***MGNTCVGPSITK	66.258	6.14
PvCPK1-3	Pavirv00017178m.g	***MGNTCVGPSITK	67.302	6.26
PvCPK1-4	Pavirv00031162m.g	***MGNTCVGPSASS	62.873	6.19
PvCPK1-5	Pavirv00060314m.g	***MGNTCVGPSAAG	64.078	5.50
PvCPK1-6	Pavirv00010438m.g	***MGNTCVGPSAAG	64.140	5.50
PvCPK1-7	Pavirv00048385m.g	***********	35.697	4.84
PvCPK1-8	Pavirv00011826m.g	***********	35.055	4.94
PvCPK3-1	Pavirv00016837m.g	**********	52.441	5.66
PvCPK3-2	Pavirv00014196m.g	ATGRRYACKSIAARK	49.907	5.80
PvCPK3-3	Pavirv00030399m.g	**********	45.657	5.42
PvCPK3-4	Pavirv00015840m.g	**********	42.142	5.14
PvCPK4-1	Pavirv00064150m.g	QFGTTYQCVGKADGA	56.796	5.83
PvCPK4-2	Pavirv00024283m.g	QFGTTYQCVGKADGA	56.842	5.75
PvCPK4-3	Pavirv00007698m.g	QFGTTYQCVGKADGA	56.891	5.60
PvCPK4-4	Pavirv00002804m.g	*************	52.651	5.62
PvCPK4-5	Pavirv00049634m.g	*************	34.697	4.75
PvCPK5-1	Pavirv00003075m.g	QFGTTYLCTDLATGV	53.888	5.39
PvCPK5-2	Pavirv00044902m.g	QFGTTYLCTELATGV	54.561	5.44
PvCPK5-3	Pavirv00044833m.g	QFGTTYLCTELATGV	54.454	5.51
PvCPK5-4	Pavirv00049417m.g	QFGTTYLCTDLATGV	54.712	6.02
PvCPK5-5	Pavirv00002047m.g	***MGNSCRGSSTPT	62.367	5.72
PvCPK5-6	Pavirv00059889m.g	***MGNSCRGSSTPT	62.748	6.01
PvCPK6	Pavirv00016031m.g	***MGNQCPNGTLGS	61.180	5.49
PvCPK7-1	Pavirv00069650m.g	***********	56.655	6.23
PvCPK7-2	Pavirv00014425m.g	**************	39.803	5.86
PvCPK7-3	Pavirv00016858m.g	***MGNCCVTAGGAA	60.941	6.96
PvCPK8-1	Pavirv00070851m.g	***MGNCCVTAGGAA	60.853	6.85

PvCPK8-2	Pavirv00026354m.g	***MGNCCGTPATQE	59.775	6.73
PvCPK9-1	Pavirv00018537m.g	***MGQCCSRATAPD	59.407	6.13
PvCPK9-2	Pavirv00063027m.g	***MGQCCSRATAPD	59.515	6.25
PvCPK9-3	Pavirv00044023m.g	***MGQCCRATAPD	57.436	6.48
PvCPK13-1	Pavirv00065690m.g	***MGNCCRSPAAAA	60.711	6.70
PvCPK13-2	Pavirv00068905m.g	***MGNCCRSPAAAA	60.695	6.70
PvCPK13-3	Pavirv00021833m.g	****MGGCYSAIAAT	59.380	6.98
PvCPK16-1	Pavirv00029104m.g	****MGLCSSSTAAA	57.526	8.03
PvCPK16-2	Pavirv00048594m.g	*****************	48.744	7.84
PvCPK17-1	Pavirv00035994m.g	QFGVTHLCTRRATGE	52.303	5.77
PvCPK17-2	Pavirv00011855m.g	QFGVTHLCTHRATGE	52.284	5.76
PvCPK17-3	Pavirv00014051m.g	***ERFACKTISKRK	48.725	5.69
PvCPK24	Pavirv00021668m.g	*MLYILLCGVPPFWG	31.330	4.86
PvCPK29-1	Pavirv00054378m.g	****MGNCFTKTYEH	60.626	6.38
PvCPK29-2	Pavirv00016737m.g	*************	44.845	5.50
PvCPK30-1	Pavirv00024932m.g	***MGNVCFCGTTST	63.434	8.75
PvCPK30-2	Pavirv00001784m.g	***MGNVCFCGTTST	63.733	8.50
PvCPK32-1	Pavirv00013424m.g	******LCHSNGVIH	39.568	5.11
PvCPK32-2	Pavirv00011479m.g	GCYSAYACSRKLRGQ	61.972	6.41
PvCPK32-3	Pavirv00068592m.g	***MGNCCVARPSCN	64.030	6.39
PvCPK32-4	Pavirv00018203m.g	***MGNCCVARPSSF	63.673	6.45
PvCPK32-5	Pavirv00049858m.g	SRDGGPRCSPERDSD	59.859	6.38
PvCPK34-1	Pavirv00017949m.g	***MGNCCPGSGDAE	57.385	5.83
PvCPK34-2	Pavirv00007044m.g	***MGNCCPGSGDAE	57.371	5.83
PvCPK34-3	Pavirv00019274m.g	QFGVTSLCTHKATGE	52.139	5.69
Phaseolus vulgaris				
PvuCPK1	Phvul.007G233900	***MGNTCTGPSISK	64.994	5.42
PvuCPK2	Phvul.007G266100	***MGNSCVGSRSSA	65.132	6.12
PvuCPK3-1	Phvul.002G294500	****MGNCSSSHGGG	58.282	6.31
PvuCPK3-2	Phvul.002G161200	HTNENLACKSIAKRK	56.707	6.50
PvuCPK4-1	Phvul.007G089200	QFGTTYLCTHKTTGK	55.159	5.66
PvuCPK4-2	Phvul.007G089300	**************	63.121	6.79
PvuCPK8	Phvul.007G253300	***MGNSCVTPGRIR	58.676	6.96
PvuCPK9-1	Phvul.006G043700	****MEVCLINIYTL	52.205	5.78
PvuCPK9-2	Phvul.008G266600	****MGGCLSKNGSE	59.096	6.78
PvuCPK10	Phvul.002G087800	*MGNCNACAKADVVE	61.992	6.51
PvuCPK11-1	Phvul.009G160100	QFGTTFLCTHNATAR	55.763	5.53
PvuCPK11-2	Phvul.002G279300	QFGTTFLCTRRVGRG	56.933	5.42

PvuCPK13	Phvul.008G098400	***MGNCCRSPAAVA	59.760	6.23
PvuCPK17-1	Phvul.008G201900	***MGNCCSGGTNDC	58.491	5.89
PvuCPK17-2	Phvul.006G015300	****MGNCCSQGNAA	60.538	5.23
PvuCPK20	Phvul.007G265100	***MGNNCVGPNVGN	64.787	5.55
PvuCPK21	Phvul.003G078400	*****MGCHGSKEKK	60.723	6.12
PvuCPK24	Phvul.011G055400	****MGSCISTQGVR	61.069	6.93
PvuCPK25	Phvul.001G197700	***MGNNCVGSKISD	64.554	5.20
PvuCPK26	Phvul.008G292500	***MGNTCRGSLKGK	63.013	5.90
PvuCPK28-1	Phvul.002G108700	****MGICFSATKVS	64.690	9.15
PvuCPK28-2	Phvul.003G261700	****MGICYSATKVS	59.721	5.85
PvuCPK29	Phvul.001G002900	PPPMPTLCSSQTGPV	57.765	5.93
PvuCPK30	Phvul.003G194100	*MGNCNACVRDDVAH	60.894	6.34
PvuCPK32	Phvul.001G135300	****MGICCAKPSTQ	60.980	6.74
Physcomitrella patens				
PpCPK1-1	Pp1s49_208V6	QFGTTYLCVEKTTGK	55.111	5.49
PpCPK1-2	Pp1s49_200V6	QFGTTYLCVEKTTGK	55.111	5.49
PpCPK1-3	Pp1s205_14V6	***MGNTCVGAAGYE	63.784	5.87
PpCPK1-4	Pp1s166_57V6	MNPQQSLCSSLFIRG	60.921	6.23
PpCPK1-5	Pp1s138_79V6	QFGTTYLCTERATGL	55.447	4.78
PpCPK1-6	Pp1s309_91V6	QFGITYLCTEKATGL	55.375	4.84
PpCPK2-1	Pp1s187_88V6	QFGTTYLCVEKATGR	55.251	5.44
PpCPK2-2	Pp1s187_77V6	QFGTTYLCVEKATGR	55.251	5.39
PpCPK7-1	Pp1s232_44V6	***************	58.472	6.45
PpCPK7-2	Pp1s97_71V6	***MGNCCVGSSTKK	59.322	6.61
PpCPK7-3	Pp1s2_191V6	***MGNCCAGSATKK	58.485	6.50
PpCPK7-4	Pp1s2_156V6	***MGNCCAGSATKK	58.485	6.50
PpCPK9	Pp1s143_92V6	QFGVTYLCTHKETGE	68.533	6.56
PpCPK13	Pp1s364_61V6	***MGNCCVGSSSNK	59.795	6.50
PpCPK16-1	Pp1s199_57V6	****MGNCCKKSSVA	66.357	8.52
PpCPK16-2	Pp1s370_37V6	***MGGFCSKTPVVE	62.094	8.49
PpCPK16-3	Pp1s83_172V6	***MGGCCSKSSVVE	66.278	8.63
PpCPK16-4	Pp1s83_8V6	***MGSCCTKSSVVE	74.055	9.01
PpCPK17-1	Pp1s96_216V6	QFGVTYLCTDKMTNE	60.578	6.25
PpCPK17-2	Pp1s316_13V6	***MGNQCVGAIGGP	60.641	6.64
PpCPK17-3	Pp1s325_31V6	QFGVTYLCTDKETTGI	59.700	6.68
PpCPK17-4	Pp1s108_32V6	ERAAADLCRVIVNVV	64.047	6.50
PpCPK17-5	Pp1s108_25V6	ERAAADLCRVIVNVV	62.458	6.50
PpCPK30	Pp1s364_61V6	***MGNCCVGSSTKK	59.893	6.36

Picea abies				
PaCPK3	MA_13110g0010	*******	33.670	5.07
PaCPK5	MA_115550g0010	*******	62.181	6.09
PaCPK6	MA_10429609g0010	*******	34.710	4.79
PaCPK9	MA_98632g0020	MGNRQGRCGKRNYPE	52.949	6.86
PaCPK13	MA_9458g0010	****MGNCCGSAGSK	62.037	6.35
PaCPK15	MA_3553g0010	RLLSFFNCILLFLLI	38.377	4.86
PaCPK17	MA_18543g0010	*****	60.851	8.05
PaCPK21	MA_10435930g0030	MVFRVDCGFILPEK	42.229	4.84
PaCPK29	MA_10435930g0050	*******	35.393	4.98
PaCPK33	MA_70277g0010	*******	32.082	4.61
PaCPK34	MA_10437101g0010	*******	51.236	5.59
Populus trichocarpa				
PtCPK1	Potri.010G244800	***MGNTCVGPSISR	66.283	5.42
PtCPK2	Potri.008G014700	***MGNTCVGPSISK	64.673	5.86
PtCPK3-1	Potri.003G134000	****MGNCNSLPSSS	59.019	6.61
PtCPK3-2	Potri.001G097400	****MGNCNSLASSS	58.240	6.66
PtCPK6-1	Potri.009G168600	***MGNTCRGSFKGI	62.452	5.82
PtCPK6-2	Potri.004G207300	***MGNTCRGSFKGK	62.516	5.62
PtCPK7	Potri.001G257100	***MGNCCVTPTGAS	59.816	6.93
PtCPK8	Potri.009G052700	***MGNCCFTPSGAS	60.001	7.02
PtCPK9	Potri.004G015500	*****MGCFSSKEKA	60.083	6.44
PtCPK11	Potri.019G083200	QFGITYLCTHKASSA	58.271	6.03
PtCPK12	Potri.T012800	QFGTTFLCTHKTSGK	56.603	5.83
PtCPK13-1	Potri.006G101300	***MGNCCRSPAAVA	65.640	6.45
PtCPK13-2	Potri.016G117200	***MGNCCRSPAAVA	59.702	6.23
PtCPK17	Potri.009G069200	****MGNCCSRGGAQ	58.554	6.16
PtCPK20-1	Potri.006G199400	***MGNTCVGPNLGN	66.484	5.33
PtCPK20-2	Potri.016G065700	***MGNTCVGPNLGN	66.364	5.72
PtCPK24	Potri.007G127000	****MGSCVSTPANL	60.699	6.17
PtCPK25	Potri.016G066700	**MGNSNCIGSRFSK	68.502	6.39
PtCPK28-1	Potri.007G057600	****MGACFSTINIS	63.231	8.36
PtCPK28-2	Potri.005G113600	****MGACFSTIKVS	63.055	9.00
PtCPK29-1	Potri.002G017000	****MGLCFSRLSFS	60.804	5.75
PtCPK29-2	Potri.005G245000	GKCFSRSCSHDIPIS	57.622	6.20
PtCPK30-1	Potri.015G066200	****MGNCNTCVRPD	63.967	6.59
PtCPK30-2	Potri.012G071700	****MGNCNTCVRPD	63.148	6.37
PtCPK32-1	Potri.006G052900	***MGNCCVTPSGNP	59.846	6.55

PtCPK32-2	Potri.016G054600	***MGNCCVTPPGVP	60.447	6.78
PtCPK33	Potri.011G003400	*****MGCCGSKGNA	59.927	6.50
PtCPK34	Potri.001G274700	***MGNCCSRGGAQD	56.469	5.93
Prunus persica				
PpeCPK1	ppa003542m.g	***MGNTCVGPSISK	63.193	5.51
PpeCPK2	ppa002734m.g	***MGNNCVGSTKGD	71.299	6.30
PpeCPK3	ppa004162m.g	***MGNNCSSGALPS	59.008	6.50
PpeCPK8-1	ppa006164m.g	******************	48.049	5.73
PpeCPK8-2	ppa004069m.g	***MGNCCVTPQTGS	60.207	6.81
PpeCPK9	ppa003830m.g	****MGKCLSKSKDS	61.168	6.93
PpeCPK10	ppa003795m.g	****MGNCNVCVRAD	62.195	7.33
PpeCPK11	ppa004665m.g	QFGTTYLCIHKPTGD	55.731	5.29
PpeCPK12	ppa004580m.g	QFGTTYLCTESSTGH	56.527	5.32
PpeCPK13	ppa004141m.g	***MGNCCRSPAAVA	59.443	6.36
PpeCPK17	ppa026653m.g	***MGNCCSQRSTED	59.759	5.93
PpeCPK20	ppa003092m.g	***MGNTCVGPNLGG	67.284	5.48
PpeCPK21	ppa006748m.g	VHLVMELCAGGELFD	44.868	5.28
PpeCPK24	ppa021005m.g	****MGSCVCTPAKA	59.679	5.93
PpeCPK26	ppa003459m.g	***MGNTCRGSFRGK	64.304	6.43
PpeCPK28	ppa003676m.g	****MGGCFSTVKVS	62.972	9.21
PpeCPK29	ppa004027m.g	****MGLCFTRCRSH	60.384	6.39
Ricinus communis				
RcCPK1	29852.t000019	***MGNTCVGPTISK	64.237	5.57
RcCPK3	30190.t000579	****MGNCSSNLPST	59.244	6.54
RcCPK6	30100.t000014	***MGNTCRGSFKGK	62.879	5.74
RcCPK8	30169.t000219	***MGNCCTIPSSSS	60.331	6.95
RcCPK11	29728.t000018	QFGTTYLCTNKATNA	56.029	5.54
RcCPK13	28308.t000002	***MGNCCRSPAAVA	59.384	6.19
RcCPK17	30142.t000025	****MGNCCSRGDSG	59.521	6.05
RcCPK20	29333.t000051	***MGNTCVGPNLSF	65.395	5.52
RcCPK21	29842.t000094	*****MGCCGSKEKP	62.094	6.62
RcCPK24	29896.t000003	****MGGCISAPLKA	60.477	6.21
RcCPK25	29333.t000041	***MGNNCVGSRASQ	72.058	6.40
RcCPK29	30170.t000694	****MGLCFTKFHSR	59.734	6.66
RcCPK30	30147.t000328	*MGNCNTCVRPDTSP	62.372	6.85
RcCPK32	29761.t000015	***MGNCCATPPSD	60.101	7.12
RcCPK33	27777.t000008	****MGFCLSKEKRS	60.220	6.74
Selaginella moelendorffii				

SmCPK1	99178	QFGVTYLCVEKSSGK	54.920	5.96
SmCPK2	165073	QFGTTYVCTEKSSGK	55.377	5.66
SmCPK3	118877	QFGVTRLCSRRSTGE	54.680	5.35
SmCPK7	105846	***MGNCCATPPREA	60.131	6.51
SmCPK13	105020	***MGNCCATPGIKR	60.291	6.95
SmCPK16	92726	****MGVCFSVPKRR	56.815	7.97
SmCPK17-1	96034	****MGNCLGGGDEK	55.160	6.45
SmCPK17-2	152133	QFGVTHLCTDRASGK	54.579	5.77
SmCPK28	164119	****MGICFSADKSE	61.819	8.38
Setaria italica				
SiCPK1-1	Si034743m.g	***MGNTCVGPSITK	67.109	6.26
SiCPK1-2	Si034852m.g	***MGNTCVGPSAAG	63.918	5.62
SiCPK1-3	Si029343m.g	***MGNTCVGPSAPS	63.077	5.84
SiCPK2	Si021787m.g	******************	57.017	5.80
SiCPK3-1	Si001025m.g	ETGQRFACKSIATRK	59.025	6.50
SiCPK3-2	Si021672m.g	***************	61.546	6.84
SiCPK4	Si009864m.g	QFGTTYQCVGKADGA	56.766	5.66
SiCPK5-1	Si016803m.g	***MGNTCGVTLRSK	61.394	5.90
SiCPK5-2	Si016805m.g	***MGNSCRGSFTPT	61.063	5.64
SiCPK5-3	Si009748m.g	***MGNACGGSLRSR	61.170	5.96
SiCPK6-1	Si035094m.g	***MGNQCQNGTYGN	60.489	5.10
SiCPK6-2	Si040704m.g	***MGNQCPNGTLGS	59.559	5.43
SiCPK7-1	Si035115m.g	***MGNCCGAPATQG	59.771	7.30
SiCPK7-2	Si021615m.g	***MGNCCVARPSCK	63.087	6.41
SiCPK8	Si029431m.g	***MGNCCMTPGGAA	60.857	6.85
SiCPK9	Si029460m.g	***MGQCCSRATAPD	59.535	6.32
SiCPK11	Si026386m.g	***MWYHCTALVDDS	48.765	5.35
SiCPK13-1	Si000940m.g	***MGNCCRSPAAVA	60.454	6.41
SiCPK13-2	Si021667m.g	*MKPSGNCCRSPAAA	60.934	6.93
SiCPK13-3	Si015574m.g	****MGGCYSAYACS	60.935	7.23
SiCPK13-4	Si029399m.g	****MGGCYSAIAAT	61.953	7.75
SiCPK16	Si016899m.g	****MGACFSSASAA	58.611	8.77
SiCPK20	Si025125m.g	***MGNVCVGSRFSK	69.179	5.95
SiCPK29	Si009791m.g	****MGNCFTKTYEH	60.145	6.51
SiCPK30	Si034847m.g	***MGNVCFCGTTST	64.488	8.86
SiCPK34-1	Si024765m.g	***MGNCCCPGSSSA	58.194	5.89
SiCPK34-2	Si003781m.g	****MELCAGGELFD	42.523	5.41
Solanum lycopersicum				

SlCPK1-1	Solyc04g009800	***MGNTCVGPSISR	64.602	5.80
SlCPK1-2	Solyc11g006370	***MGNTCVGPSISK	64.680	5.66
SlCPK2-1	Solyc10g081740	QFGTTFLCVEKATGE	55.764	5.22
SlCPK2-2	Solyc01g006840	***MGNNCVHAKISK	67.548	5.54
SlCPK3	Solyc08g008170	****MGNCNSLSSDQ	57.703	6.12
SlCPK4-1	Solyc06g065380	QFGTTYQCTEKATGL	57.138	6.04
SlCPK4-2	Solyc11g018610	VSSSTKSCMVLPYQ	56.890	5.61
SlCPK6-1	Solyc10g074570	***MGNTCRGSIGGK	62.253	5.88
SlCPK6-2	Solyc01g112250	***MGNACRGSFGGK	59.918	5.95
SlCPK7-1	Solyc10g079130	***MGNCCAVPKTSE	59.629	6.32
SlCPK7-2	Solyc11g065660	***MGNCCGTPGNSS	59.654	6.45
SlCPK9	Solyc07g064610	****MGICASKGKPN	57.818	7.05
SlCPK10	Solyc03g113390	****MGNCNACIRPE	60.936	6.86
SlCPK11-1	Solyc04g049160	***************	57.231	5.24
SlCPK11-2	Solyc05g056570	QFGTTYLCTEISTSN	56.430	5.13
SlCPK13	Solyc09g005550	***MGNCCRSPAAVA	59.620	6.45
SlCPK16	Solyc02g083850	***MGNICFSSSKVS	64.204	9.26
SlCPK17-1	Solyc11g064900	****MGNCCSRGQPN	59.527	6.13
SlCPK17-2	Solyc01g008740	***MGNCCSSGEEQQ	61.102	5.75
SlCPK20-1	Solyc10g076900	***MGTNCSGPTLNK	55.615	5.48
SlCPK20-2	Solyc01g006730	***MGNTCIGPKLGN	64.616	6.11
SlCPK20-3	Solyc10g081640	***MGNNCVGPKLAN	63.522	5.15
SlCPK21	Solyc03g031670	****MGGCFSKKYTQ	62.991	6.78
SlCPK24	Solyc06g073350	****MGTCMSQNAS	61.061	6.05
SlCPK28	Solyc03g033540	****MGSCFSSSKVS	63.883	8.93
SlCPK29	Solyc04g081910	GLCFTKECCCPHYRD	58.923	5.45
SlCPK32	Solyc01g008440	***MGNCCVKPGKSA	59.987	7.02
SlCPK34	Solyc12g099790	***MGGCCSKAETDP	59.625	5.61
Solanum tuberosum				
StCPK1-1	PGSC0003DMG400000994	***MGNTCVGPSISK	64.801	5.54
StCPK1-2	PGSC0003DMG400028229	***MGNNCVHSKFTK	68.347	5.86
StCPK2	PGSC0003DMG400027527	RVVPALICETLLTNE	57.665	5.37
StCPK3	PGSC0003DMG400005829	****MGNCNSLSSDH	57.847	6.19
StCPK4	PGSC0003DMG400026077	QFGTTYQCTEKVTGL	56.453	6.04
StCPK6	PGSC0003DMG400016820	***MGNTCRGSIGGK	63.3.66	5.88
StCPK8	PGSC0003DMG400010704	***MGNCCVKPGKSA	60.004	6.91
StCPK9-1	PGSC0003DMG400027877	****MGVCLSKSKPA	56.625	6.50
StCPK9-2	PGSC0003DMG400022318	****MGICVSKGKPN	57.862	7.19

StCPK11-1	PGSC0003DMG400023440	QFGTTYLCTEISTSN	56.374	5.13
StCPK11-2	PGSC0003DMG400000890	VSSSTKSCMNVLPYQ	56.996	5.76
StCPK16-1	PGSC0003DMG400022562	****MGSCFSSSKVS	64.172	8.92
StCPK16-2	PGSC0003DMG400003564	***MGNICFSSSKVS	63.596	9.30
StCPK17-1	PGSC0003DMG400004646	***MGGCCSKAETDP	59.662	5.68
StCPK17-2	PGSC0003DMG400009451	***MGNCCSRGQPND	58.970	6.16
StCPK20-1	PGSC0003DMG401007209	****MGNTCSGPTLNK	60.369	5.49
StCPK20-2	PGSC0003DMG401028133	***MGNNCVGPKLAN	70.172	5.31
StCPK20-3	PGSC0003DMG400021342	***MGNTCIGPKLGN	64.626	6.06
StCPK24	PGSC0003DMG400026908	****MGTCMSVQNAS	61.050	6.40
StCPK29	PGSC0003DMG400009883	GLCFTKECCCPHYRD	60.960	5.58
StCPK32	PGSC0003DMG400008149	***MGNCCAVPKTSE	59.424	6.36
Sorghum bicolor				
SbCPK1-1	Sb01g005780	***MGNTCVGPSITK	67.531	6.33
SbCPK1-2	Sb08g014910	***MGNVCVGSRFSK	71.779	5.52
SbCPK2-1	Sb01g005750	***MGNTCVGPSAAG	64.189	5.50
SbCPK2-2	Sb02g003500	***MGNTCVGPSTPS	63.946	5.97
SbCPK3-1	Sb03g028340	***************	59.310	6.38
SbCPK3-2	Sb09g029950	**************	61.089	6.94
SbCPK4	Sb05g004610	QFGTTYQCVGKADGA	57.098	5.60
SbCPK5	Sb04g031570	QFGTTYLCTDLATGV	54.508	5.91
SbCPK6-1	Sb06g026530	***MGNACGGSLRSK	61.166	5.72
SbCPK6-2	Sb01g048570	***MGNQCPNGTLGS	61.647	5.38
SbCPK7	Sb08g007660	***MGNCCVTPKGAA	61.181	7.59
SbCPK8-1	Sb02g036730	***MGNCCAAPLTEE	60.000	6.81
SbCPK8-2	Sb01g004150	***MGNCCVARPLSG	63.269	6.70
SbCPK9	Sb02g034640	***MGQCCSRATAPD	59.521	6.30
SbCPK11	Sb08g004510	QFGTTYQCVGKADGA	56.727	5.66
SbCPK13-1	Sb09g022960	***MGNCCRSPAAAA	60.460	6.77
SbCPK13-2	Sb03g038870	***MGNCCRSPAAVA	60.728	6.60
SbCPK13-3	Sb07g025560	GCYSAFACSRKLRGR	63.120	6.80
SbCPK16	Sb02g009790	****MGLCSSCTAAR	57.594	7.77
SbCPK17-1	Sb09g024100	***MGNCCCSGSSSA	58.061	5.89
SbCPK17-2	Sb05g002110	****MGQCCSKGTGE	58.845	6.37
SbCPK17-3	Sb08g001380	**MSVAACPPSSEQS	62.537	6.27
SbCPK26	Sb04g038450	***MGNSCRGSSSSS	63.983	5.95
SbCPK28	Sb04g002220	****MGACFSSSASA	58.788	8.54
SbCPK29	Sb06g025220	****MGNCFTKTHEQ	59.701	6.33

SbCPK30	Sb01g011630	***MGNVCFCGTTST	64.906	8.75
SbCPK32	Sb03g043700	****MGGCYSVIAAT	62.649	6.85
SbCPK34	Sb03g037570	QFGVTSLCTHKATGE	51.930	5.72
Thellungiella halophila				
ThCPK1	Thhalv10012990m.g	***MGNTCVGPSRNG	68.533	5.51
ThCPK2	Thhalv10020268m.g	***MGNACVGPNISG	72.069	5.52
ThCPK3	Thhalv10024899m.g	ETKKLFACKSIPTRR	59.207	6.46
ThCPK4	Thhalv10028598m.g	SSTANYACKSIPKRK	56.471	5.32
ThCPK5	Thhalv10024803m.g	***MGNSCRRGSFRER	63.251	5.56
ThCPK6	Thhalv10022628m.g	***MGNSCRGSFKDK	62.259	5.44
ThCPK7	Thhalv10013170m.g	***MGNCCGNPSSAT	61.668	5.51
ThCPK8	Thhalv10013210m.g	***MGNCCASPGSDT	60.056	6.31
ThCPK9	Thhalv10020475m.g	****MGNCFAKNHGL	59.999	6.33
ThCPK10	Thhalv10007293m.g	*MGNCNVCVRPDSED	61.648	6.23
ThCPK11	Thhalv10001816m.g	STSANYACKSIPKRK	56.221	5.55
ThCPK12	Thhalv10004083m.g	QFGTTFLCTHNQTGQ	55.512	5.36
ThCPK13	Thhalv10010284m.g	***MGNCCRSPAAVA	59.405	6.68
ThCPK14	Thhalv10016499m.g	***MGNCCGTAGSLI	60.285	7.79
ThCPK15	Thhalv10027200m.g	***************	37.808	5.08
ThCPK16	Thhalv10022615m.g	****MGLCFSSAKVS	65.746	9.31
ThCPK17	Thhalv10013236m.g	****MGNCCSHGRDS	58.525	6.29
ThCPK18	Thhalv10027243m.g	****MGLCFSSPKAT	58.824	8.42
ThCPK19	Thhalv10023804m.g	***MGCLCINLKKKL	63.071	6.67
ThCPK20	Thhalv10017647m.g	***MGNTCVGPNLNP	64.906	5.86
ThCPK21	Thhalv10028567m.g	*****MGCFSSKHRE	60.499	6.71
ThCPK22	Thhalv10028617m.g	*****MGCCGSKNLP	54.532	5.45
ThCPK23	Thhalv10026767m.g	***NTYACKSILKRK	43.254	5.79
ThCPK24	Thhalv10016430m.g	****MGSCVSSPLKG	66.040	7.16
ThCPK26	Thhalv10025058m.g	HNGGNQACFVLGQKT	54.243	5.75
ThCPK28	Thhalv10003969m.g	****MGVCFSAIRVT	60.524	8.80
ThCPK29	Thhalv10018359m.g	NIRTKHFCKINIMGF	62.962	7.61
ThCPK30	Thhalv10018366m.g	****MGNCIACVRFD	61.999	7.14
ThCPK31	Thhalv10028618m.g	KFSITRKCVEKSTGK	54.622	5.11
ThCPK32	Thhalv10005884m.g	***MGNCCGTAGSLA	60.346	6.51
ThCPK34	Thhalv10013240m.g	***MGNCCSHGRDSR	58.384	6.36
Theobroma cacao				
TcCPK1	Thecc1EG044380	***MGNSCVGPSISK	64.988	5.80
TcCPK2	Thecc1EG023099	***MGNSCITLRKDG	68.088	5.54

TcCPK3	Thecc1EG016367	****MGNCNGHPSAD	60.033	6.46
TcCPK4	Thecc1EG001677	QFGTTYLCTHKPTGQ	55.383	5.40
TcCPK6	Thecc1EG007580	***MGNTCRGSFKGK	63.005	5.90
TcCPK7	Thecc1EG024724	***MGNCCATPSTG	60.263	6.91
TcCPK8	Thecc1EG040717	***MGNCCATPGSPV	59.895	7.02
TcCPK11	Thecc1EG036367	QFGTTYQCTHKATGT	63.958	6.26
TcCPK13	Thecc1EG021684	***MGNCCRSPAAVA	62.325	6.50
TcCPK21	Thecc1EG030220	*****MGCCGSKHKL	60.576	6.50
TcCPK24	Thecc1EG020113	****MGSCISTPSRL	61.186	6.07
TcCPK28	Thecc1EG000206	****MGACLSTTKVS	69.011	8.58
TcCPK29-1	Thecc1EG034291	****MGLCQSLGFCL	58.274	5.59
TcCPK29-2	Thecc1EG034289	****MGLCQSQGFCL	59.705	6.62
TcCPK30	Thecc1EG013784	MGNCNACVRPDDSP	62.313	6.51
TcCPK33	Thecc1EG030876	****MGGCLTKSKEP	60.518	6.70
TcCPK34	Thecc1EG040893	****MGNCCSRGNPA	59.764	6.34
Vitis venifera				
VvCPK1	GSVIVG01001931001	**************	38.703	4.88
VvCPK2	GSVIVG01022606001	***MGNNCVGSMVPE	63.727	5.39
VvCPK3	GSVIVG01019446001	ERAAAALCRQIVTVV	45.355	5.92
VvCPK4	GSVIVG01034489001	******************	39.061	4.91
VvCPK6	GSVIVG01023866001	***MGNTCRGSFRGK	51.999	5.50
VvCPK8	GSVIVG01025249001	***MGNCCASPGSEK	58.575	6.40
VvCPK9	GSVIVG01037652001	*****************	37.228	5.11
VvCPK10	GSVIVG01008077001	****MELCEGGELFD	46.130	5.37
VvCPK12	GSVIVG01000238001	******************	38.990	4.88
VvCPK13	GSVIVG01011167001	GRGEFGYCELEGGLC	43.128	5.69
VvCPK17	GSVIVG01037295001	****MGNCCSQGNTN	55.858	5.66
VvCPK20	GSVIVG01022524001	***MGNTCVGPNLAA	61.312	5.34
VvCPK21	GSVIVG01012730001	*****MGCFSSKERV	58.339	6.55
VvCPK24	GSVIVG01010743001	TNNPMGGCISMPAKA	65.686	6.78
VvCPK28	GSVIVG01018778001	****MGACLSATKVR	62.989	9.04
VvCPK29	GSVIVG01008749001	****MGFCFSRPRDI	58.954	5.45
VvCPK32	GSVIVG01033306001	****MELCEGGELFD	44.853	6.11
Volvox carteri				
VcCPK4	Vocar20014761m.g	*****MGCSSSKDGY	102.653	7.40
VcCPK12	Vocar20008501m.g	EGRMGNLCSCVGEAP	56.541	6.31
VcCPK15	Vocar20001731m.g	VHLVMDLCQGDLYEL	57.540	5.31
VcCPK17	Vocar20000362m.g	ISKAKLVCKEDVKDV	54.201	6.40

VcCPK20	Vocar20014333m.g	SFGVVRECVEKSSGR	72.458	6.75
VcCPK34	Vocar20005450m.g	****MGSCASTENQV	55.583	6.19
Zea mays				
ZmCPK1-1	GRMZM2G028926_T01	***MGNTCVGPSITK	66.414	6.13
ZmCPK1-2	GRMZM2G121228_T01	***MGNTCVGPSAAG	63.696	5.44
ZmCPK1-3	GRMZM2G027351_T01	*** MGNTCVGPSAAG	64.113	5.39
ZmCPK1-4	GRMZM2G353957_T01	***MGNVCVGPRFSK	71.678	6.19
ZmCPK1-5	GRMZM2G117796_T01	****MGLCYGKSAAV	55.320	8.78
ZmCPK2	GRMZM2G320506_T01	***MGNTCVGPSITM	67.731	5.89
ZmCPK3-1	GRMZM2G058305_T01	********************	60.731	6.93
ZmCPK3-2	GRMZM2G025387_T01	********************	52.461	5.75
ZmCPK3-3	GRMZM5G856738_T02	ETGQRFACKSIATRK	52.461	5.75
ZmCPK4-1	GRMZM2G347226_T01	QFGTTYQCVGKADGA	56.761	5.83
ZmCPK4-2	GRMZM2G035843_T01	QFGTTYHCVGKADGA	56.479	5.65
ZmCPK4-3	GRMZM2G463464_T01	QFGTTYQCVGKADGG	56.842	5.66
ZmCPK4-4	GRMZM2G047486_T01	QFGTTYQCVGKADGA	56.560	5.59
ZmCPK5-1	GRMZM2G321239_T01	***MGNACGGALRSK	61.239	5.98
ZmCPK5-2	GRMZM2G314396_T01	***MGNACSGALRSK	60.360	5.88
ZmCPK5-3	GRMZM2G347047_T01	QFGTTYLCTELSTGA	53.856	5.69
ZmCPK5-4	GRMZM2G040743_T01	***MGNQCQNGTYGY	60.850	5.17
ZmCPK6-1	GRMZM2G081310_T01	***MGNTCGVTLRSK	61.898	5.91
ZmCPK6-2	GRMZM2G032852_T02	***MGNQCPNGTLGS	61.651	5.43
ZmCPK7	GRMZM2G097533_T01	***MGNCCVSRPSSG	59.102	7.25
ZmCPK8-1	AC233871.1_FGT003	***MGNCCATPSTTE	60.296	6.95
ZmCPK8-2	GRMZM2G104125_T01	******************	40.804	6.32
ZmCPK8-3	GRMZM5G839017_T02	******************	40.751	6.65
ZmCPK8-4	GRMZM5G874665_T02	NDYFVHRCVVEFPDA	55.402	5.61
ZmCPK8-5	GRMZM2G006404_T01	******************	56.564	5.98
ZmCPK8-6	GRMZM2G047479_T01	******************	40.804	6.32
ZmCPK9-1	GRMZM2G154489_T01	***MGQCCSRATAPD	59.365	6.25
ZmCPK9-2	GRMZM2G168706_T01	*** MGQCCSRATAPD	59.352	6.30
ZmCPK13-1	GRMZM2G311220_T01	***MGNCCRSPAAVA	60.168	6.55
ZmCPK13-2	GRMZM2G088361_T01	***MGNCCRSPAAAA	60.217	6.77
ZmCPK13-3	GRMZM2G030673_T01	***MGNCCRSPAAAA	60.492	6.66
ZmCPK16-1	GRMZM2G053868_T01	****MGACFSSASAA	58.471	8.65
ZmCPK16-2	GRMZM2G157068_T01	****MGACFSSASAA	58.471	8.65
ZmCPK17-1	GRMZM2G340224_T01	KLDASFCCFSLLSIN	67.488	7.16
ZmCPK17-2	GRMZM2G365815_T01	****MGQCCSKGAGE	59.977	6.21

ZmCPK17-3	AC203294.3_FGT001	***MGNC**C**PGSGDAE	50.903	6.31
ZmCPK19	GRMZM2G028086_T01	***MGNC**C**VTPKAAD	60.806	7.59
ZmCPK20	GRMZM2G012326_T01	***MGNT**C**IGPSAPS	75.556	6.50
ZmCPK24	GRMZM2G332660_T01	GCYSAFA**C**SRKLRGR	62.778	7.13
ZmCPK28	GRMZM2G365035_T01	****MGL**C**SSSTAAR	57.534	8.37
ZmCPK29	GRMZM2G112057_T01	****MGN**C**FTRKYDE	61.042	6.13
ZmCPK30	GRMZM2G076634_T01	VILYILL**C**GVPPFWA	36.578	6.23
ZmCPK32-1	AC210013.4_FGT014	***MGNC**C**AAPSTEG	60.438	6.73
ZmCPK32-2	GRMZM2G099425_T01	***MGNC**C**VTPNGAA	60.795	6.74
ZmCPK32-3	GRMZM2G158721_T01	LKMLRRG**C**RGAAAVL	74.111	7.95
ZmCPK34-1	GRMZM2G167276_T01	***MGNC**C**PGSGDAE	56.154	6.17
ZmCPK34-2	GRMZM2G472311_T01	****MGQ**C**CSKGQCC	63.392	6.10
