# Supplementary material for: Genome-Wide Identification of Calcium Dependent Protein Kinase Gene Family in Plant Lineage Shows Presence of Novel D-x-D and D-E-L Motifs in EF-Hand Domain
Source: Front Plant Sci. 2015 Dec 24;6:1146. doi: 10.3389/fpls.2015.01146 (PMC4690006; doi:10.3389/fpls.2015.01146)
Supplement: Supplementary file 4 [file Table4.PDF]

Supplementary Table 4

Comparison of average abundance of different amino acids in plant CPK proteins. The average abundance of Leu is highest (8.47/CPK gene) and Trp is the lowest (0.91/CPK gene). The most important amino acids responsible for Ca^{2+} ion binding in the EF-hand [Glue (E) and Asp (D)] have average frequency of 7.83 and 7.14/CPK protein respectively.

Amino Acids	Monocot	Dicot	Lower Eukaryote	All Three
Ala	8.67	6.83	10.08	7.47
Cys	1.42	1.39	1.18	1.39
Asp	7.22	7.16	6.65	7.14
Glu	7.54	8.02	6.97	7.83
Phe	3.84	4.07	3.38	3.97
Gly	7.55	6.96	8.88	7.23
His	2.66	2.72	2.63	2.70
Ile	5.25	6.03	4.17	5.73
Lys	6.53	7.72	5.82	7.31
Leu	8.63*	8.37*	8.84*	8.46*
Met	2.91	3.06	2.54	2.99
Asn	3.25	3.80	2.98	3.62
Pro	4.82	4.25	4.96	4.41
Gln	2.98	3.22	3.85	3.21
Arg	6.32	5.44	6.16	5.69
Ser	6.12	6.37	7.09	6.37
Thr	3.90	4.32	4.38	4.23
Val	6.72	6.53	6.61	6.57
Trp	0.91*	0.91*	0.88*	0.91*
Tyr	2.62	2.73	2.15	2.66
