# Supplementary material for: Genome-Wide Identification of Calcium Dependent Protein Kinase Gene Family in Plant Lineage Shows Presence of Novel D-x-D and D-E-L Motifs in EF-Hand Domain
Source: Front Plant Sci. 2015 Dec 24;6:1146. doi: 10.3389/fpls.2015.01146 (PMC4690006; doi:10.3389/fpls.2015.01146)
Supplement: Supplementary file 5 [file Table5.PDF]

Supplementary Table 5

Presence of different conserved regions in kinase domain of CPK proteins. Domains conserved between monocot, dicot and lower eukaryotic plants are indicated separately. Results indicate the presence of slightly different conserved region in the kinase domain between the lower and higher eukaryotic plants.

**Conserved Sequences of Kinase domain of Plant CPKs**												
Monocots		C-x-G-G-E-L-x-D-R-I	H-R-D-L-K-P-E-N-F/A		D-x-V-G-S-x-Y-Y	A-P-E-V-L	D-I/V-W-S	G-V-I-x-Y-I-L-L	G-x-P-P-F-W	P-W-P-x-I-S	A-K-D-L-V	H-P-W
Dicots		C-x-G-G-E-L-x-D-R-I	H-R-D-L-K-P-E-N-F-L	D-F-G-L-S-x-F	D-x-V-G-S-x-Y-Y	A-P-E-V-L	D-V/I-W-S	G-V-I-x-Y-I-L-L	G-x-P-P-F-W	P-W-P-x-I-S	A-K-D-L-V	H-P-W
Lower Eukaryotes		M-E-L-C-x-G-G-E-L-F	H-R-D-L-K-P-E-N-F-L	D-F-G-L-S-V/x		A-P-E-V-L/x	D-I-W-S-x-G-V		P-F-W	H-P-W		
Altogether	G-Q-G-Q-F-G	V-M-E-x-C-x-G-G-E-L-F-D/x-x-I	G-V-M/x-H-R-D-L-K-P-E-N-F-L	D-F-G-L-S-x-F	D-x-V-G-S-x-Y-Y	A-P-E-V-L	D-V-W-S	G-V-I-x-Y-I-L-L	P-P-F-W	P-W-P-x-I-S	A-K-D-L/V	H-P-W
