# Supplementary material for: Genome-Wide Identification of Calcium Dependent Protein Kinase Gene Family in Plant Lineage Shows Presence of Novel D-x-D and D-E-L Motifs in EF-Hand Domain
Source: Front Plant Sci. 2015 Dec 24;6:1146. doi: 10.3389/fpls.2015.01146 (PMC4690006; doi:10.3389/fpls.2015.01146)
Supplement: Supplementary file 6 [file Table6.PDF]

Supplementary Table 6

Table showing the presence of different conserved regions in auto-inhibitory domain of CPK proteins. Monocot and dicot plants share some common conserved amino acid sequences F-R/S-A-M-N-K-L in the auto-inhibitory domain. However, lower eukaryotic plants only share the A-M-N-K-L domain in the auto-inhibitory domain. The K-T/P-L-D domain is also absent from lower eukaryotic plants.

Conserved amino acid sequences present in Auto-inhibitory domain of Plant CPKs			
Monocots	K/T-P-L-D	F-R/S-A-M-N-K-L	A-L-x-V/I-I-A
Dicots	K-P-L-D	F-S-A-M-N-K-L	A-L-x-x-I-A
Lower Eukaryotes		A-M-N-K-L	
Altogether		S-A-M-N-K-L-K	
