# Supplementary material for: Genome-Wide Identification of Calcium Dependent Protein Kinase Gene Family in Plant Lineage Shows Presence of Novel D-x-D and D-E-L Motifs in EF-Hand Domain
Source: Front Plant Sci. 2015 Dec 24;6:1146. doi: 10.3389/fpls.2015.01146 (PMC4690006; doi:10.3389/fpls.2015.01146)
Supplement: Supplementary file 7 [file Table7.PDF]

Supplementary Table 7

Table shows the presence of D-E-L, E-E-L and some other motifs in different CPKs of monocot and dicot model plants.

Monocot Model plant				
**D-E-L**	**E-E-L**	**x-D/E-L**	**Q-E-L**	**E-E-F/M**
BdCPK17-1	BdCPK3-1	BdCPK6	BdCPK30	BdCPK7-2
OsCPK2	SiCPK3-1	PvCPK6	PvCPK30-1	PvCPK8-2
PvCPK34-1	SbCPK3-1	SbCPK6-2	PvCPK30-2	SiCPK7-1
ZmCPK34-1	OsCPK1	ZmCPK6-2	SiCPK30	SbCPK8-1
BdCPK34-1	BdCPK3-2	SiCPK6-2	SbCPK30	ZmCPK8-1
SiCPK34-1	SbCPK3-2	OsCPK7	OsCPK9	ZmCPK32-1
SbCPK17-1	ZmCPK3-1	SiCPK6-1		OsCPK8
ZmCPK17-1	OsCPK15	ZmCPK5-4		PvCPK7-1
OsCPK14	PvCPK3-1	OsCPK23		BdCPK16-2
SbCPK34	ZmCPK3-2	OsCPK22		OsCPK3
PvCPK34-3	ZmCPK3-3	ZmCPK32-3		OsCPK4
SbCPK17-2	PvCPK3-2			SiCPK16
ZmCPK17-2	PvCPK3-3			SbCPK28
ZmCPK34-2	BdCPK34-3			ZmCPK16-1
PvCPK17-1	BdCPK17-2			ZmCPk16-2
PvCPK17-2	OsCPK25			PvCPK16-2
PvCPK17-3	OsCPK26			
BdCPK12	SbCPK17-3			
OsCPK24	BdCPK33			
PvCPK4-3	OsCPK19			
SbCPK4	PvCPK9-1			
ZmCPK4-4	PvCPK9-2			
ZmCPK4-2	SiCPK9			
PvCPK4-1	SbCPK9			
PvCPK4-2	ZmCPK9-1			
SiCPK4	ZmCPK9-2			
SbCPK11	BdCPK29			
ZmCPK4-3	OsCPK12			
ZmCPK4-1	PvCPK29-1			
OsCPK28	SiCPK29			
BdCPK5-1	SbCPK29			
SiCPK5-3	ZmCPK29			
SbCPK6-1	BdCPK1-1			
ZmCPK5-1	PvCPK1-1			
ZmCPK5-2	SiCPK20			
OsCPK13	SbCPK1-2			
BdCPK5-2	ZmCPK1-4			
SiCPK5-1	OsCPK27			
ZmCPK6-1	BdCPK2			
OsCPK5	PvCPK1-2			

PvCPK5-2	PvCPK1-3			
PvCPK5-3	SiCPK1-1			
PvCPK5-1	SbCPK1-1			
PvCPK5-4	ZmCPK1-1			
SbCPK5	ZmCPK2			
SbCPK26	OsCPK10			
SiCPK5-2	BdCPK20			
ZmCPK5-3	PvCPK1-5			
OsCPK6	PvCPK1-6			
PvCPK4-4	SiCPK1-2			
BdCPK32	SbCPK2-1			
PvCPK8-1	ZmCPK1-3			
PvCPK7-3	ZmCPK1-2			
SiCPK8	OsCPK11			
SbCPK7	BdCPK1-2			
ZmCPK32-2	OsCPK17			
ZmCPK19	PvCPK1-4			
OsCPK20	SiCPK1-3			
SbCPK32	SbCPK2-2			
OsCPK21	ZmCPK20			
SiCPK34-2	BdCPK5-3			
ZmCPK17-3	PvCPK5-5			
PvCPK7-2	PvCPK5-6			
PvCPK4-5	PvCPK9-3			
	BdCPK13-1			
	BdCPK13-2			
	OsCPK16			
	PvCPK13-2			
	PvCPK13-1			
	SiCPK13-1			
	SiCPK13-2			
	ZmCPK13-1			
	ZmCPK13-2			
	ZmCPK13-3			
	SbCPK13-2			
	ZmCPK19			
	BdCPK7-1			
	PvCPK32-3			
	PvCPK32-4			
	SiCPK7-2			
	SbCPK8-2			
	OsCPK29			
	ZmCPK7			
	BdCPK24			
	PvCPK32-5			
	SiCPK13-4			
	PvCPK13-3			

	BdCPK7-3			
	PvCPK32-2			
	SbCPK13-3			
	ZmCPK24			
	SiCPK13-3			
	PvCPK3-4			
	PvCPK29-2			
	BdCPK16-1			
	PvCPk16-1			
	SbCPk16			
	ZmCPK28			
	OsCPK18			
	ZmCPK8-2			
	ZmCPK8-6			
	ZmCPK8-3			
	ZmCPK8-4			
	ZmCPK8-5			
	ZmCPK30			
	SiCPK2			
	PvCPK1-7			
	PvCPK1-8			
	PvCPK4-5			
	PvCPK32-1			
	PvCPK24			
**Dicot Model Plant (*Arabidopsis thaliana*)**				
AtCPK5	AtCPK1			AtCPK16
AtCPK6	AtCPK2			AtCPK28
AtCPK19	AtCPK20			AtCPK18
AtCPK32	AtCPK4			
AtCPK26	AtCPK11			
AtCPK29	AtCPK12			
	AtCPK3			
	AtCPK17			
	AtCPK34			
	AtCPK9			
	AtCPK33			
	AtCPK15			
	AtCPK21			
	AtCPK7			
	AtCPK8			
	AtCPK13			
	AtCPK24			
	AtCPK22			
	AtCPK27			
