# Supplementary material for: Genome-Wide Identification of Calcium Dependent Protein Kinase Gene Family in Plant Lineage Shows Presence of Novel D-x-D and D-E-L Motifs in EF-Hand Domain
Source: Front Plant Sci. 2015 Dec 24;6:1146. doi: 10.3389/fpls.2015.01146 (PMC4690006; doi:10.3389/fpls.2015.01146)

Supplementary Figure 1

Multiple sequence alignment of CPKs of monocot, dicot and lower eukaryotes (algae, bryophytes, pteridophytes). The CPKs of lower eukaryotes are longer than the CPKs of monocot and dicot plants. This indicates that, the size of CPKs gradually got reduced during the course of evolution. Amino acids in red color indicate the 90% consensus level and are conserved throughout the lower eukaryotic plant lineage.

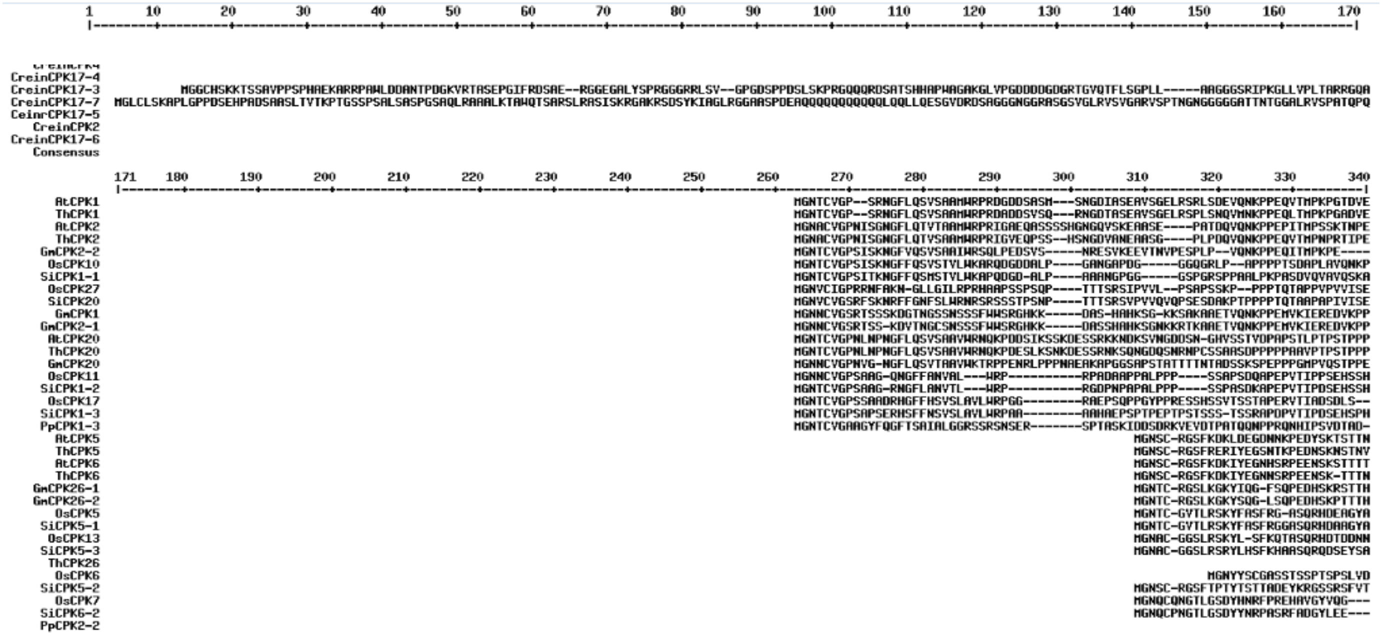

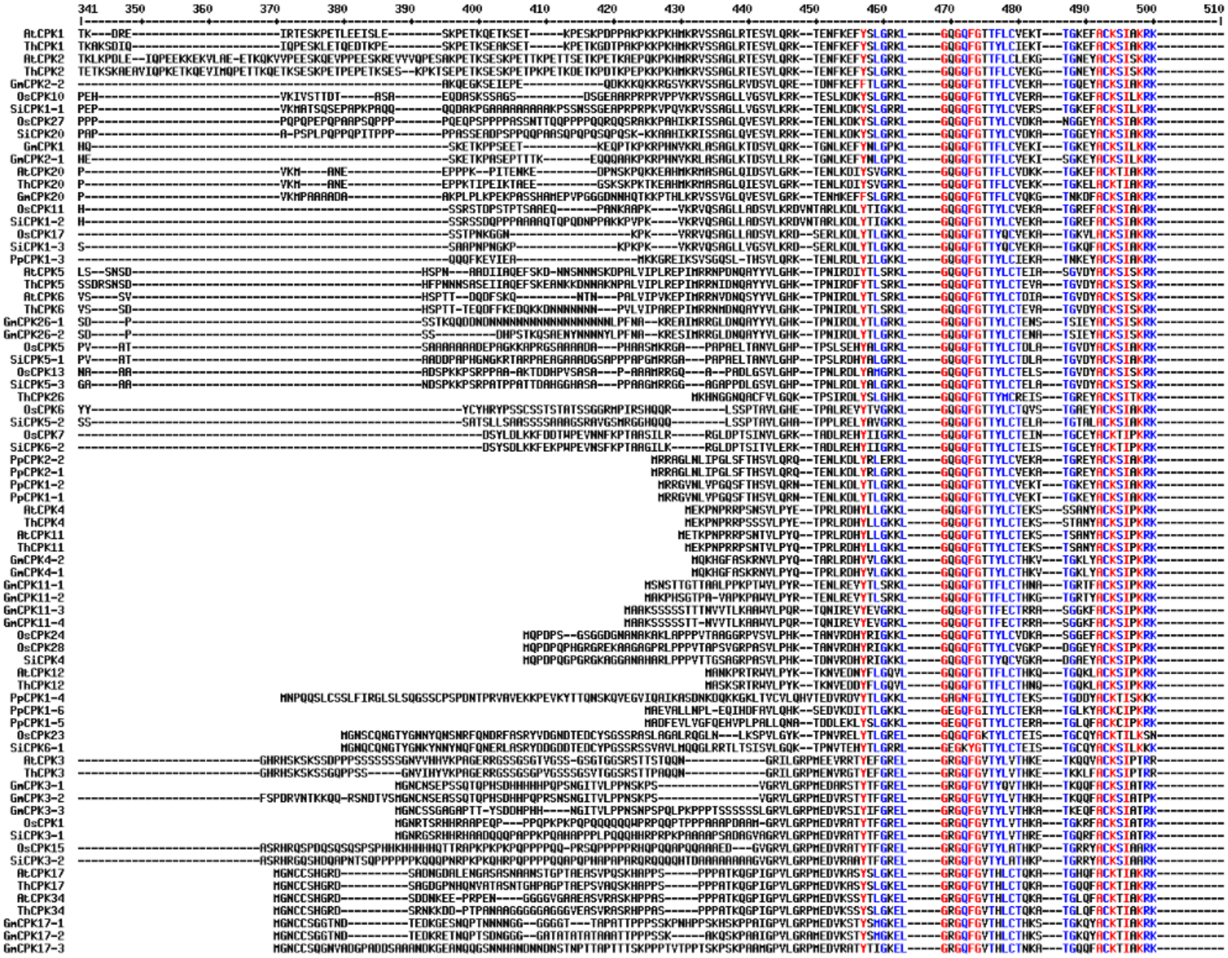
341 350 360 370 380 390 400 410 420 430 440 450 460 470 480 490 500 510
AtCPK1
ThCPK1
AtCPK2
ThCPK2
GmCPK2-2
OsCPK10
SiCPK1-1
OsCPK27
SiCPK20
GmCPK1
GmCPK2-1
AtCPK20
ThCPK20
GmCPK20
OsCPK11
SiCPK1-2
OsCPK17
SiCPK1-3
PpCPK1-3
AtCPK5
ThCPK5
AtCPK6
ThCPK6
GmCPK26-1
GmCPK26-2
OsCPK5
SiCPK5-1
OsCPK13
SiCPK5-3
ThCPK26
OsCPK6
SiCPK5-2
OsCPK7
SiCPK6-2
PpCPK2-2
PpCPK2-1
PpCPK1-2
PpCPK1-1
AtCPK4
ThCPK4
AtCPK11
ThCPK11
GmCPK4-2
GmCPK4-1
GmCPK11-1
GmCPK11-2
GmCPK11-3
GmCPK11-4
OsCPK24
OsCPK28
SiCPK4
AtCPK12
ThCPK12
PpCPK1-4
PpCPK1-6
PpCPK1-5
OsCPK23
SiCPK6-1
AtCPK3
ThCPK3
GmCPK3-1
GmCPK3-2
GmCPK3-3
OsCPK1
SiCPK3-1
OsCPK15
SiCPK3-2
AtCPK17
ThCPK17
AtCPK34
ThCPK34
GmCPK17-1
GmCPK17-2
GmCPK17-3

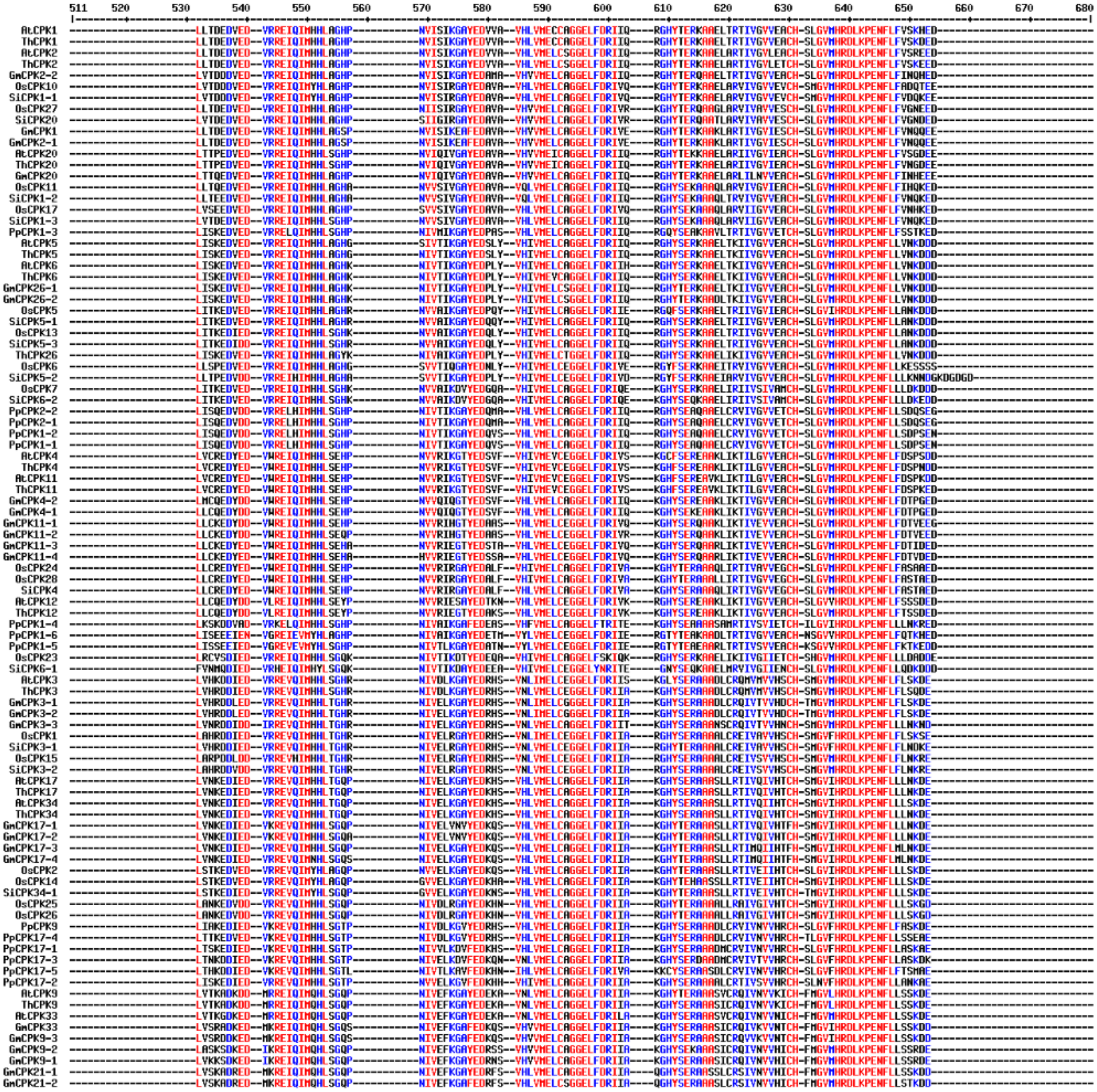

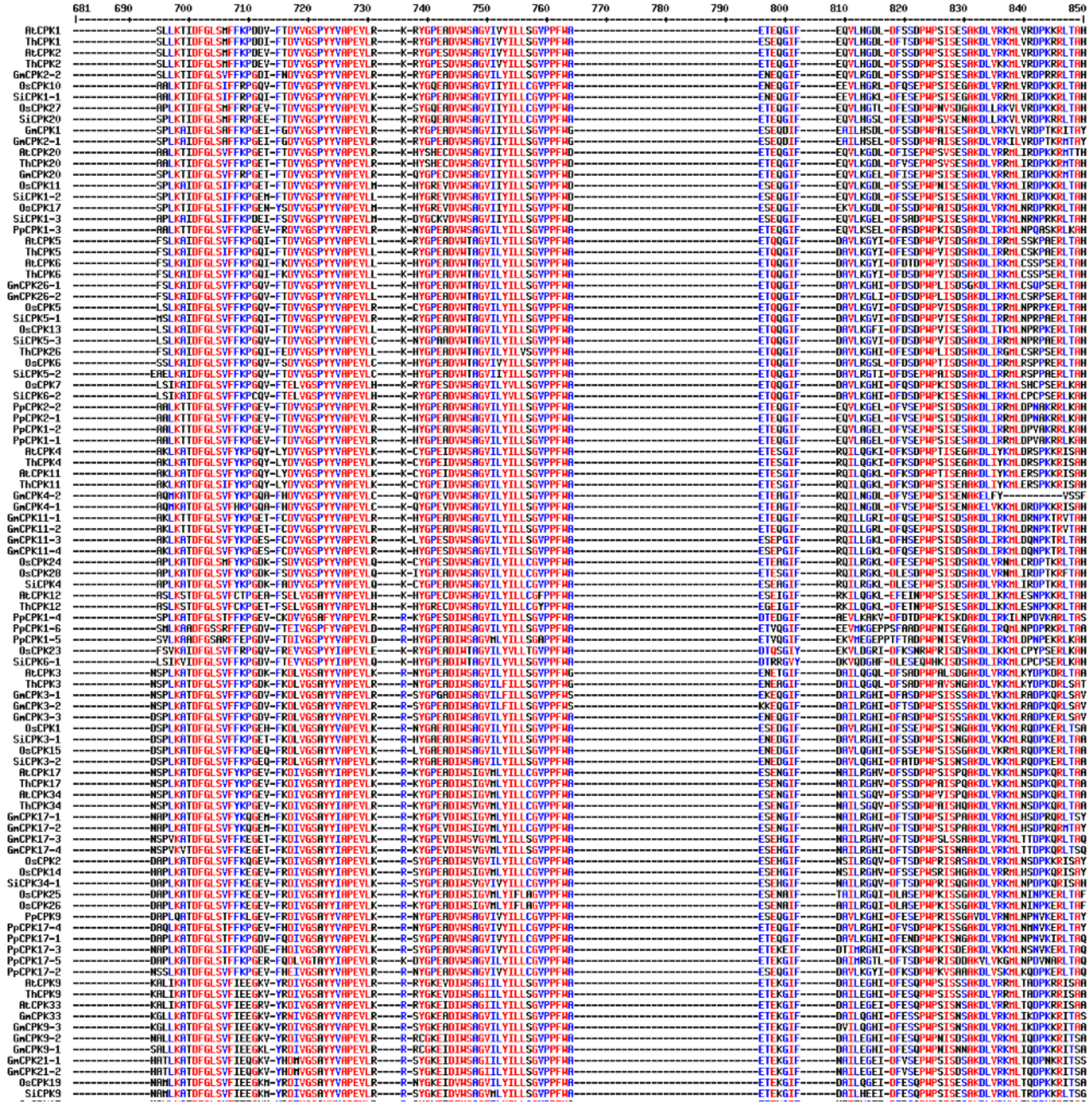

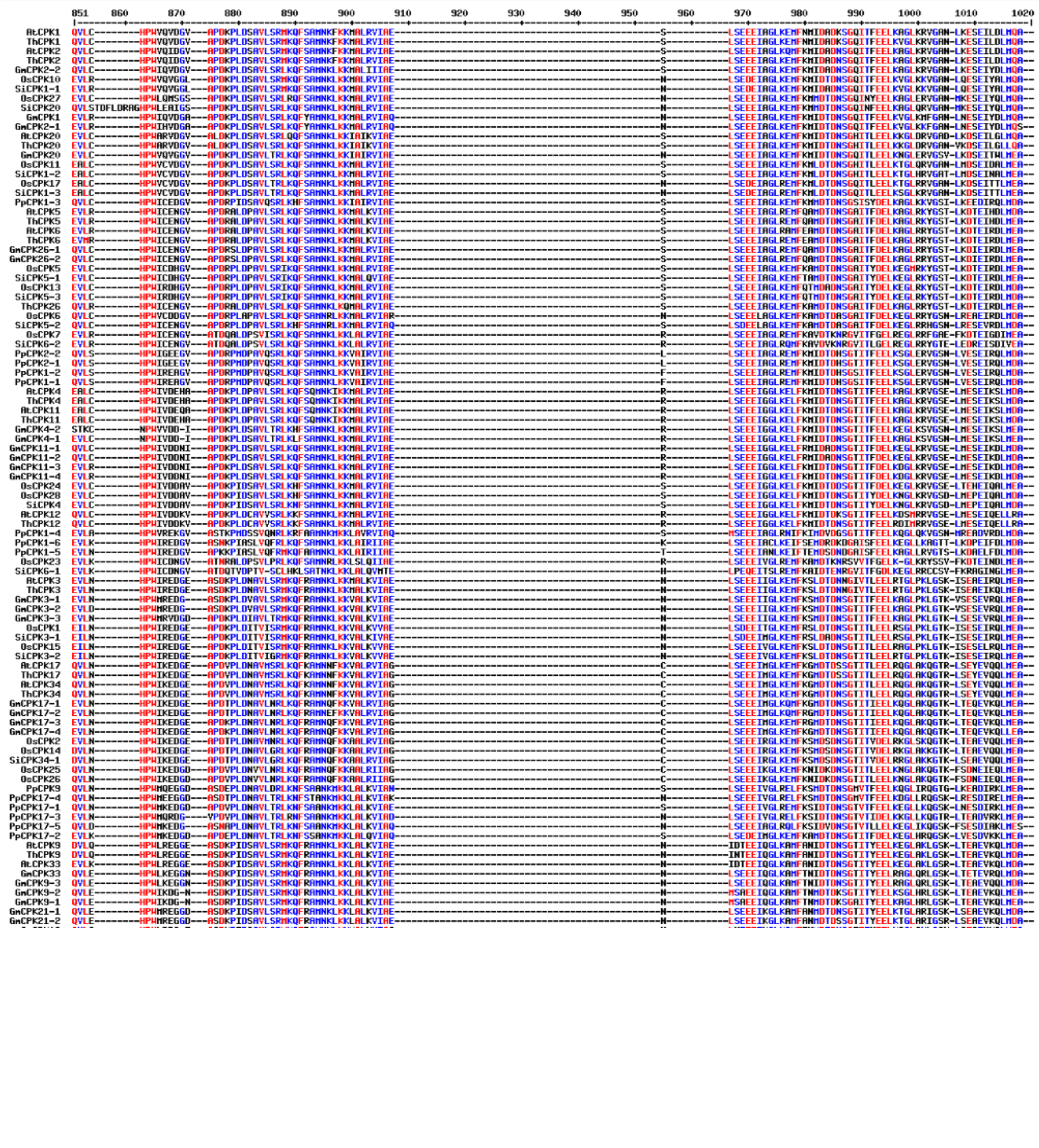

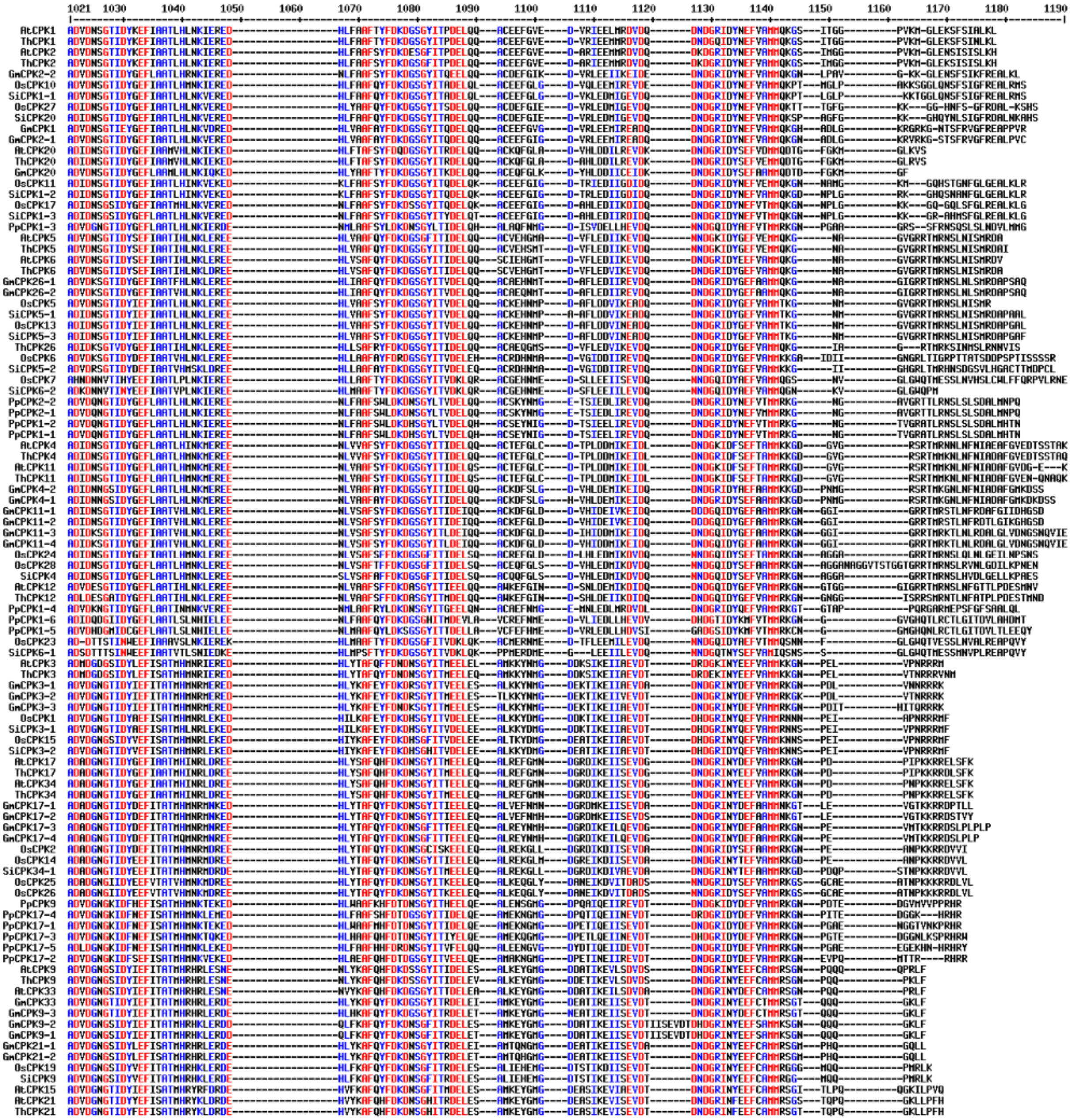

Supplement: Supplementary file 8 [file Image1.PDF]
