# Supplementary material for: Genome-Wide Identification of Calcium Dependent Protein Kinase Gene Family in Plant Lineage Shows Presence of Novel D-x-D and D-E-L Motifs in EF-Hand Domain
Source: Front Plant Sci. 2015 Dec 24;6:1146. doi: 10.3389/fpls.2015.01146 (PMC4690006; doi:10.3389/fpls.2015.01146)
Supplement: Supplementary file 9 [file Image2.PDF]

Supplementary Figure 2

Multiple sequence alignment of CPKs of monocot plants. Amino acids in red indicate the 90% consensus level and are conserved throughout the monocot plant lineage.

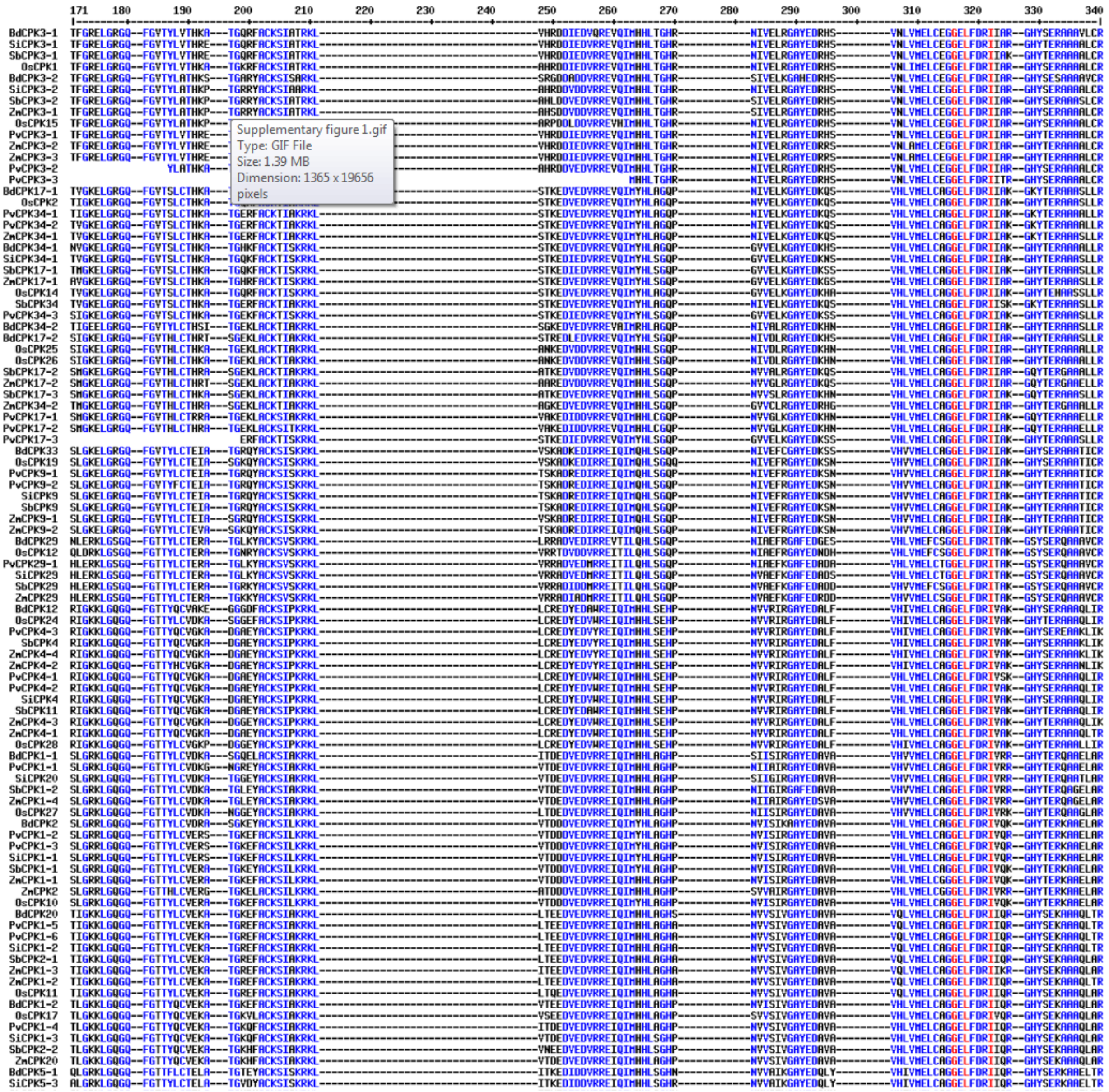

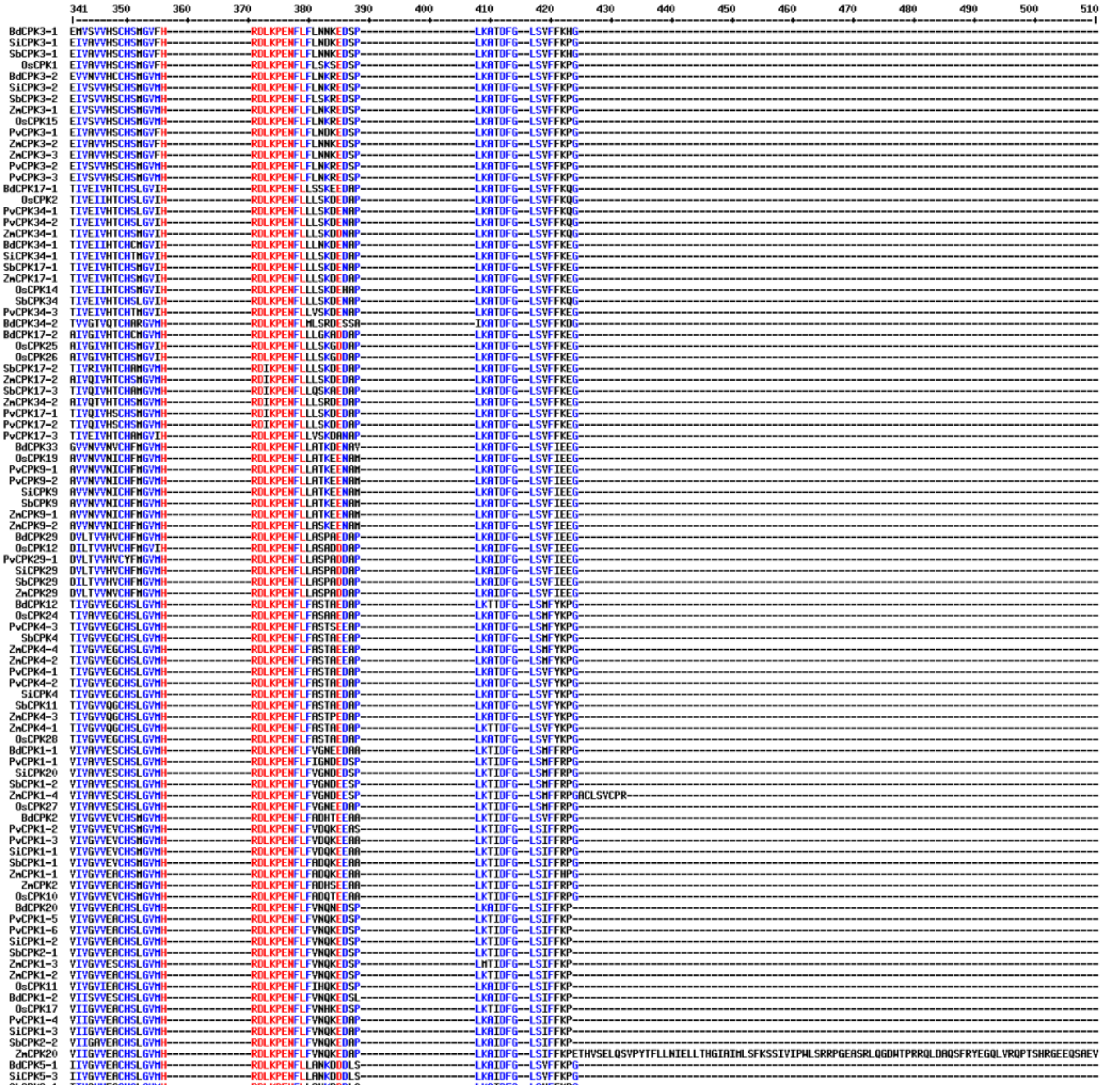

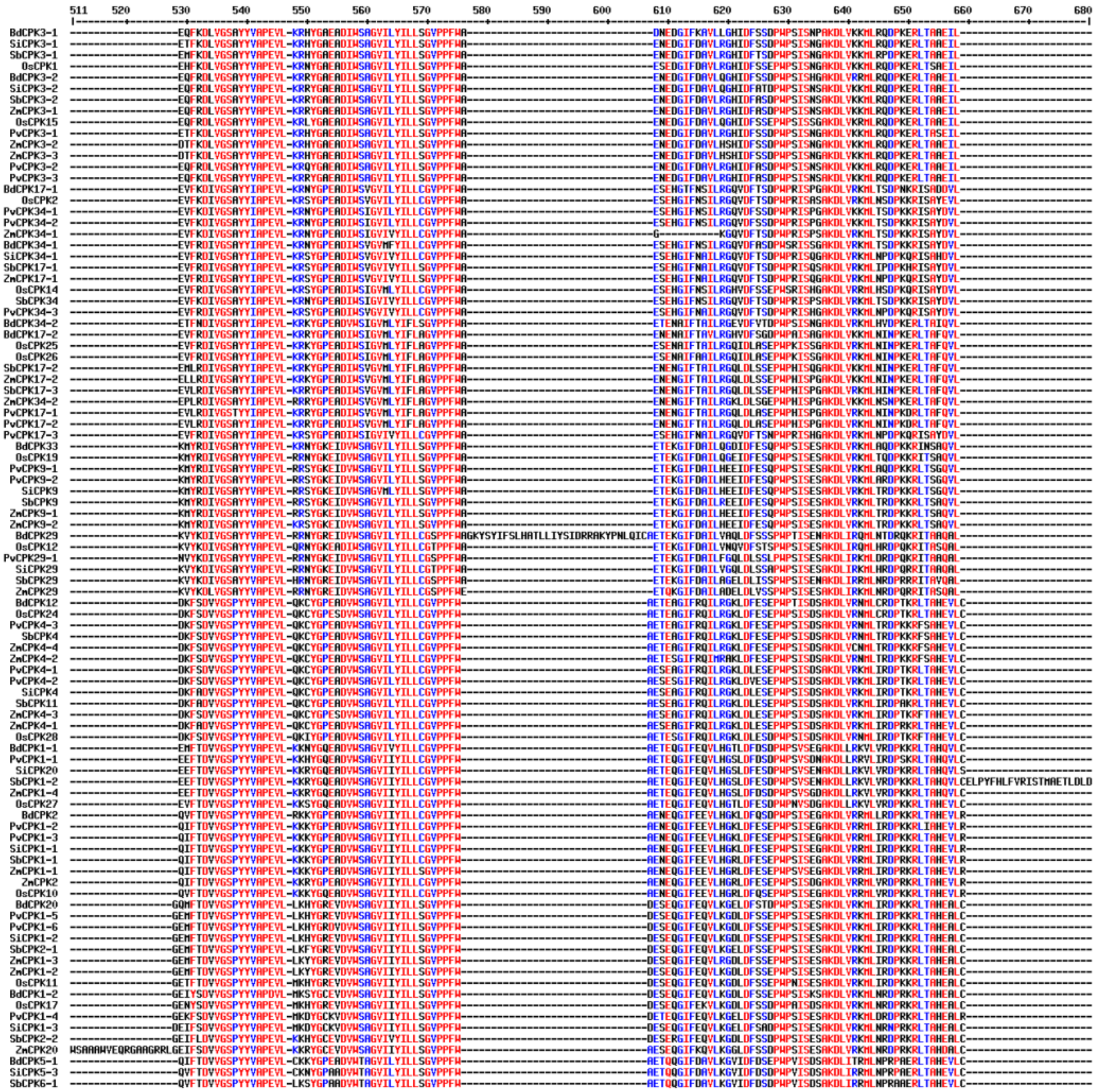

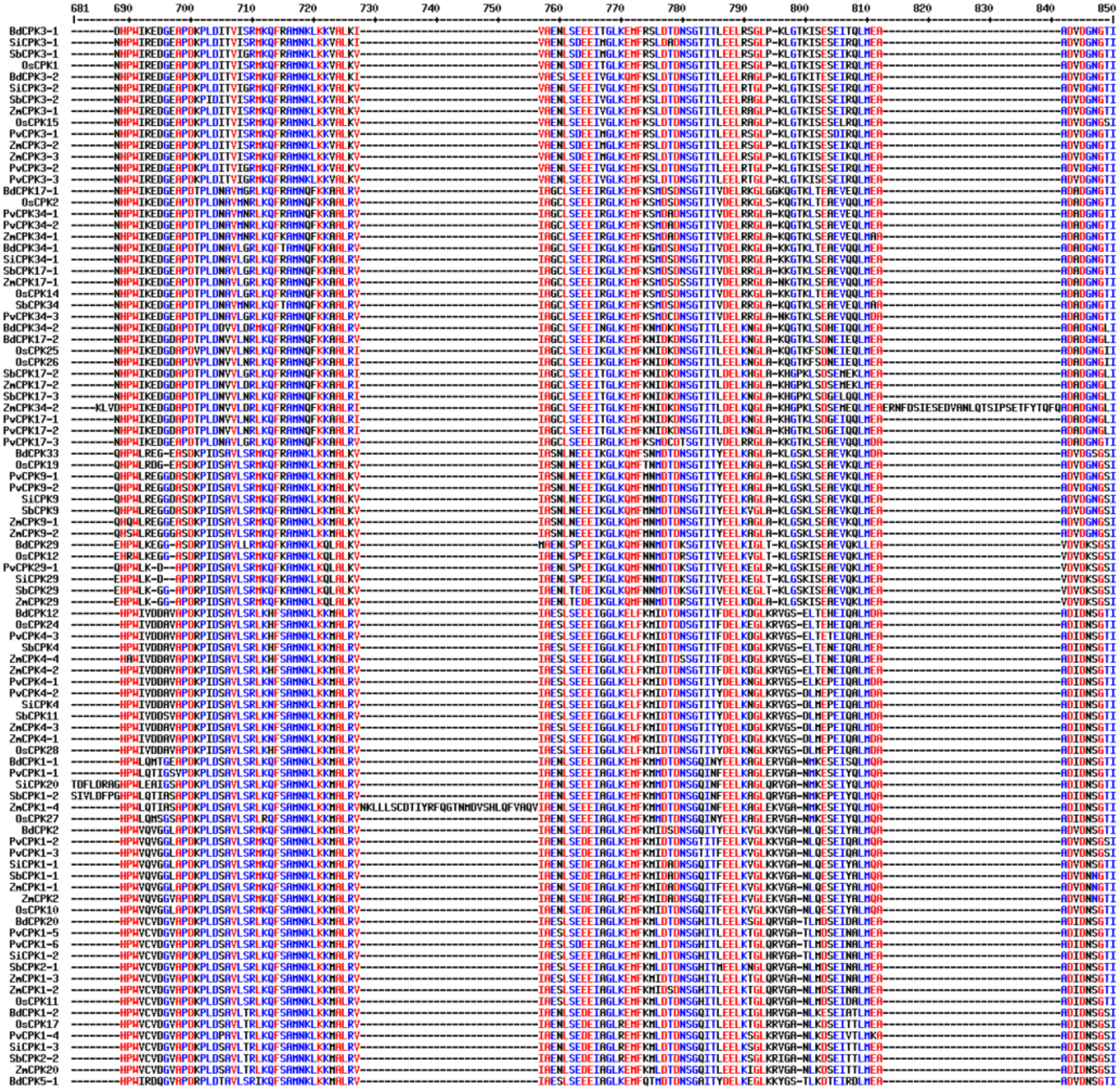

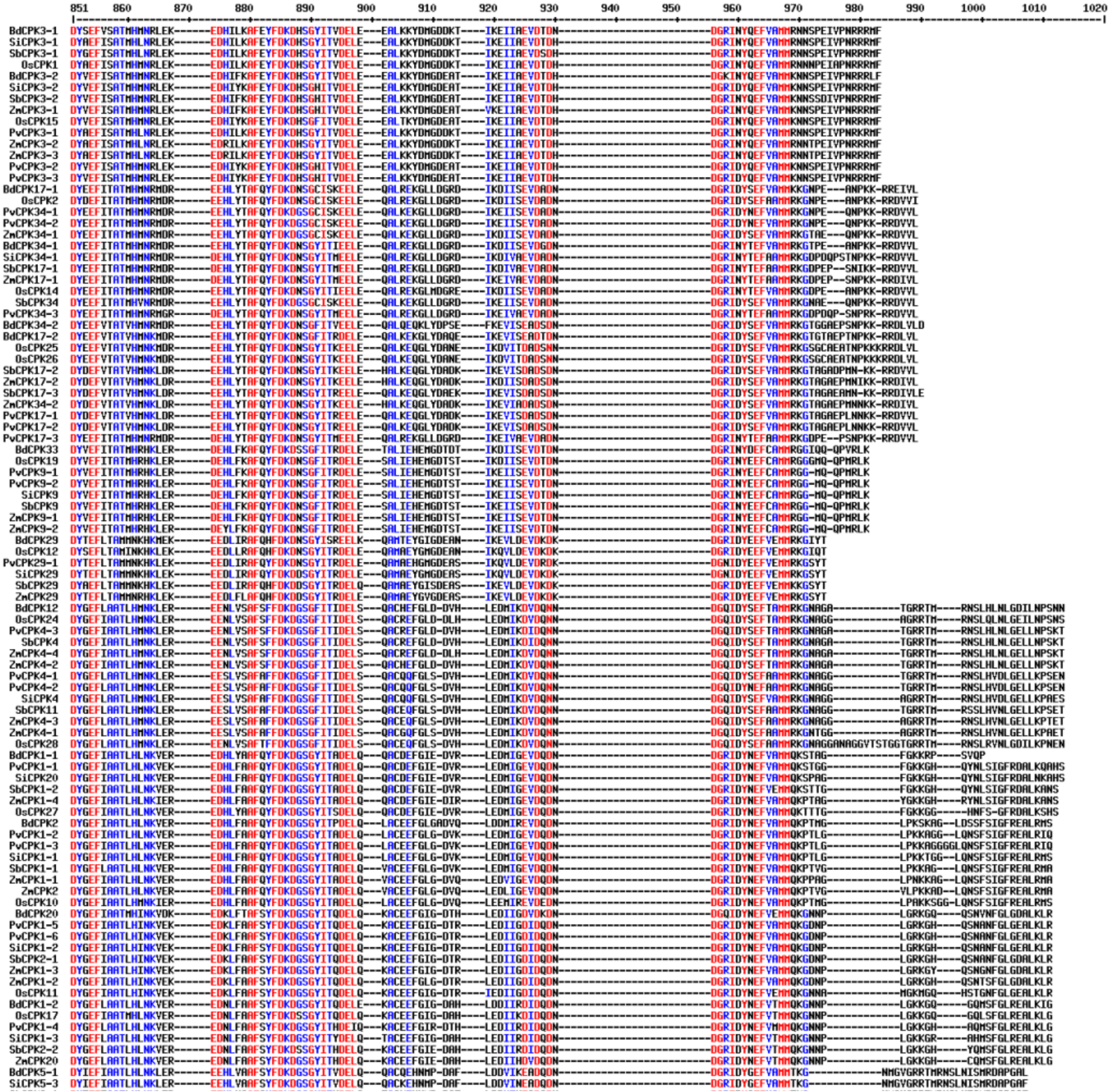
851 860 870 880 890 900 910 920 930 940 950 960 970 980 990 1000 1010 1020
