# Supplementary material for: Genome-Wide Identification of Calcium Dependent Protein Kinase Gene Family in Plant Lineage Shows Presence of Novel D-x-D and D-E-L Motifs in EF-Hand Domain
Source: Front Plant Sci. 2015 Dec 24;6:1146. doi: 10.3389/fpls.2015.01146 (PMC4690006; doi:10.3389/fpls.2015.01146)

Supplementary Figure 3

Multiple sequence alignment of CPKs of dicot plant. Amino acids in red indicate the 90% consensus level and are conserved throughout the dicot plant lineage.

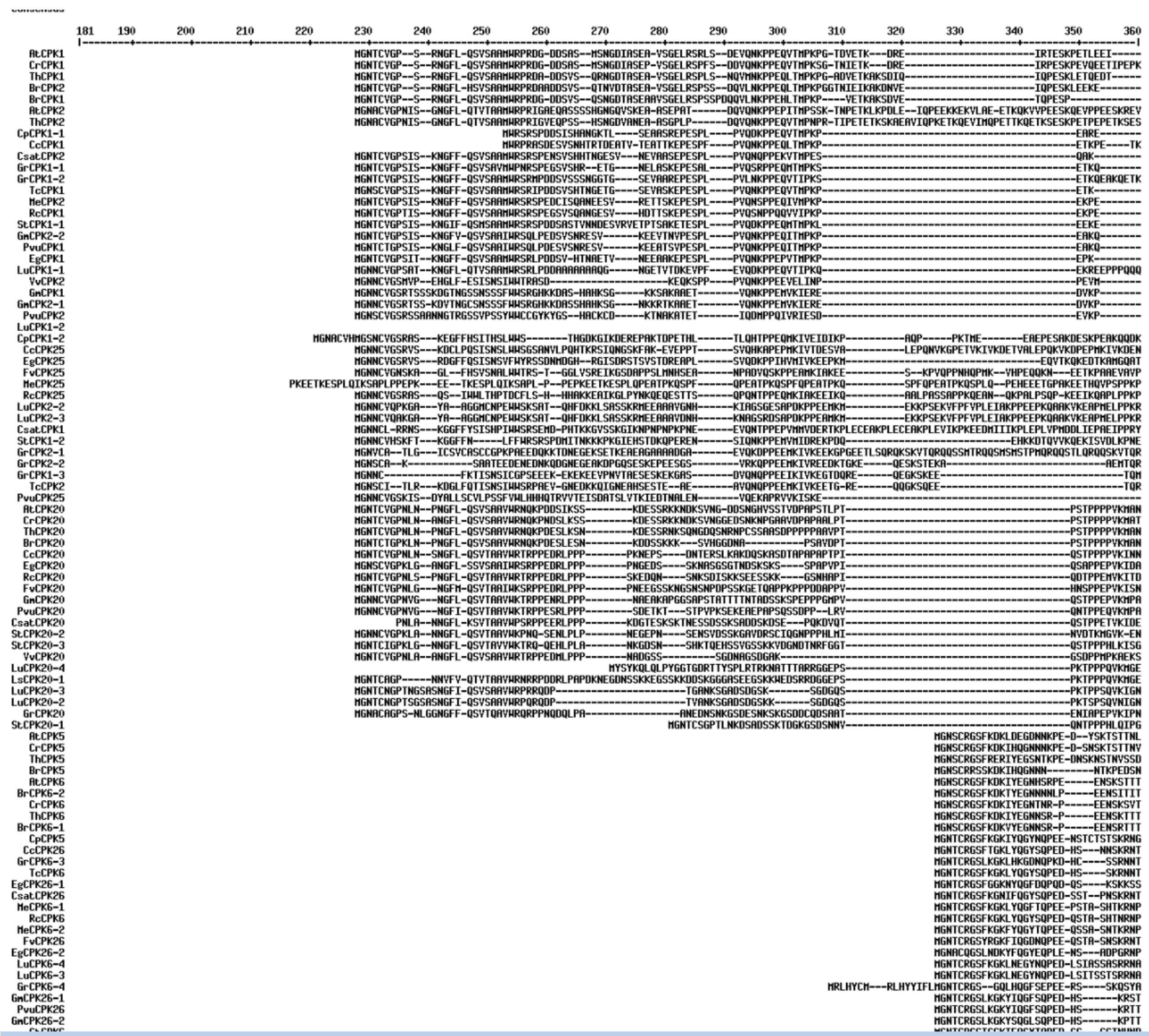

```
[illegible]
BrCPK11-2
AtCPK3       MGHRHS----KSKSSDPPPSSSSSSSG-NVVH
CrCPK32-2    MGHRHS----KSKSSDPP-SSSSSSSD-NVVH
BrCPK3-3     MGHRHS----KSKSSGPPPSSSSSSSSTNVVH
BrCPK3-4     MGHRHS----KSKSSGPPPSSSSSSSSTNVVH
ThCPK3       MGHRHS----KSKSSGQPPSSG------NVIH
BrCPK3-1     MGHRHS----KSKTS------SSSSSSDNVVH
CpCPK3       MGNCNG---MPSNRTQIPSDTTTAASGP----
GrCPK3-3     MGNCNS---QPSADSQFRPETNTGGGPNHGIN
TcCPK3       MGNCNG---HPSADNQFRPDSDSGGGPHNGIN
EgCPK3       MGNCNG---LPSAYARHRPPGSADPHGGSGDG
MeCPK3       MGNCSSSSSLPSTFSANASDPSAASRPPPPSN
RcCPK3       MGNCSSN--LPSTSTFTADRPP----PPPPSH
CcCPK3       MGNCNG---LPSSQTQLHST------------
FvCPK3       MGGNCSS-----AAA-----DSPDPRPHLPSNG
GmCPK3-3     MGNCSS-----GAG-----APTTYSDDHPHHN
PvuCPK3-1    MGNCSS-----SHGGGGTPATSTYSDELPQRN
StCPK3       MGNCNS---------LSSDHPPPPAAAAGGGG
GmCPK3-1     MGN
GmCPK3-2     MRQKP--------------NVRLSHQSHMVSRFSPDRVNTKKQQRSNDTVSMGN
PvuCPK3-2    MGN
GrCPK3-1     MGNCNR----PPIVSNRFRAEPDPGGTHHNDD
LuCPK3
GrCPK3-2     MGNCNG---LPSTGNQFQPLSDSGVGLLDGGI
AtCPK17      M--------------GNCCSHGRD------SADNGDA--LENGASASNAA
CrCPK17      M--------------GNCCSHGRD------SADNGDGQTLENGATASNAA
ThCPK17      M--------------GNCCSHGRD------SA--GDGPNHQNVATASNTG
BrCPK17-1    M--------------GNCCSNGRD------SGDNGD----TRGITASNA-
BrCPK17-2    M--------------GNCCS-GRD------SE---DGHTQDKGLTDSNTA
AtCPK34      M--------------GNCCSHGRD------SDDNKEEPRPEN----GGGG
CrCPK34      M--------------GNCCSHGRD------SGTKEEQ---ALENGGGGGG
ThCPK34      M--------------GNCCSHGRD------SRNKKDDPTPANAAGGGGGA
BrCPK34-1    M--------------GNCC--GRD------TGNNNGEPTYEN--------
BrCPK34-2    M--------------GNCCAHGRE------EPTQEK------------GL
CpCPK17      M--------------GNCCSQGNSAD--APN-SEKGETTPENGTDNPHSA
RcCPK17      M--------------GNCCSRGDSGD--AAE-NEKGEAVANNTNGSVANN
TcCPK34      M--------------GNCCSRGNPAG--APNTNEKGDATPDNNGNNPSSS
MeCPK17-1    M--------------GNCCSRG-ESG--DAPENDKGDQGNDNGSNNGGSD
MeCPK17-2    M--------------ANVVALGGDSG--DAPENEKGDQAE--GSNNPDDA
CsatCPK17    M--------------GNCCSRENPEE--APATSVK-EGSNDADNSKDDGG
EgCPK34      M--------------GNCCFRGGPAN--PLSTKAL-SMAASRNNHSPDGA
LuCPK17-1    M--------------GNCCSRGNEGD--EASNDEKGEDAQQQGGDSSNPG
LuCPK17-2    M--------------GNCCSRGNEGD--EASNDGKGEDAQQQGGGSSNPG
LuCPK34      M--------------GNCCSRGKEGDGREKGGDQGSNNNGVGGGDTTSSG
CcCPK17      M--------------GNCCSNGKDESEKEEKATAGNGGVNDGAGGNGTMD
GrCPK17-1    M--------------GNLCSRSDP----AANPDEKGEPGPENELNTST--
GrCPK17-2    M--------------GNCCSRGNP----ESLTNELGVASPENNGNVSICS
StCPK17-1    M--------------GGCCSKAET----DPAQNNEEIAQSYSKQGESHAG
StCPK17-2    M--------------GNCCSRGQ------PNDDDHIMSKEKSNDDQSKSC
GrCPK17-3    M--------------GNCCTRGDG-----SDKLEKAAAGYGNGDADPTVT
GrCPK17-4    M--------------GNCCSRGSP-----AAE----DANDDKGDAPKQAE
GrCPk17-5    M--------------GNCCSCGSS-----AEDANEKGDTITAEEAKSPNG
GmCPK17-1    M--------------GNCCSGGT---------NDTEDKGESNQPTNNNNG
GmCPK17-2    M--------------GNCCSGGT---------NDTEDKRETNQPTSDNGG
PvuCPK17-1   M--------------GNCCSGGT---------NDCDNKGETNQNNNNDEG
GmCPK17-3    M--------------GNCCSQGNVADGPADDSAAANDKGEANQQGSNNHA
GmCPK17-4    M--------------GNCCSQGNGADGPSDD-AAANDKGETNQQ-SSNHA
PvuCPK17-2   M--------------GNCCSQGNAADGPADN-TAADDKGETNQQ-TNNHS
FvCPK34      M--------------GNCCSQRN-------TEDALNDEKGDNNGGLDMNA
VvCPK17
BrCPK34-3
AtCPK9       M--------------GNCFAKNHGL-MKPQQNGNTTRSVEVGV----TNQ
CrCPK9       M--------------GNCFAKNNGL-MKPQQNGHTNRSVEVGV----TNQ
BrCPK9       M--------------GNCFAKNHGL-MKPQQNGQT-RSVEVDQ----THQ
ThCPK9       M--------------GNCFAKNHGL-VKPQQNGQT-RSVEIDHRGGGTHQ
AtCPK33      M--------------GNCLAKKYGLVMKPQQNGE--RSVEIENRRRSTHQ
CrCPK33      M--------------GNCLAKKYGVVMKPQQNGE------IENRGRSNHQ
CpCPK33      M--------------GCCFSDGKGSDRPENEYRPVAGTGQK--PYQF---
CcCPK9-3     M--------------GGCLTKSKDPKPRHYAHGYRSSPNTV--AKA----
GrCPK9-3     M--------------GGCLTKTKGSNPQHN-NGYKSGATTT--TAAEQ--
GrCPK9-2     M--------------GGCLTKNKDPMPQHN--GYRSGATTT--AAVHQQR
TcCPK33      M--------------GGCLTKSKEPKPKHN--GYRSGATTT--VAVHQQR
GrCPK9-1     M--------------GSCLTKSKDSKPKHN--GYGSGPTTT--AAVHQQR
LuCPK9-1     M--------------GHCISKGGRGGDAYRYNDNASANHHH--HHPKSRD
LuCPK9-2     M--------------GHCISKGGRGGDAYRYNDNASANHYH--HHPKSRD
MeCPK33-1    M--------------GLCLSKDKRTQ-PRFINDAAGIHNNK--RSQE---
MeCPK33-2    M--------------GYCLSKEKNTQRPRLYNGTAG---HK--KPQE---
RcCPK33      M--------------GFCLSKEKRSQ-QHHYNGATGL--QH--QSRE---
FvCPK33      M--------------GKCLSKSREPEPQHNGYRSGGGAG----AHYQKPH
GmCPK9-2     M--------------GCCASK--ESEPQHNGYRHAGTGG----VHNQKTH
GmCPK9-1     M--------------GCCVSK--DSEPQHNGYRHGGTGG----VHNQKTH
GmCPK33      M--------------GCCLSKKGSEPEEHIGYRHVAGAGGAGVYNNHKSH
GmCPK9-3     M--------------GCCMSKKGSEPEEHIVYRHVAGAGGAGVYNNHKSH
PvuCPK9-2    M--------------GGCLSKNGSESEEHNGYRHVVGGA-----HNHKSH
EgCPK33-2    M--------------GCCFTKDKAPEPQQNGYRSAGENYQRPHEHHHQRH
EgCPK33-1    M--------------GCCFTKDKAPEPQQNGYRSAGENYQRPHEHHHQRH
CpCPK21      M--------------GCCSSKPKAQQAENMSGYRSEL--PNSCQSQQHQH
RcCPK21      M--------------GCCGSKEKPSKPKNDNGYRPAN--TGNSRPQQQQQ
CcCPK15      M--------------GCWGSKERVP-PSDMNGYRSST--AHSSHQATGVA
[illegible]
```

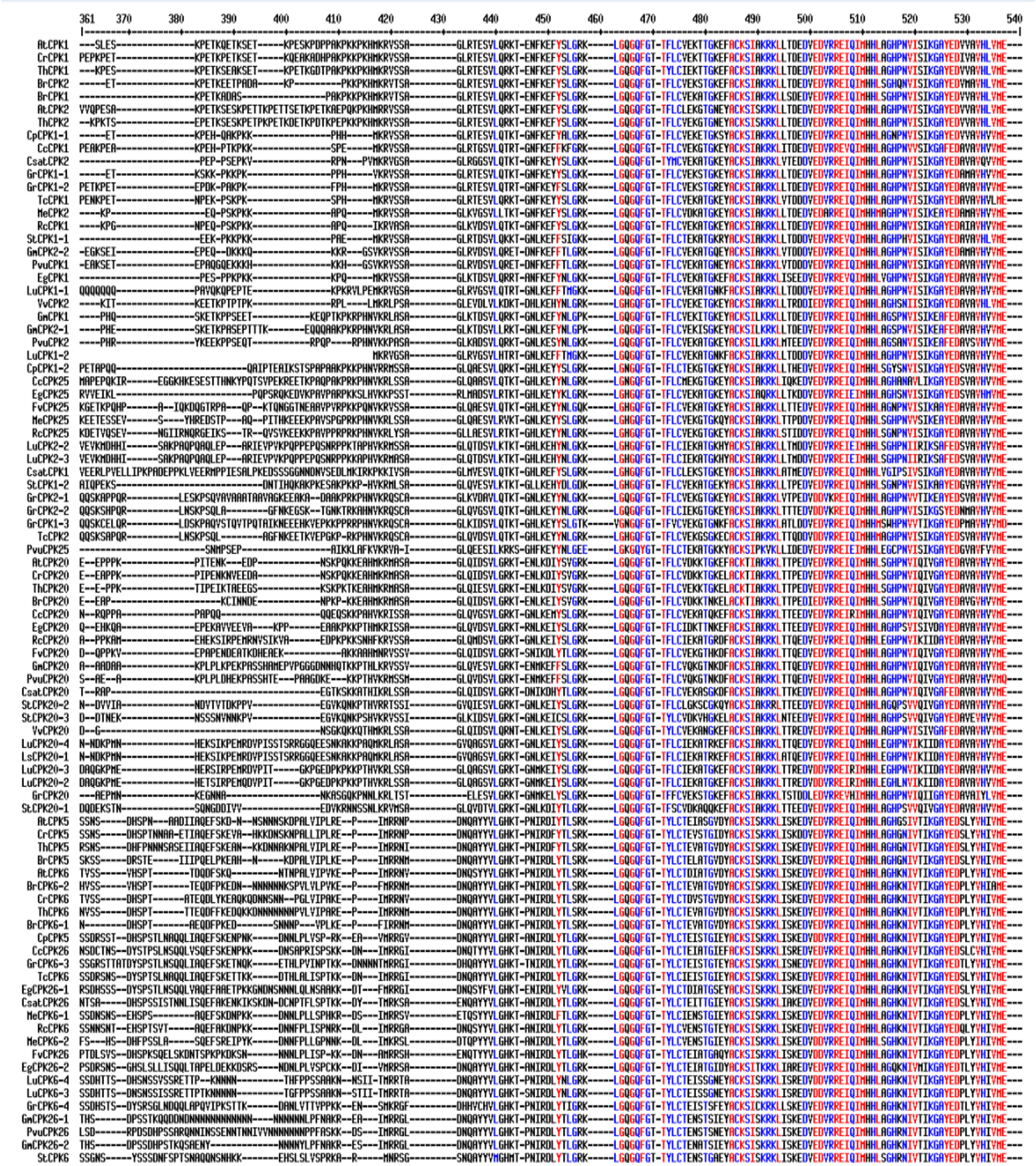

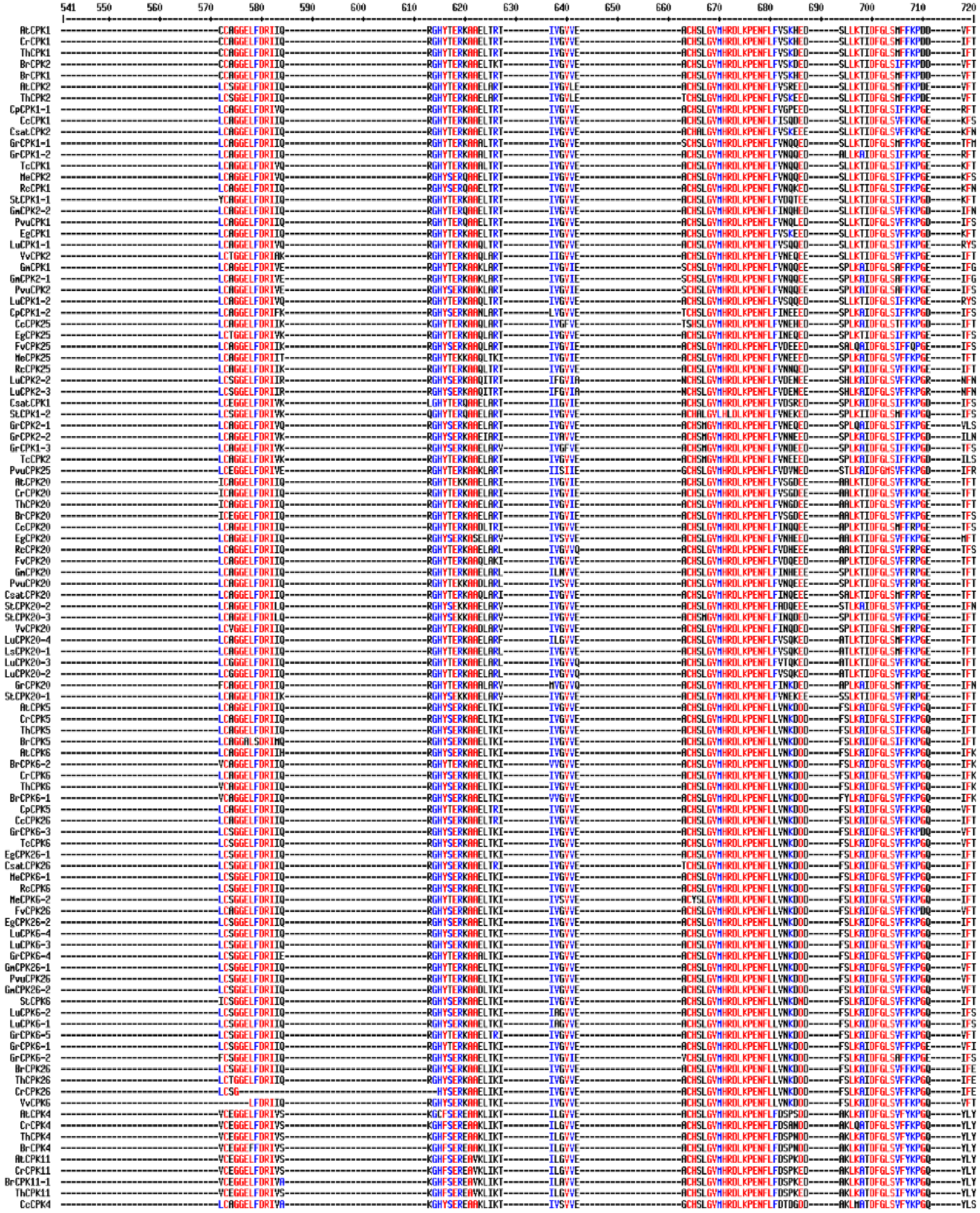
541 550 560 570 580 590 600 610 620 630 640 650 660 670 680 690 700 710 720

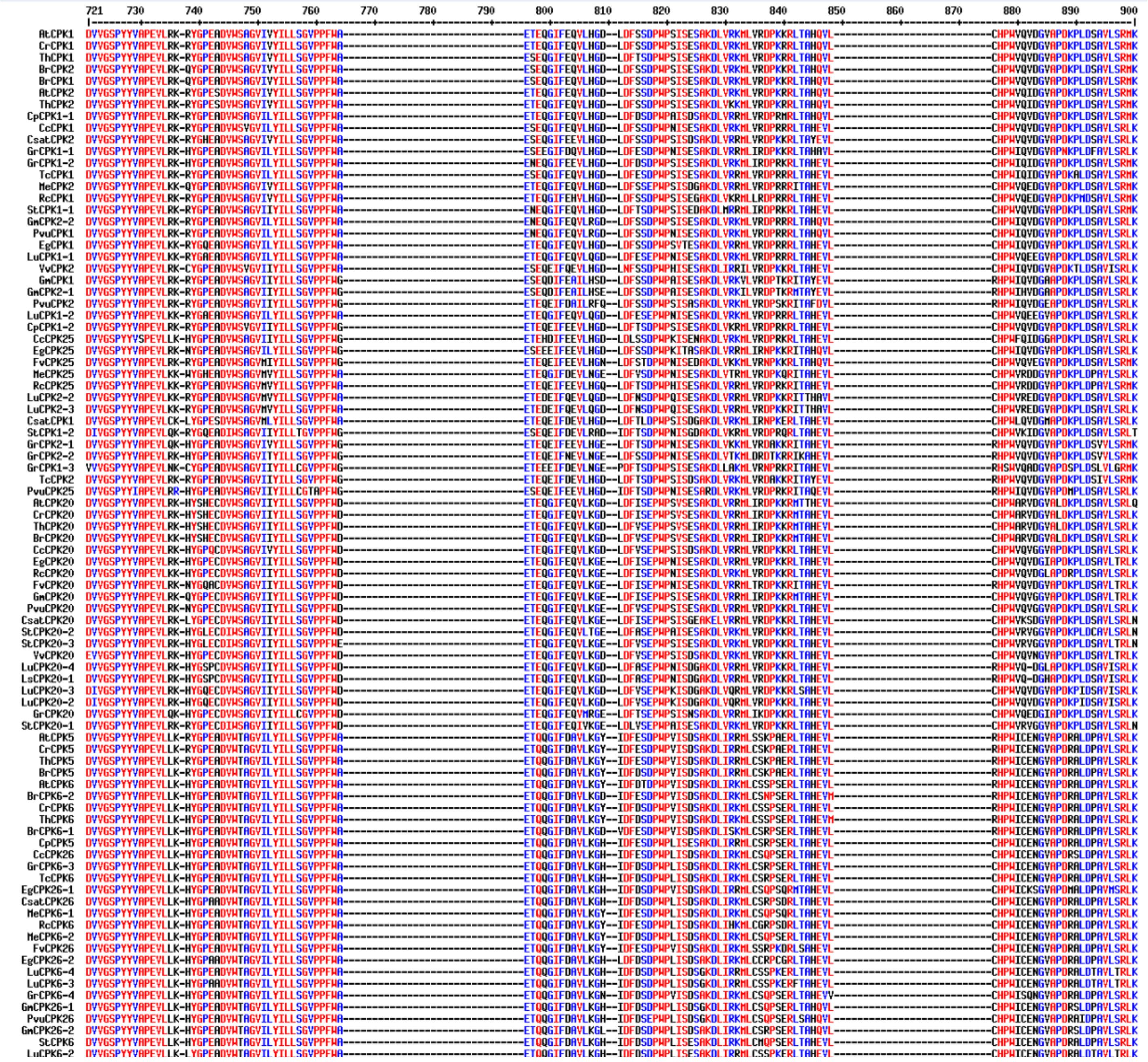

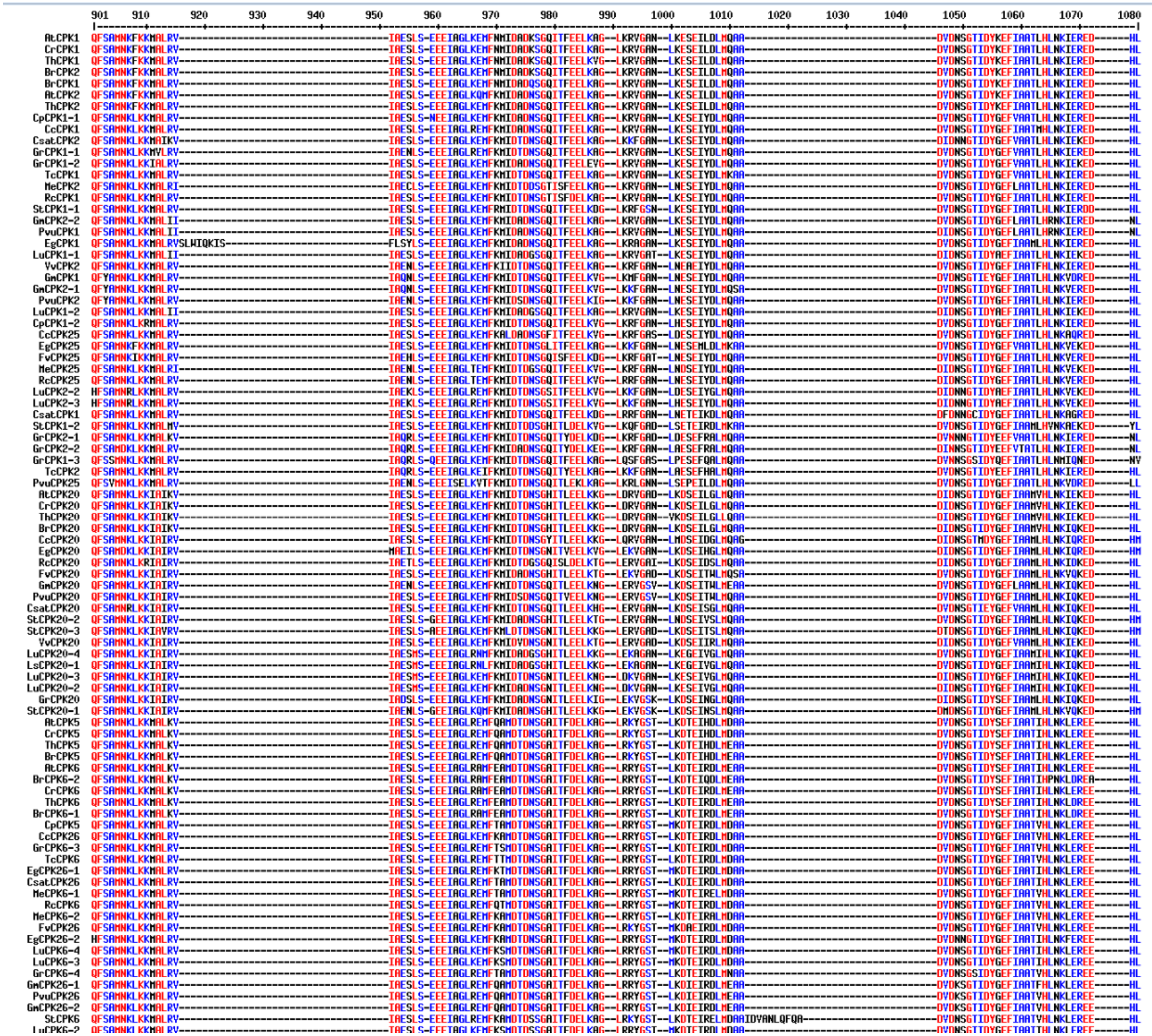

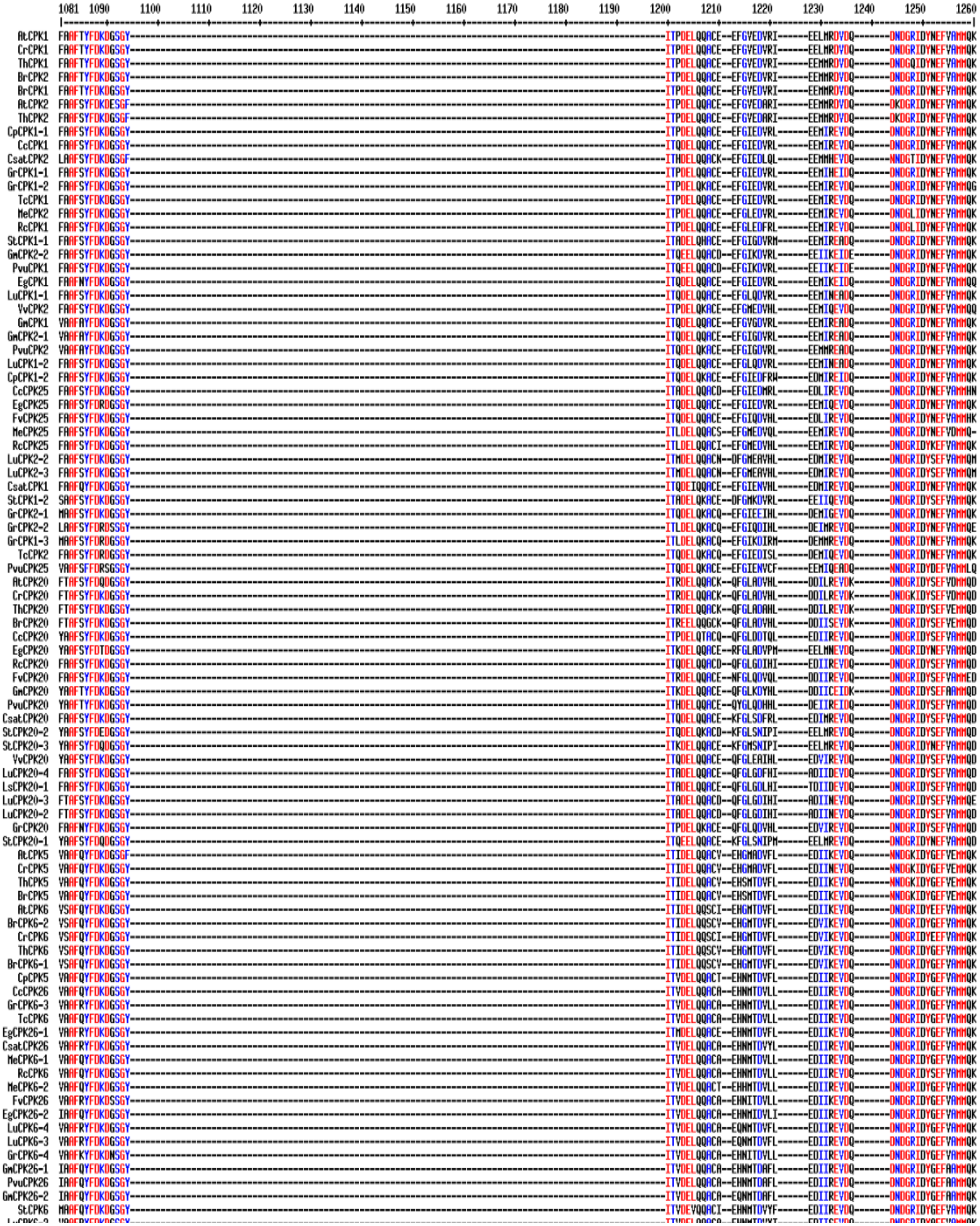

Supplement: Supplementary file 10 [file Image3.PDF]
