# Supplementary material for: Genome-Wide Identification of Calcium Dependent Protein Kinase Gene Family in Plant Lineage Shows Presence of Novel D-x-D and D-E-L Motifs in EF-Hand Domain
Source: Front Plant Sci. 2015 Dec 24;6:1146. doi: 10.3389/fpls.2015.01146 (PMC4690006; doi:10.3389/fpls.2015.01146)
Supplement: Supplementary file 11 [file Image4.PDF]

Supplementary Figure 4

Multiple sequence alignment of CPKs of lower eukaryotic plant. Amino acids in red color indicate the 90% consensus level and are conserved throughout the lower eukaryotic plant lineage.

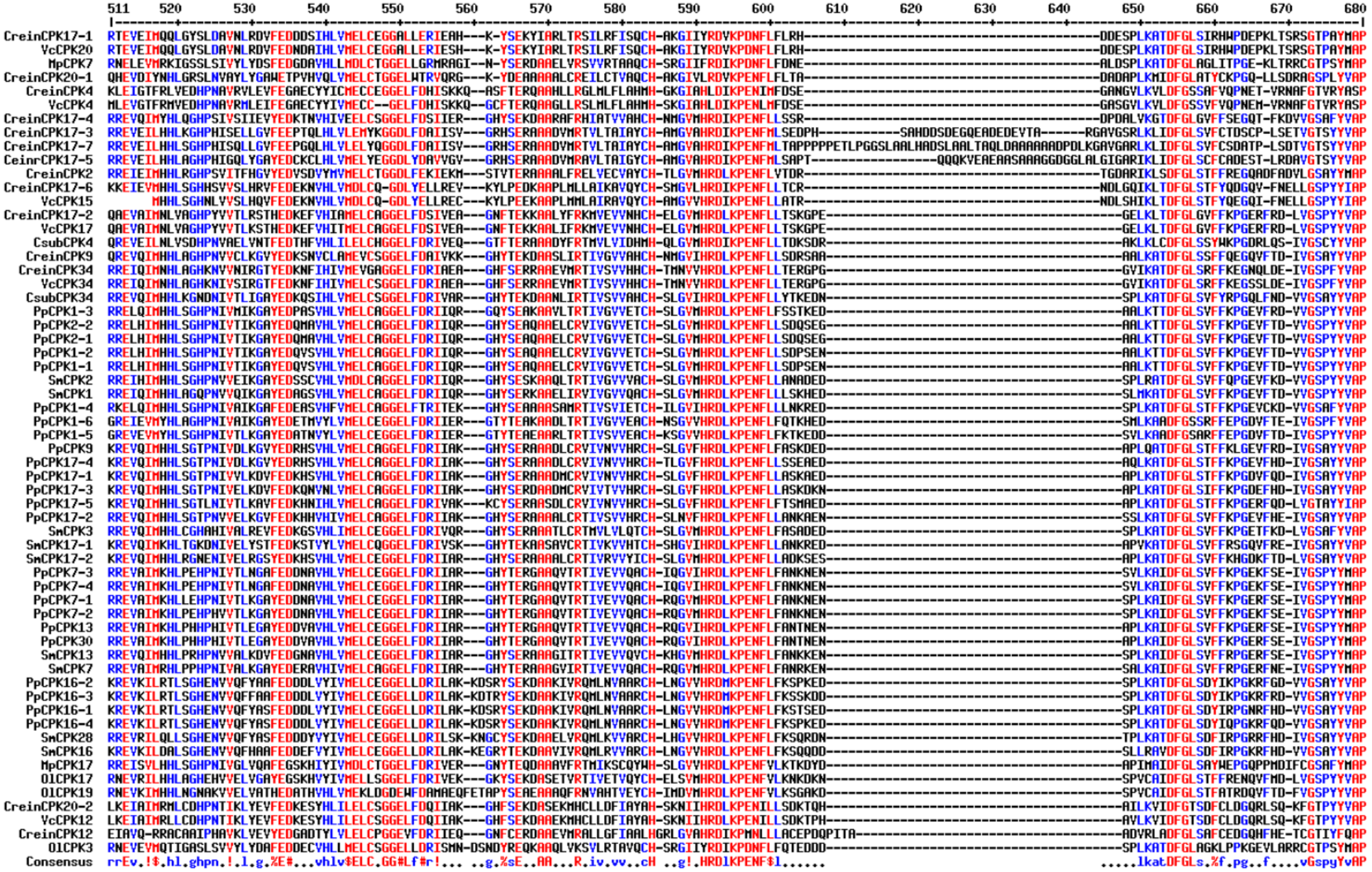

```
              681       690       700       710       720       730       740       750       760       770       780       790       800       810       820       830       840       850
              |--------+---------+---------+---------+---------+---------+---------+---------+---------+---------+---------+---------+---------+---------+---------+---------+---------|
  CreinCPK17-1 ELV-----LQSYDEKCDIWSVGMLTYQLLTGRFPFWEDV------------RTQ-TLSDVWKAILT----QD-VNWDAQELKQLSPSAVDLLKRLLQRDPAQRPSAFAALEHPWL----------------SEEGRA-RDLPLQGSVVQRLQRFSTFGHLK---------
       VcCPK20 ELV-----LQSYDEKCDIWSVGMLTYQLLTGRFPFWEDV------------RTQ-TLSDVWKAILT----QD-VNWNALELKQLSPSAVDLLKRLLQRDPAQRPSAFVALEHPWL----------------SEDGRA-RDLPLQGSVVQRLQRFSTFGHLK---------
        MpCPK7 EVI-----NRNYGEEADVWSCGVVAYQLLTGRLPFVDKV------------NQRPNAKEVFRAILE----DQ-IDFVADPWPRLSPECVDLVRKMLDRDPETRITARAALLHPWLL------------APTSEEALSGATQPIGGQVVARLQRFSTYGLLK---------
  CreinCPK20-1 EVL-----KQSYGQPCDVWSAGVVAYQLLTGRFPFEDEDCGLLLSSLDVLGKKHFTNKEIFFAILY----GD-LDFRRPPWDEISELARDFVAHLLERDPAKRPSAEQALKHPWLRQFDAS-----ASGTSATSSTASVDGGSNGGGAATTDGNGSAASRS---------
     CreinCPK4 EMA-----NDVCGQKADIWSVGVVMYILLSGRAPFLKS-----------------NDVDTLNLIKS----GPRVKFAGERWAGISQAAKDCIKALLEPNPRLRPSAVTVLNMPWLKQQ----------APETIIVPDTLRHLRAFANQSRIRRLLLGLMAD---------
        VcCPK4 EMA-----NDVCGQKADIWSAGVVMYILLCGKAPFLKN-----------------NDIDTLDYIKN----GPRVKFNGERWSSISQCAKDCIRTLLEPNPRARPTAVEVLGMPWLKQQ----------VPETVIVPDTLKHLRTFANQSRIRRLLLGLMAD---------
  CreinCPK17-4 EVL-----RKKYDKRADIWSLGVLLYLLLAGVPPFYAE-----------------TEREIFRAVLS----AP-LDFTSAPWPSVSEAAKDVIRRMLERDPSKRISIADVLAHEWVREDGA--------ARAAPLQHEVLVRLQNFAALNKLQQEALKIIAN---------
  CreinCPK17-3 EVL-----AGCYSRAADVWSAGVILHIMLAGYAPFDGR-----------------DDDHILRAILK----GNLDLKTDPIWQSISREAVSALTAMLEKDPAKRATADQLLAMPWFGRTAAA-----CAAPSAPLPGVVSERMRRFARMNSFKREARRVVAG---------
  CreinCPK17-7 EVL-----AKSYSRSADVWSAGVILHILLTGYAPFDGR-----------------NDQEILKAVQQ----GHLDLASDPIWSSISREAMAALTAMLDRDPQRRATADQLLAMPWFGRTAAA-----CRAPCVPLPGVVSERMRRFARMNSFQKEARRVVAG---------
  CeinrCPK17-5 EVLE----ANGYGRAADVWSAGIILHVLLLGYAPFNGA-----------------DDKEIQLSVLK----GVPDSTGDPLWTSLSPAAVDTLAAMLDRNPATRATADQVLSSPWLGRTAAE-----CTAPSTPLPGVVSERLRKFAKLNSLQKEARRLVAT---------
     CreinCPK2 EVL-----ERSYGKSCDVWSLGVILHVLLSGAPPFAGA-----------------TDAEIIKAVRS----RD-VDLSAKPWPAISRHARHLVSRMLEKDPEQRIQLDQVLTHPWLRLDGT--------APVRAVPTGVRDRVRQFRTLNLLKREARRVMAA---------
  CreinCPK17-6 EVI-----KQKYGKEADIWSCGVMLYIMLCGEAPFFGE-----------------GDEGIFKSIMK----AK-LDFSFEPWPSLSRDVKLLLRRMLVKDPRERASLEEVISHPWFARYSPS-----LGTTSMQLKLEALANGGS-GSQGVPGRTGAPAGLP---------
       VcCPK15 EVI-----RQKYGKEADVWSCGVMLYIILCGEAPFFGE-----------------GDEGIFKSIMK----AK-LDFSFEPWPSLSRDVKMLLRRMLVKDPRERATLEEVLAHPWFARYTP--------------QPDSIKAPGP-PAPGAERTGGPASMLP---------
  CreinCPK17-2 EVL-----RKNYSHEADMWSLGVILYILLSGLPPFWGD-----------------TEDQIFKMVLK----GHI-DFKTDPWPKISEAAKDCVKRLLDQDVTKRATAAQILKHEWLVKEGVA--------LDIALDSVVLKRLKQFAQMNKLKKACLMVIGQ---------
       VcCPK17 EVL-----RKNYSHEADMWSLGVILYILLSGLPPFWGD-----------------TEDQIFKMVLK----GHL-DFKTEPWPKISDAAKDCVRRLLDQDVTKRATAAQILKHEWLIKEGVA--------LDIALDSVVLKRLKQFAQMNKLKKACLMVIGQ---------
      CsubCPK4 EVL-----RRDYGVEADMWSLGVMLYILLSGLPPFWGD-----------------KEEDIFRMVLK----ADI-DFQTPPWPSISQAAKDCVRQLLNVAPDGRPIASKLLQHAWLKAQGAS--------LDKPLDSVVLSRIKKLSAMNKLKKAALVVMAQ---------
     CreinCPK9 EVL-----KRAYSKEADIWSCGVILYILLCGYPPFHGE-----------------NEKKIFEAVVT----KPV-DFTSDPWPAISEAAKDCVRRMLVRDPKRRATATQILQHEWMRENGCA--------ADQPIQLEVLSRMKQFSAMNRLKKEALKLIAK---------
    CreinCPK34 EVL-----KRSYGKEADIWSCGVILYILLCGWPPFHGD-----------------STQAIFKNILS----APL-DLKTEPWSRVSADAKDCVRRMLARDPRKRLTAEQVLNHPWMRENGAA--------PDEAFVPEILIRMRQFTKMNMLKREALKVIAR---------
       VcCPK34 EVL-----KRAYGKEADIWSCGVILYILLCGWPPFHGD-----------------STQAIFKNILS----APL-DLKSEPWPRVSPDAKDCVRRMLARDPRKRLTAEQVLNHHWMRENGAA--------PDEAFVPEILIRMRQFTKMNLLKREALKVIAR---------
     CsubCPK34 EVL-----KRKYSKEADIWSCGVMLYILLSGVPPFWGE-----------------TEQQIFDAVAK----GYI-DFATDPWPHISSEAKSAVRAMLQQDPKKRATAEQILGHSWMKENGTA--------SDKPLDNVILQRMRGFLNMNKLKKEALKVIAG---------
      PpCPK1-3 EVL-----RKNYGPEADVWSAGVILYILLSGVPPFWAE-----------------TEQGIFEQVLK----SEL-DFASDPWPKISDSAKDLIRKMLNPQASKRLKAHQVLCHPWICEDGVA--------PDRPIDSAVQSRLKHFSAMNKLKKIAIRVIAE---------
      PpCPK2-2 EVL-----RKHYGPEADVWSAGVILYILLSGVPPFWAE-----------------TEQGIFEQVLK----GEL-DFVSEPWPSISDSAKDLIRRMLDPNAKRRLKAHQVLSHPWIGEEGVA--------PDRPMDPAVQSRLKQFSAMNKLKKVAIRVIAE---------
      PpCPK2-1 EVL-----RKHYGPEADVWSAGVILYILLSGVPPFWAE-----------------TEQGIFEQVLK----GEL-DFVSEPWPSISDSAKDLIRRMLDPNAKRRLKAHQVLSHPWIGEEGVA--------PDRPMDPAVQSRLKQFSAMNKLKKVAIRVIAE---------
      PpCPK1-2 EVL-----RKHYGPEADVWSAGVILYILLSGVPPFWAE-----------------TEQGIFEQVLA----GEL-DFVSEPWPSISESAKDLIRRMLDPVAKRRLKAHQVLSHPWIREAGVA--------PDRPMDPAVQSRLKQFSAMNKLKKVAIRVIAE---------
      PpCPK1-1 EVL-----RKHYGPEADVWSAGVILYILLSGVPPFWAE-----------------TEQGIFEQVLA----GEL-DFVSEPWPSISESAKDLIRRMLDPVAKRRLKAHQVLSHPWIREAGVA--------PDRPMDPAVQSRLKQFSAMNKLKKVAIRVIAE---------
        SmCPK2 EVL-----RKQYGPEADVWSAGVILYILLSGVPPFWAE-----------------TEQGIFEQVLQ----GDI-DFESDPWPKISESAKDLIRKMLTRNPRKRLKAQEVLTHPWIMEGGVA--------PDAPIDSAVLSRLKHFSAMNKIKKIALRVIAE---------
        SmCPK1 EVL-----RKKYGPEADVWSAGVILYILLSGVPPFWAE-----------------TEKGIFEQVLK----GEI-DFESHPWPVISQDAKDLIRKMLCPVPANRLKAHEVLGHPWARADGVA--------PDKPLDSAVLSRMKQFSAMNKIKKIALRVIAE---------
      PpCPK1-4 EVL-----RRKYGPESDIWSAGVILYILLSGVPPFWAD-----------------TEDGIFAEVLK----AKV-DFDTDPWPKISKDAKDLIRKILNPDVKARLTASEVLAHPWVREKGVA--------STKPMDSSVQNRLKRFAAMNKMKKLAVRVIAQ---------
      PpCPK1-6 EVL-----DRHYGPEADIWSAGVILYILLSGVPPFWAE-----------------TVQGIFEEVMK----GEPPSFAADPWPNISEGAKDLIRQMLNPDPRKRLTAAEVLKHPWIREDGVA--------SNKPIASLVQFRLKQFSAMNKLKKLAIRIIAE---------
      PpCPK1-5 EVL-----DRHYGPEADIWSAGVMLYILLSGAPPFWAE-----------------TVQGIFEKVME----GEPPTFTADPWPNISEVAKDLIRKMLDPNPEKRLKAHEVLNHPWIREDGVA--------PKKPIASLVQFRMKQFAAMNKLKKLAIRIIAE---------
        PpCPK9 EVL-----KRNYGPEADVWSAGVIVYILLCGVPPFWAE-----------------SEQGIFDAVLK----GHI-DFESEPWPRISSGAVDLVRNMLNPNVKERLTAYQVLNHPWMQEGGDA--------SDEPLDNAVLDRLKNFSAANKMKKLALKVIAN---------
     PpCPK17-4 EVL-----RRNYGPEADVWSAGVIVYILLCGVPPFWAE-----------------TEQGIFDAVLR----GHI-DFVSDPWPKISSGAKDLVRKMLNMNVKERLTAYQVLNHPWMEEGGDA--------SDTPLDNAVLTRLKNFSTANKMKKLALKVIAK---------
     PpCPK17-1 EVL-----KRSYGPEADVWSAGVIVYILLCGVPPFWAE-----------------TEQGIFDAVLK----GHI-DFENDPWPKISNGAKDLVRKMLNPNVKIRLTAQQVLNHPWMKEDGDA--------PDVPLDNAVLTRLKNFSAANKMKKLALKVIAE---------
     PpCPK17-3 EVL-----KRSYGPEADVWSAGVIVYILLCGVPPFWAE-----------------TEKEIFDTIMR----GHI-DFKSDPWPKISDEAKDLVKKMLNSNVKERLTAQEVLNHPWMQRDG-V--------PDVPLDNAVLTRLRNFSAANKMKKLALKVIAD---------
     PpCPK17-5 EVL-----RKDYGPEADVWSAGVILYILLCGVPPFWAE-----------------TEKGIFDAIMR----GTL-DFTSDPWPRISDDAKVLVKGMLNPDVNARLTAQQVLDHPWMKEDG-A--------SNAPLDNAVLTRLKNFSAANKMKKLALKVIAQ---------
     PpCPK17-2 EVL-----RRNYGPEADVWSAGVIVYILLCGVPPFWAE-----------------SEQGIFDAVLK----GYI-DFKSDPWPKVSAAAKDLVSKMLKQDPKERLTAQEVLKHPWMKEDGDA--------PDEPLDNAVLTRLKNFSSANKMKKLALQVIAQ---------
        SmCPK3 EVL-----RRNYGPEADIWSAGVILYILLSGVPPFWAE-----------------TEQGIFDEVLR----GHL-DLSSDPWPSISDGAKDLLRKMLKQDPKERMTPLEVLNHPWLKEDGEA--------PDVPLDNAVLSRMKQFSAMNKLKKLALKVIAE---------
     SmCPK17-1 EVL-----KKSYGPEADVWSAGVILYVLLAGVPPFWAE-----------------TEQGIFEAVLR----GHL-DLNGSPWPTISASAKDLVRKMLKQNPRERLSAADVLQHPWIKEDGDA--------PDKLIDGEVLSRMKNFSAMNKLKKVALKIISE---------
     SmCPK17-2 EVL-----RRNYGPEADVWSAGVILYILLSGVPPFWAE-----------------SEQGIFDAVLR----GQV-DFVSDPWPSISSSAKDLVKKMLTQDVKKRVTAAEVLSHPWVREDGDA--------PDAPLDNAVLSRMKQFTAMNKLKKLALKVIAA---------
      PpCPK7-3 EVL-----KRNYGPEVDVWSAGVILYILLCGVPPFWAE-----------------TEQGVAQAILR----GVL-DFKRDPWPKVSDSAKSLVRRMLEPDPKARYNAQQVLDHPWLQNAKKN--------PNVPL-DTVRSRLKQFSAMNKLKKRALQVIAE---------
      PpCPK7-4 EVL-----KRNYGPEVDVWSAGVILYILLCGVPPFWAE-----------------TEQGVAQAILR----GVL-DFKRDPWPKVSDSAKSLVRRMLEPDPKARYNAQQVLDHPWLQNAKKN--------PNVPL-DTVRSRLKQFSAMNKLKKRALQVIAE---------
      PpCPK7-1 EVL-----KRNYGPEVDVWSAGVILYILLCGVPPFWAE-----------------TEQGVAQAILR----GIL-DFKRDPWPKVSDSAKSLVRHMLEPDPKARYSAQQVLDHPWLQNAKKN--------PNVPL-DAVRSRLKQFSAMNKLKKRALQVIAE---------
      PpCPK7-2 EVL-----KRNYGPEVDVWSAGVILYILLCGVPPFWAE-----------------TEQGVAQAILR----GLL-DFKRDPWPKVSDSAKSLVRHMLEPDPKARYNAQQVLDHPWLQNAKKN--------PNVPL-DAVRSRLKQFSAMNKLKKRALQVIAE---------
       PpCPK13 EVL-----KRNYGPEVDVWSAGVILYILLCGVPPFWAE-----------------TEQGVAQAILR----GIL-DFKREPWPKVSETAKSLVRHMLEPDPKARYNAQQVLDHPWLQNAKKN--------PNVPL-DAVRSRLKQFSAMNKLKKRALQVIAE---------
       PpCPK30 EVL-----KRNYGPEVDVWSAGVILYILLCGVPPFWAE-----------------TEQGVAQAILR----GIL-DFKREPWPKVSETAKSLVRHMLEPDPKARYNAQQVLDHPWLQNAKKN--------PNVPL-DAVRSRLKQFSAMNKLKKRALQVIAE---------
       SmCPK13 EVL-----KRNYGPEIDVWSAGVILYILLSGVPPFWAE-----------------SEQGVAQAILR----GQI-EFKRDPWPKISENAKSLVQNMLMPDPKQRYTAQQVLDHPWLQNAKKA--------SNVPLGDVVRNRLKQFSAMNKLKRKALQMITE---------
        SmCPK7 EVL-----KRNYGPEVDVWSAGVILYILLCGVPPFWAE-----------------SEQGVAQAILR----GVL-DFRREPWPAVSESAKSLVRRMLEPDPRLRLSASQVLEHPWLHRAGKH-------APNVPL-DAVRTRLKQFAALNKLRKKALEIIAE---------
     PpCPK16-2 EVL-----NRKSGPESDVWSIGVITYILLCGRRPFWDK-----------------TEAGIFNEVLK----KKP-DFREKPWPSITASAQDFVKKLLVKDPHMRLTAAQALSHPWVKEGGDA--------SDMPLDISVLSNMREFVKYSRLKQLALRALAS---------
     PpCPK16-3 EVL-----NRKSGPESDVWSIGVITYILLCGRRPFWDK-----------------TEAGIFNEVLK----KKP-DFREKPWPSITASAQDFVKKLLRKDPHMRLTAAQALSHPWVKEGGDA--------SDMPLDISVLSNMREFVKYSRLKQLALRALAS---------
     PpCPK16-1 EVL-----KKKSGPESDVWSIGVITYILLCGRRPFWDK-----------------TEKGIFDEVLK----KNP-DYGEKPWPTISSSAKDFVKKLLVKDPAARLTAAQALSHPWAKEGGDA--------LDIPLDISVLSNMREFVKYSRLKQLALRALAS---------
     PpCPK16-4 EVL-----KRKSGPESDVWSIGVITYILLCGRRPFWDK-----------------TEQGIFNEVLK----KKP-DFREKPWPTISLSAKDFVKKLLVKDAAARLTAAQALSHPWAKEGGDA--------LDIPLDISVLSNMREFVKYSRLKQLALRALAS---------
       SmCPK28 EVL-----KRKSGPESDVWSIGVITYILLCGKRPFWDK-----------------TEAGIFNEVLK----KKP-EFRIKPWPSISANAKDFVKRLLVKDPRARPTAAQALSHPWVREGGCA--------PDFPLDIIVLSNMREFVKFSRLKQFAFRALAT---------
       SmCPK16 EVL-----KRKSGPESDVWSVGVITYILLCGRRPFWDK-----------------TEAGIFNEVLK----RKP-DLRERPWPSIGSSAKDFVRKLLVKDPRARLTAAQALSHPWVREGGDA--------LEIPLDISVLSNMREFVKYSRLKQLAFRALAT---------
       MpCPK17 EIF-----NKKYDAGVDIWACGVILYILLSGGPPFIAD-----------------TDKGIQDMIL-----ACKYDLAASRWPKISASAKDLIRKMLMKKPKDRLTAEEVLNHPWVREDGDA--------PATKIEDEVLSRMKNFSAMNKFKKLGLMAMAR---------
       OlCPK17 EVL-----RRRYSNESDVWSCGIILYILLSGVPPFWAQ-----------------TEQGIFDAILQ---GA--LDLESDPWPKISAEAKELISGMLQADPKKRLTAAQVLKHPWVADPNVA--------PKTKLDNVVFKRFKNFAGISKFKRMGLMAMAR---------
       OlCPK19 EVM-----AYKYSNGADVWSCGVILYILLSGVPPFWGS-----------------SEKQVFDAIKRYKTGAEPLDLVSEPWPSVSDAAKELIQGMLTVDPTKRMTIEDVLQHPWLADPSVA--------PKTALDNIVYKRFKNFAAMSKFKKLGLHAMAR---------
  CreinCPK20-2 EVL-----KKDYDKSADIWSAGVIMFILLCGYPPFGGK-----------------TDAKILQRVQ-----AGQYSFDGKEWEVVTEAAKDMIAHMLVMDIAKRATAKQLLQHRWFQVAATA--------PAAALGAHMVKRLRAFAGMSRMKRLALVVLAR---------
       VcCPK12 EVL-----KKDYDKSADIWSAGVIMFILLCGYPPFGGK-----------------TDAKILQRVQ-----AGQFSFDGKEWEVVTEAAKDMISQMLVMDITKRATAKQLLQHRWFQVAATA--------PAAALGAHMVKRLRAFAGMSRMKRLALVVLAR---------
    CreinCPK12 EVVGDASGRVRYDKGADVWAAGVTMYVLLCGWVPFGGR-----------------YAFKRSEVELRKIIRAGKFCFTGLVWEVVSDSAKDLIRQLLTPDPAQRPSAAQLLSHPWFHEAASL---------QRPLGQRMITNLKQFAAACRLQKLAALVVAKRGGG-----
        OlCPK3 EVI-----ERNYGSQADVWSAGVVAYQLLSGRLPFIDK-----------------VNARPNAKEVFRAILEDEVDLESDPWSQVSDEAKDFVRKLLDRDPEARPIARAALLHPWLTQTSASWKAKAAKGQDSALDGQVVARLQRFATQGLLKRSVLRLIARELLSEDEDD
     Consensus EVl     .r.ygpeaD!WSaGVilYiLL.G.pPFw.e                 te.g!f..!l.    g...df...pWp.iS..Akdlvr.$L..#p..R.ta.qvL.HpW..e.g.a        .p..pld..v..rlk.f.amnklkk.al.via.
```

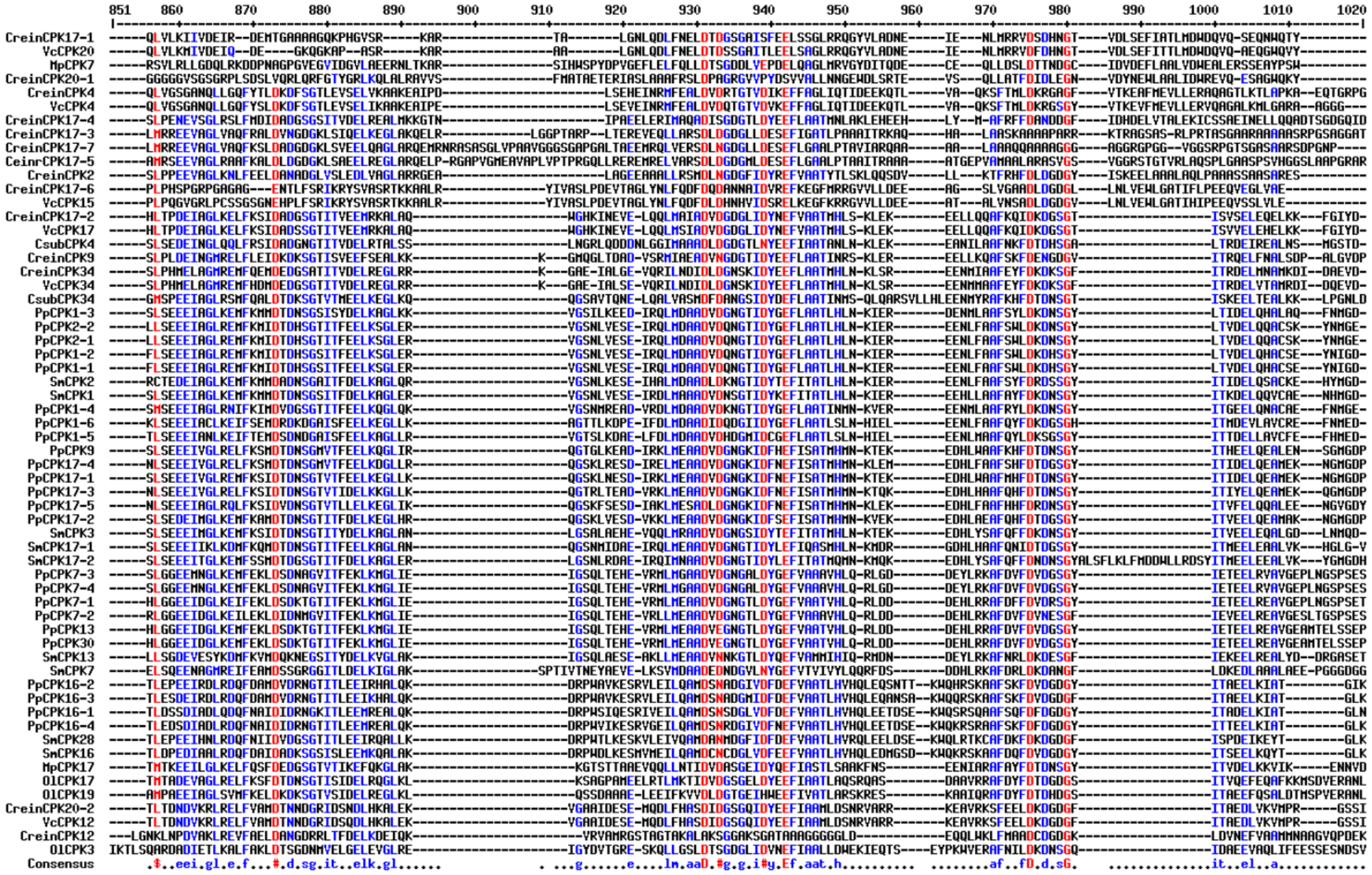

851 860 870 880 890 900 910 920 930 940 950 960 970 980 990 1000 1010 1020
CreinCPK17-1
VcCPK20
MpCPK7
CreinCPK20-1
CreinCPK4
VcCPK4
CreinCPK17-4
CreinCPK17-3
CreinCPK17-7
CeinrCPK17-5
CreinCPK2
CreinCPK17-6
VcCPK15
CreinCPK17-2
VcCPK17
CsubCPK4
CreinCPK9
CreinCPK34
VcCPK34
CsubCPK34
PpCPK1-3
PpCPK2-2
PpCPK2-1
PpCPK1-2
PpCPK1-1
SmCPK2
SmCPK1
PpCPK1-4
PpCPK1-6
PpCPK1-5
PpCPK9
PpCPK17-4
PpCPK17-1
PpCPK17-3
PpCPK17-5
PpCPK17-2
SmCPK3
SmCPK17-1
SmCPK17-2
PpCPK7-3
PpCPK7-4
PpCPK7-1
PpCPK7-2
PpCPK13
PpCPK30
SmCPK13
SmCPK7
PpCPK16-2
PpCPK16-3
PpCPK16-1
PpCPK16-4
SmCPK28
SmCPK16
MpCPK17
OlCPK17
OlCPK19
CreinCPK20-2
VcCPK12
CreinCPK12
OlCPK3
Consensus

```
              1021  1030      1040      1050      1060      1070      1080      1090      1100      1110      1120      1130      1140      1150      1160      1170      1180      1190
              |--------+---------+---------+---------+---------+---------+---------+---------+---------+---------+---------+---------+---------+---------+---------+---------+---------|
 CreinCPK17-1 ----LDHAFRKMDHDGDGFISLDELLQQLPPLRPAPGAPAGGLAQVVGSAVSGAAAALGLGGRLEAYEGDAERLAEAKLMLREADTNGDGKISREEFFDLLRDGHAPDSLSFYDDRLAIGEDGRARPVTQAHPPSSQQQQQQGGLQTAQR
      VcCPK20 ----LDHAFRKMDHDGDGYISLDELLQQLPPLRPAPGV--GSLAQVV----SDAAAALGFS---RGYDGDAERLAEAKLMLREADTNGDGKISREEFFDLLKESHAPDSLSFYDDRLAITTDGRTSAVVGQQNRQPAAKL
       MpCPK7 ----VKEAFNMLDKDGNGTIDAGEVAE-LVFMNEDDGK----------------------------LTSEAKKVIAA--CISEADTDGDGLIDLDEFITLLQMDPT-DRLDAYELRLSKRGGSVDLSADETVLVD
 CreinCPK20-1 ----VNQVFDMFDQGHTGRLTPENIQRVLCGGGLLGAAGSGSSGS--------EADVLADPGGADAELDECPFDDVVPAAMREADEDHDGSISREEFVRFLSTAPG-DKLEFYESRRKRLGGSSSGSDGNGGGGL
    CreinCPK4 PQSMDGSVASSHAPVTHGRTSGADG--GEARVLLDPGL-VSELEQEFANLDANGDGVLSFEEFKAIL-----GIQSTPEGLQLTQPQLP----AAPSIAENMETLAQTLR-------TRTRSHSALDEARATSGSNTRTSLRGAHRHSNSGGPSSTGALGPGAGGAS-AT
       VcCPK4 ----DSPLRHEAELKLHGRQSGSEG--ADSKVILDPGL-VQELEAEFAYLDANGDGVLSFEEFKAIL-----GIQSTPEGLTLAQPQLA----AVPSLQEGMETLAQTLR-------TRTRSVSK-DEQYQQPPMQRLALARVSH---NGGGGSGTNG-GAGAGGNC-GP
 CreinCPK17-4 FEEFCHLMRCGNSALTKATTTVKQGLMGTVRSQAVLDL-TKLRAQSLAAAAAGGDSEVKEQLLTTMM-----SMTAKRSRRHLAQPSHAGASKAPSSVAMASSAVASALNLASVNGDTSVRRGGLSPLAPAGGPAPRKLTQRRLSEEAEYACRTAMGMLGGAQGGSSTGP
 CreinCPK17-3 AL--LAAAFAHFDTDGSGFITEDELRAALAAHHPAGE--GPDIGAIMAQFDIDGDGRIDYDEFLKMMVQADEGEDDEAGGEAEVLDVSSTPL-QPDDDGADAEGSREAAAAEAAAEAADRVGGCYVGVRPRSARPGNNRITAADVARLAAAAAGDPRATPQLASVAEAGG
 CreinCPK17-7 ----LAAAFAYFDADGSGYITVDELRSALAAHHPTGE--GPDIRALLSRVDADSDGRISYAEFLTMM--AAECMEEEVDLDQEE---------QEQDEGRSRSGQRRSKT----------LGGM---LKLRGANAVGQRSRASSHHSHHSHHIQSPHAQSHKVHGNEQQW
 CeinrCPK17-5 SHNPLAAAFAALDKDGSGFITADEIEAVLAAHHPNGRAHGPDVQAMLAAADTDADGRISYEEFLAMLLAAEDGAGDFLGGGGNGAPQAPAPAHQPESQSQPLPAPAQLQQIAGPQGQKHQAAEEQREAHSLSQPLPQVVAAAQPKSPVVKEPVVSCQAPAAPATAKPPLP
    CreinCPK2 ----VTGLLAEADLDKDGRLNYREF---CTMLLAAPGLEAGGSEADANAAEVQAALRLSGIDLDSA------GLPGALNGNAAAALPASAAAAVAAAVAAGFRPGSHSTNDTA---WDPSFGSGKRTSTGGGVRTSTGGGIRTSPSGVQASIGSRPQAPSPQRSGSLAAA
 CreinCPK17-6 -------AFAFMDADHDGFLTRAELVAYLGDGHTEL------AELIIAEADADCDGRVSADEFAAAY------NMTLERSRAASRPNTAPG--GGRTRSEQSDTEDGAG-----GGMGGAGGG--------------GGGVGGGKGPGG-VVRMTSVTSVVSMGDELGR
      VcCPK15 -------AFAFMDTDRNGYLTCGELVSYLGESHKEL------AELIIAEADTDLDGRISGEEFKAAY------AMTMDRSRATSRPNTATGSWGAGNGRASSGTAAAAGRPVGKGGSGSVEGGTTGMAVGEAGGQRLNGVAVDGGQDPNGTVVRMTSVTSVVSMGDELAQ
 CreinCPK17-2 DA--KE-LLATADTNGDGLIDYLEFCSMLRNNNEALKQSTRAIKRQYSKFF
      VcCPK17 DA--KE-LLATADTNGDGHIDYLEFCSMLRNNNEALKQSTRAIKRQYSKFF
     CsubCPK4 EE--IENMIDQFDTNHDGEIDYSEFLAMMRADNEDLKAASNYLRRRSSVCS
    CreinCPK9 KE--IDEIIDQVDQDGNGTIEYGEFVAMMRGL
   CreinCPK34 ----VDAILAQVDQNGDGRIDYEEFCAMMRATDLDVLKSAHEALKTKVVVKSVLARVQAEPMREDSITDMSRKSSRAMATAESRKQRGASATPANAEEGEQ
      VcCPK34 ----VDALLRQVDKNGDGRIDYEEFCLMMRASDLDVLKCAHEVRILEG
    CsubCPK34 QN--VEEVLKDVDKNQDGDIDYEEFCEMMRNMQSSQIKRTATNYRKGAIL
      PpCPK1-3 IS--VDELLHEVDQNNDGQIDYAEFVTMMRKGNPGAAGRSSFRNSQSLSLNDVLMMG
      PpCPK2-2 TS--IEDLIREVDQDNDGRIDYNEFVTMMRKGN-GAVGRTTLRNS--LSLSDALMNPQ
      PpCPK2-1 TS--IEDLIREVDQDNDGRIDYNEFVMMMRKGN-GAVGRTTLRNS--LSLSDALMNPQ
      PpCPK1-2 TS--IEELIREVDQDNDGRIDYNEFVTMMRKGN-GTVGRATLRNS--LSLSDALMHTN
      PpCPK1-1 TS--IEELIREVDQDNDGRIDYNEFVTMMRKGN-GTVGRATLRNS--LSLSDALMHTN
       SmCPK2 DL--LEDMLREIDQDNDGRIDYNEFVTMMRNGN-GGVGRGQMRNSLSL--RDVIMVG
       SmCPK1 EV--IEEMMREADQDNDGRIDYSEFVTMMRKGA-GGIGRKTMRNSLSITFRDLLTV
      PpCPK1-4 MN--LEDLMRDVDLDNDGRIDYQEFVAMMRKGTGTAPPQRGARMEPSFGFSAALQL
      PpCPK1-6 VL--IEDLLHEVDVDHDGTIDYKMFVTMMRKGN-GGVGHQTLRCT--LGITDVLAHDMT
      PpCPK1-5 VR--LEDLLHDVSIGADGSIDYKMFVTMMRKCN-GGMGHQNLRCT--LGITDVLTLEEQY
       PpCPK9 QA--IQEIIREVDTDNDGKIDYDEFVAMMRKGNPDTEDGVMVVPPRHR
     PpCPK17-4 QT--IQEIINEVDTDRDGRIDYDEFVAMMRKGNPITEDGGK---HRHR
     PpCPK17-1 ET--IQEIISEVDTDNDGRIDYDEFVAMMRKGNPGAENGGTVNKPRHR
     PpCPK17-3 ET--LQEIINEVDTDHDGRIDYDEFVAMMRKGNPGTEDGGNLKSPRHRW
     PpCPK17-5 DT--IQEIIDEVDTDNDGRIDYDEFVAMMRKGNPGAEEGEKHN-HRHRY
     PpCPK17-2 ET--INEIIREVDTDNDGRIDYDEFVAMMRKGNEVPQMTTR----RHRR
       SmCPK3 ----LTEIIKEVDTDNDGKIDYDEFVAMMRGAN-PPRTAAVAAKAAEKSSS
     SmCPK17-1 ED--AKDIIKEVDTDNDGRINYDEFCAMMLKRN-TPPAGAGHRRSINDLPVGIKK
     SmCPK17-2 ET--MKEILKEVDTDNDGKINYDEFVAMMTKGAAPSSEQQNNRRKGQELPVGAPRHRK
      PpCPK7-3 DV--VQGILLEVDVDKDGRISYEEFSAMMRRGTDWRKASRQYSRDRFNSLSMRLVREGSLNPDKSVAVR
      PpCPK7-4 DV--VQGILLEVDVDKDGRISYEEFSAMMRRGTDWRKASRQYSRDRFNSLSMRLVREGSLNPDKSVAVR
      PpCPK7-1 DV--VQAILLEVDIDKDGRISYEEFATMMRRGTDWRKASRQYSRDRFNSLSMRLLRDGSLNPDKIVAVR
      PpCPK7-2 DV--VQGILSEVDLDKDGRISYEEFATMMRRGTDWRKASRQYSRDRFNSLSMRLLRDGSLNPPSYSMR
       PpCPK13 DV--VQAILSEVDLDKDGRISYEEFAVMMRRGTDWRKASRQYSRDRFNSLSMRLLRDGSLNPPSYSMSSMR
       PpCPK30 DV--VQAILSEVDLDKDGRISYEEFAVMMRRGTDWRKASRQYSRDRFNSLSMRLLRDGSLNPPSYSMSSMR
       SmCPK13 EM--IDDILQEVDLDKDGKISYEEFASMMRTGTDWRKASRHYSRGRYNSLSIRLLRDGSLQSGNYEKR
        SmCPK7 EV--VADILAEVDADKDGLVSYEEFARVMRTGTDWRKASRRYSKGRFNSLSNKLIKDGSLTATA
     PpCPK16-2 GS--VETLLEEADVDGDKRISLPEFQKLLHQASFGSRTNTDHNRHRT
     PpCPK16-3 GS--VGTLLEEADSDKDGRISLSEFQKLLHQASFGSRTNTDHNRQRT
     PpCPK16-1 GS--MDSILVEADIDGDGKISLSEFQKLLRQASLGSRTN-EHHTLVTHNHRKC
     PpCPK16-4 GS--MDSFLGEADIDGDGRISLPEFQKLLRQASLGSRNNSEHQAPVSHNPRRREAHHHHVCSATKSYRRKSGIGTVWNISINSYKVYFGPLMSTIVGNFLGCRNQD
       SmCPK28 GS--LDTLLDEADADGDGRISLREFQKLLRQASLGSRTNHHHIQPHLKHPKLLEMHAPSPKISEESSQ
       SmCPK16 GS--LGTLLEEGDIDGDGRISLAEFQKLLRQASLGSRN
       MpCPK17 VD--ATNFLDEVDKNNDGRVDYDEFLAMMTKEDKPKFR
       OlCPK17 GD--IGELIASADTDGDGCIDFNEFMAMMKADK
       OlCPK19 GD--VTELIATADANGDGVIDFEEFMTAMSADSSAR
  CreinCPK20-2 EL--AREMVNEVDKNNDGRVDYAEFEKMMQS
       VcCPK12 EL--AREMVNEVDKNNDGRVDYAEFEKMMQS
    CreinCPK12 TA--AEAIFDEVDVDGDGFLTPAE-VQLLLPASLTRDEAAAMVQQADKDADGRVSRKELEEALVVSKHHSKRAAGLPEGEEEADDGAGGADGDGEEAEQAGKNTVP
        OlCPK3 RAAIVKACIREADIDGNGKIDMDEFASLLQADPTDDLDQYDSREISVSSSFDKD
     Consensus ..  ......evD.#.dGridy.Ef..m$r..........................................................................................................................................
```
